# Associations of family history of hypertension, genetic, and lifestyle risks with incident hypertension

**DOI:** 10.1038/s41440-025-02314-9

**Published:** 2025-08-13

**Authors:** Masato Takase, Takumi Hirata, Naoki Nakaya, Mana Kogure, Rieko Hatanaka, Kumi Nakaya, Ippei Chiba, Sayuri Tokioka, Kotaro Nochioka, Tomohiro Nakamura, Naho Tsuchiya, Hirohito Metoki, Michihiro Satoh, Akira Narita, Taku Obara, Mami Ishikuro, Hisashi Ohseto, Ippei Takahashi, Tomoko Kobayashi, Eiichi N. Kodama, Yohei Hamanaka, Masatsugu Orui, Soichi Ogishima, Satoshi Nagaie, Nobuo Fuse, Junichi Sugawara, Shinichi Kuriyama, Gen Tamiya, Atsushi Hozawa, Masayuki Yamamoto, the ToMMo investigators

**Affiliations:** 1 Graduate School of Medicine, Tohoku University, Sendai, Miyagi Japan; 2 Tohoku Medical Megabank Organization, Tohoku University, Sendai, Miyagi Japan; 3 Human Care Research Team, Tokyo Metropolitan Institute for Geriatrics and Gerontology, Itabashi-ku, Tokyo Japan; 4 Tohoku University Hospital, Tohoku University, Sendai, Miyagi Japan; 5 Kyoto Women’s University, Kyoto, Japan; 6 Tohoku Medical and Pharmaceutical University, Sendai, Japan; 7 International Research Institute of Disaster Science, Tohoku University, Sendai, Miyagi Japan; 8 Suzuki Memorial Hospital, Satonomori, Iwanumashi, Miyagi Japan; 9 RIKEN Center for Advanced Intelligence Project, Tokyo, Japan

**Keywords:** Family History, Hypertension, Lifestyle Risk, Polygenic Risk Score, Implemental hypertension

## Abstract

Family history of hypertension may reflect genetic and lifestyle factors. Genetic risk can be assessed using polygenic risk score (PRS); however, whether PRS can stratify hypertension risk when combined with family history and lifestyle information is unclear. This prospective cohort study included 9,001 hypertension-free individuals aged ≥20 years from the Tohoku Medical Megabank Community-Based Cohort Study. Participants were scored on lifestyle factors, including body mass index, urinary sodium-to-potassium ratio, physical activity, alcohol consumption, and smoking at recruitment. During the mean follow-up of 4.3 years, 2822 (31.4%) cases of hypertension occurred. High genetic risk and poor lifestyle were associated with increased hypertension risk. Compared with participants with low genetic risk, ideal lifestyle, and no family history, high genetic risk significantly increased hypertension risk, even among those with ideal lifestyle and no family history (relative risk [RR] 1.28 [95% confidence interval [CI] 1.11–1.46]). Participants with low PRS, ideal lifestyle, but with family history had increased hypertension risk (RR 1.32 [95%CI 1.11–1.57]). Poor lifestyle increased hypertension risk across most genetic risk groups, regardless of family history. Integrating PRS into models with family history and lifestyle risk significantly improved predictive accuracy (area under the curve: 0.671 for family history and lifestyle risk and 0.674 for PRS integrated; *P* for difference <0.05). Integrating PRS with lifestyle and family history enhances the stratification of individuals at high risk for hypertension.

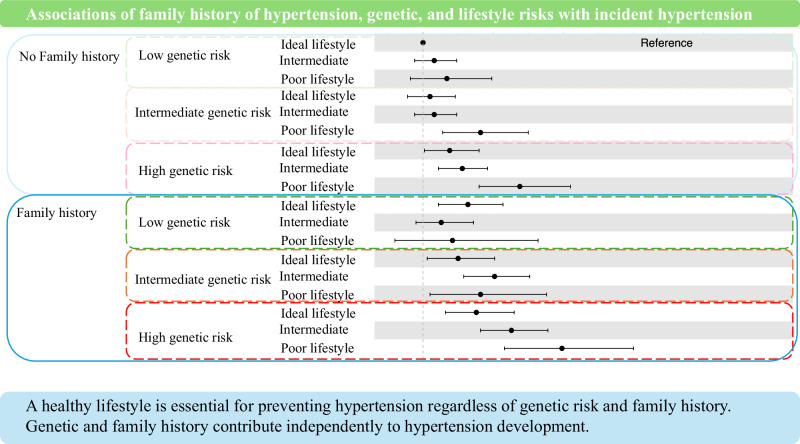

## Introduction

Hypertension is a leading cause of death and morbidity worldwide. According to the World Health Organization, 1.3 billion individuals live with hypertension, which contributes to over 10 million deaths annually [1]. Additionally, only 54% of adults with hypertension are diagnosed, 42% receive treatment, and 21% achieve hypertension control, underscoring its status as a critical public health challenge [1].

Hypertension arises from genetic and lifestyle factors [2]. Epidemiological studies have identified several lifestyle risk factors, including alcohol consumption, physical inactivity, obesity, smoking, and imbalanced sodium and potassium intake [3–6]. Furthermore, large-scale genome-wide association studies (GWASs) have identified over 900 genomic regions associated with hypertension [7–10]. Based on these findings, polygenic risk scores (PRS) have been developed from single nucleotide polymorphisms (SNPs) identified in GWASs. PRS has shown potential in predicting hypertension incidence [2, 11–13] and is expected to advance personalized prevention and treatment through disease-onset prediction and risk stratification.

Several prospective cohort studies have examined the combined effects of genetic susceptibility and adherence to a healthy lifestyle on hypertension incidence [2, 11, 14]. These studies showed that high genetic risk is associated with elevated blood pressure (BP) and hypertension, independent of lifestyle, while poor lifestyle is linked to elevated BP and hypertension, regardless of genetic risk [2, 11, 14]. However, only three studies in East Asia have explored this relationship: one prospective cohort study in China [14] and two cross-sectional studies in Japan [15, 16]. Given that allele frequencies and hypertension risk factor distributions differ by ancestry [17, 18], examining the combined effects of genetics and lifestyle on hypertension in specific populations remains essential.

Family history of hypertension is frequently used in clinical settings as a proxy for genetic risk. Family history of hypertension is associated with hypertension risk, regardless of other factors [19–21]. However, whether integrating PRS with lifestyle information can further stratify hypertension risk, beyond family history, remains unclear.

Therefore, this study aimed to examine the associations of a combined PRS and healthy lifestyle with hypertension in a large prospective cohort from the Tohoku Medical Megabank Community-Based Cohort (TMM CommCohort) Study [22]. Additionally, we assessed whether PRS could predict new-onset hypertension beyond lifestyle factors and family history of hypertension.

Point of view

- **Clinical relevance**
  Integrating PRS with lifestyle and family history may improve individualized risk stratification and early preventive interventions for hypertension in clinical settings.
- **Future direction**
  Multi-omics approaches may help clarify the mechanisms linking genetic risk and hypertension and identify novel intervention targets.
- **Consideration for the Asian population**
    PRS enables more refined stratification of hypertension risk in Asian populations, complementing rather than replacing the value of family history in clinical assessment.

## Methods

### TMM CommCohort study participants

This prospective cohort study included individuals aged ≥20 years living in Miyagi Prefecture, northeastern Japan, included in the TMM CommCohort study. Study details have been previously described [22, 23]. Briefly, the TMM CommCohort study (from May 2013 to March 2016) recruited >50,000 participants through three approaches: a type 1 survey (*n* = 40,433) conducted at municipal health check-up sites, a type 1 additional survey (*n* = 770), performed on different dates from the type 1 survey; and a type 2 survey (*n* = 13,855), conducted at assessment centers. Participants provided lifestyle and health-related information via blood and urine samples and a mailed self-reported questionnaire. All participants (*n* = 54,952) provided written informed consent.

Baseline Participants were invited to a follow-up assessment between June 2017 and March 2021. This survey collected detailed information similar to the baseline survey [24]. The Institutional Review Board of the Tohoku Medical Megabank Organization approved this study (approval number: 2022-4-047; approval date: June 30, 2022).

The analysis included participants from Type 1 surveys, as type 2 participants were previously used to construct the PRS [15]. Exclusion criteria were: (1) participants who withdrew by November 16, 2021 (*n* = 770); (2) history of cardiovascular disease (*n* = 1806); (3) hypertension diagnosis at baseline (*n* = 13,849); (4) participants lacking genetic data genotyped via the Affymetrix Axiom Japonica Array (*n* = 4374); (5) urinary sodium-to-potassium (Na/K) ratio values of infinity (*n* = 53); (6) genetic principal component values exceeding six standard deviations from the mean (*n* = 30); and (7) lack of follow-up BP data (*n* = 10,550). After these exclusions, a total of 9001 out of 19,581 eligible participants with follow-up blood pressure data were included in the final analysis (follow-up rate: approximately 46%).

### Healthy lifestyle factors and family history

A healthy lifestyle score was constructed based on five well-established hypertension risk factors: body mass index (BMI), alcohol consumption, smoking, physical activity, and Na/K ratio [3–6]. Detailed definitions are provided in the Supplementary Methods. Overall lifestyle was categorized as ideal ( ≥4 ideal lifestyle factors), poor ( ≤1 poor lifestyle factors), or intermediate (2–3 ideal lifestyle factors). Family history of hypertension was defined based on self-reported hypertension in biological parents or siblings.

### BP measurement and ascertainment of hypertension

At baseline, BP was measured during municipal health checkups. Per Ministry of Health, Labor and Welfare guidelines, BP was measured on the right upper arm after urination, following at least 5 min of seated rest, and avoiding activities that could affect BP, including exercise, diet, or smoking. Two measurements were taken, with the average used for analysis; a single measurement was accepted if site conditions required it [3, 25].

At follow-up, a trained nurse measured BP twice on the right upper arm using a digital automatic BP monitor (HEM-9000AI; Omron Healthcare Co., Ltd., Kyoto, Japan) at the community support center. Measurements were taken after ≥2 min of seated rest, and the mean value of the two readings was used for analysis. Hypertension was defined as BP ≥ 140/90 mmHg during the follow-up survey or self-reported antihypertensive treatment.

### PRS derived from BioBank Japan (BBJ)

Details on genotyping and quality control in this study are provided in the Supplementary Method. The PRS was calculated using summary statistics from a previous GWAS for systolic BP (SBP) in the BBJ, publicly available through the National Bioscience Database Center [26]. Study participants were independent of BBJ. To exclude non-autosomal sex effects, SNPs on the X and Y chromosomes were removed. PLINK 1.9 was used to calculate the PRS via the clumping and thresholding method. Based on a previous study [15], clumping was conducted to capture the appropriate level of causal signals using the following parameters: --clump-p1 1 --clump-r2 0.1 –clump-kb 250. The PRS constructed with *P* < 0.001 was selected as it provided the best model fit for an independent Japanese population.

### Statistical analysis

Missing data were imputed using random forest imputation via missForest R packages. Data are presented as means (SD) or median (interquartile range) for continuous variables and number (percentage) for categorical variables.

First, participants were classified based on their PRS tertile to analyze the potential association between PRS and hypertension. Poisson regression with robust variance correction was used to estimate relative risk (RR) and 95% confidence intervals (CI) for hypertension incidence. The same model was used to analyze associations between hypertension and lifestyle factors or family history of hypertension. Second, we examined the association between the combination of family history and lifestyle factors and hypertension incidence, using individuals with no family history and an ideal lifestyle as the reference group. Third, joint exposure risk ratios for genetic and lifestyle factors were evaluated, with the reference group being those in the lowest PRS tertile and ideal lifestyle. Fourth, the combined effects of family history, PRS tertiles, and lifestyle risks were examined using 18 categories. Poisson regression with robust variance correction was employed to estimate RR and 95%CI, with the reference group being those in the lowest PRS tertile, ideal lifestyle, and without family history of hypertension. Models were adjusted for age at inclusion, sex, education level, and the first 10 genetic principal components to account for population structure.

To compare the predictive performance of hypertension incidence, the area under the receiver operating curve (AUROC) and 95% CI were calculated using logistic regression analysis. The AUROC was compared using the Delong test.

As sensitivity analyses, first we analyzed covariance and estimated the adjusted least-square means of SBP for 9 categories by genetic and lifestyle risk, and 18 categories by genetic and lifestyle risk, and family history of hypertension. Second, we performed analyses stratified by age (median age, 60.0 years) were performed, as younger participants may have reported the absence of hypertension in their family, indicating no family history. Third, to confirm the robustness of our findings, we performed sensitivity analyses using additional P-value thresholds: 5 × 10⁻⁸, 0.01, 0.05, 0.1, 0.2, 0.3, 0.4, and 0.5. Finally, we conducted stratified analysis by sex.

Inverse probability weighting was applied in all analyses to mitigate selection bias from including only participants who completed both the baseline and follow-up survey. Statistical significance was set at *P* < 0.05 (two-sided). Analyses were performed using R version 4.1.2 (R Foundation for Statistical Computing, Vienna, Austria).

## Results

### Characteristics of participants

A total of 9001 participants were included in the analysis. During the mean 4.3 years follow-up, 2822 (31.4%) cases were identified. Participants had a mean (SD) age of 57.9 (11.8) years, systolic BP of 117.8 (12.6) mmHg, diastolic BP of 71.4 (8.8) mmHg, BMI of 22.5 (3.2) kg/m², and a urinary sodium-to-potassium (Na/K) ratio of 4.0 (1.0). The proportion (%) of participants were women, obese, current drinkers, current smokers, physically inactive, and had high Na/K ratio ( >4.0) were 6318 (70.2%), 1650 (18.3%), 4,501 (50.0%), 1060 (11.8%), 3797 (42.2%), and 4343 (48.3%), respectively (Table 1). Women tended to have healthier lifestyles than men. Higher PRS groups included a greater proportion of participants with family history of hypertension. Supplementary Table 1 shows the participants’ characteristics according to family history, genetic risk, and lifestyle risk.

**Table 1:** Characteristics of study participants according to the genetic and lifestyle risk

Genetic risk	Low			Intermediate			High			Overall
Lifestyle score	Ideal	Intermediate	Poor	Ideal	Intermediate	Poor	Ideal	Intermediate	Poor	
Number	1252	1533	216	1066	1649	285	979	1711	310	9001
Age, years	60.2 (10.6)	60.2 (10.6)	58.3 (11.9)	57.6 (11.8)	59.4 (11.2)	57.1 (12.1)	56.3 (12.8)	58.5 (10.9)	56.1 (12.4)	57.9 (11.8)
Women, %	1072 (85.6)	1072 (85.6)	894 (58.3)	59 (27.3)	959 (90.0)	1072 (65.0)	106 (37.2)	889 (90.8)	1146 (67.0)	6318 (70.2)
SBP, mmHg	116.7 (12.7)	116.7 (12.7)	117.5 (12.5)	120.9 (11.7)	116.4 (12.9)	117.7 (12.7)	119.9 (12.0)	117.5 (12.6)	118.4 (12.4)	117.8 (12.6)
DBP, mmHg	70.1 (8.7)	71.6 (8.8)	75.1 (8.6)	70.0 (8.7)	71.6 (8.8)	73.7 (8.6)	70.7 (8.6)	71.9 (8.8)	74.6 (8.3)	71.4 (8.8)
Smoking Status, %										
Never smoker	1136 (90.7)	764 (49.8)	12 (5.6)	1019 (95.6)	959 (58.2)	34 (11.9)	952 (97.2)	1021 (59.7)	37 (11.9)	5934 (65.9)
Ex-smoker	70 (5.6)	504 (32.9)	132 (61.1)	31 (2.9)	466 (28.3)	176 (61.8)	13 (1.3)	437 (25.5)	178 (57.4)	2007 (22.3)
Current smoker	46 (3.7)	265 (17.3)	72 (33.3)	16 (1.5)	224 (13.6)	75 (26.3)	14 (1.4)	253 (14.8)	95 (30.6)	1060 (11.8)
Drinking Status, %										
Never drinker	1092 (87.2)	711 (46.4)	22 (10.2)	842 (79.0)	499 (30.3)	13 (4.6)	730 (74.6)	376 (22.0)	8 (2.6)	4293 (47.7)
Ex-drinker	4 (0.3)	48 (3.1)	11 (5.1)	3 (0.3)	41 (2.5)	10 (3.5)	8 (0.8)	62 (3.6)	20 (6.5)	207 (2.3)
Current drinker	156 (12.5)	774 (50.5)	183 (54.7)	221 (20.7)	1109 (67.3)	262 (91.9)	241 (24.6)	1273 (74.4)	282 (91.0)	4501 (50.0)
Moderate intensity physical activity, min/week	180.0 [129.6, 262.3]	129.6 [49.3, 203.6]	75.0 [37.2, 137.1]	171.5 [124.5, 259.1]	128.1 [52.3, 202.5]	94.3 [36.2, 138.0]	177.3 [126.0, 268.4]	132.0 [50.1, 204.6]	75.0 [32.0, 129.6]	142.5 [62.1, 229.3]
Vigorous intensity physical activity, min/week	49.3 [30.0, 120.0]	30.0 [6.0, 120.0]	30.0 [0.0, 33.1]	49.3 [30.0, 120.0]	30.0 [6.0, 120.0]	30.0 [0.0, 39.5]	42.7 [30.0, 120.0]	30.0 [4.6, 120.0]	30.0 [0.0, 33.0]	30.0 [19.3, 120.0]
BMI, kg/m^2^	21.6 (2.5)	22.7 (3.2)	25.2 (3.3)	21.4 (2.5)	22.7 (3.3)	25.0 (3.4)	21.6 (2.5)	22.5 (3.2)	25.3 (3.5)	22.5 (3.2)
Na/K ratio	3.7 (0.9)	4.2 (1.0)	4.7 (0.9)	3.6 (0.9)	4.2 (1.0)	4.7 (0.9)	3.6 (0.8)	4.2 (1.0)	4.7 (0.9)	4.0 (1.0)
Estimated sodium intake, g/day	9.0 (2.0)	9.6 (2.0)	10.4 (2.0)	8.9 (2.0)	9.6 (2.1)	10.3 (2.1)	8.8 (1.9)	9.5 (2.1)	10.3 (2.1)	9.4 (2.1)
Family history, %	403 (32.2)	403 (32.2)	499 (32.6)	61 (28.2)	338 (31.7)	545 (33.1)	97 (34.0)	394 (40.2)	620 (36.2)	3068 (34.1)
Education levels, %										
Below high school	779 (62.2)	926 (60.4)	138 (63.9)	664 (62.3)	1045 (63.4)	182 (63.9)	597 (61.0)	1068 (62.4)	211 (68.1)	5610 (62.3)
Vocational school or junior college or technical college	337 (26.9)	370 (24.1)	37 (17.1)	318 (29.8)	389 (23.6)	58 (20.4)	298 (30.4)	432 (25.2)	64 (20.6)	2303 (25.6)
University or graduate school	119 (9.5)	230 (15.0)	41 (19.0)	75 (7.0)	209 (12.7)	43 (15.1)	77 (7.9)	195 (11.4)	33 (10.6)	1022 (11.4)
Others	17 (1.4)	7 (0.5)	0 (0.0)	9 (0.8)	6 (0.4)	2 (0.7)	7 (0.7)	16 (0.9)	2 (0.6)	66 (0.7)
Obesity, %	8 (1.1)	64 (5.1)	342 (22.3)	133 (61.6)	45 (4.2)	332 (20.1)	179 (62.8)	48 (4.9)	316 (18.5)	1650 (18.3)
High Na/K ratio	332 (26.5)	940 (61.3)	192 (88.9)	252 (23.6)	949 (57.6)	250 (87.7)	200 (20.4)	957 (55.9)	271 (87.4)	4343 (48.3)
Insufficient regular physical activity, %	239 (19.1)	771 (50.3)	170 (78.7)	219 (20.5)	852 (51.7)	221 (77.5)	210 (21.5)	874 (51.1)	241 (77.7)	3797 (42.2)

Obesity is defined as BMI ≥25.0 kg/m^2^ based on the Western Pacific Region of World Health Organization criteria in Japanese individuals

The high Na/K ratio is defined as ≥4.0

Insufficient regular physical activity is defined as not meeting the American Heart Association recommendations of at least 150 min of moderate activity per week or 75 min of vigorous activity per week

*BMI* body mass index, *DBP* diastolic blood pressure, *Na/K ratio* sodium-to-potassium ratio, *SBP* systolic blood pressure

Participants who completed the follow-up survey were older, had a higher proportion of women, non-obesity, never-smokers, and engaged in regular physical activity than those who were not followed up (Supplementary Table 2).

### Association of lifestyle with hypertension incidence

Poor lifestyle was associated with higher hypertension risk. Compared with the ideal lifestyle group, the multivariable-adjusted RRs (95%CIs) for hypertension risk were 1.08 (1.01–1.16) and 1.33 (1.19–1.48) for the intermediate and poor lifestyle groups, respectively.

### Association of the PRS with hypertension incidence

Higher PRS was associated with higher hypertension risk. The multivariate RRs (95%CIs) for low (reference), intermediate, and high were 1.00, 1.08 (0.99–1.17), and 1.27 (1.18–1.36), respectively.

### Associations of family history and lifestyle risk combination with Hypertension Incidence

When family history and lifestyle categories were combined, poor lifestyle was associated with higher hypertension risk. Family history was associated with a higher hypertension risk among most lifestyle categories (Supplementary Table 3).

### Associations of genetic and lifestyle risk combination with Hypertension Incidence

When genetic risk and lifestyle categories were combined, higher genetic risk was associated with higher hypertension risk. Poor lifestyle was associated with a higher hypertension risk among most genetic risk groups (Table 2). Compared with participants with low genetic risk and an ideal lifestyle, those with high genetic risk and an ideal lifestyle had a significantly higher hypertension risk (RR 1.16 [95%CI, 1.02–1.31). Participants with high genetic risk and poor lifestyles had the highest hypertension risk (RR 1.65 [95%CI, 1.41–1.93]).

**Table 2:** Association between genetic and lifestyle risk with hypertension incidence

Genetic risk	Lifestyle score	Hypertension/number of participants	%	RR, 95% CI	
Low	Ideal ( ≤1 poor factors)	367/1252	(29.3)	Ref	
	Intermediate (2–3 poor factors)	455/1533	(29.7)	1.01	(0.90–1.14)
	Poor ( ≥4 poor factors)	69/216	(31.9)	1.10	(0.89–1.36)
Intermediate	Ideal ( ≤1 poor factors)	303/1066	(28.4)	1.01	(0.89–1.15)
	Intermediate (2–3 poor factors)	500/1649	(30.3)	1.10	(0.98–1.23)
	Poor ( ≥4 poor factors)	105/285	(36.8)	1.32	(1.10–1.57)
High	Ideal ( ≤1 poor factors)	310/979	(31.7)	1.16	(1.02–1.31)
	Intermediate (2–3 poor factors)	575/1711	(33.6)	1.28	(1.14–1.42)
	Poor ( ≥4 poor factors)	138/310	(44.5)	1.65	(1.41–1.93)

Bold shows are statistically significant

The model is adjusted for age, sex, and the first ten principal components

Hypertension is defined as systolic/diastolic blood pressure of 140/90 mmHg or higher measured at a community-support center and/or self-reported treatment for hypertension

Lifestyle is categorized as ideal (having at least three ideal lifestyle factors), poor (having 0–1 ideal lifestyle factors), intermediate (having 2–3 ideal lifestyle factors)

Lifestyle includes the following factors: obesity, defined as BMI ≥ 25.0 kg/m^2^ based on the Western Pacific Region of World Health Organization criteria in Japanese individuals, high Na/K ratio, defined as ≥4.0, insufficient regular physical activity, defined as not meeting the American Heart Association recommendations of at least 150 min of moderate activity per week or 75 min of vigorous activity per week, smoking, defined as ex-smoker or current smoker, drinking, defined as ex-drinker or current drinker

Genetic risk is classified based on the tertile of polygenic risk score

*CI* confidence interval, *RR* relative risk

### Associations of family history of hypertension, genetic and lifestyle risk with Hypertension Incidence

Initially, we examined the association between family history of hypertension and hypertension. Participants with family history of hypertension had significantly higher hypertension risk than those without (RR, 1.23 [95%CI, 1.16–1.31] in the multivariable-adjusted model.

Subsequently, we evaluated the combined associations of family history, genetic risk, and lifestyle risk with hypertension (Table 3). Family history was significantly associated with hypertension risk, even among those with low genetic risk and an ideal lifestyle (RR, 1.32 [95%CI, 1.11–1.57]). Among participants with an ideal lifestyle and no family history of hypertension, a high genetic risk was significantly associated with hypertension risk (RR, 1.19 [95%CI, 1.01–1.40]). Participants with high genetic risk, poor lifestyle, and family history of hypertension had the highest hypertension risk (RR, 1.99 [95%CI, 1.58–2.50]).

**Table 3:** Association between genetic and lifestyle risk with hypertension incidence

Family history	Genetic risk	Lifestyle score	Hypertension/number of participants	%	RR, 95% CI	
No	Low	Ideal ( ≤1 poor factors)	235/849	(27.7)	Ref	
		Intermediate (2–3 poor factors)	319/1034	(30.9)	1.08	(0.94–1.24)
		Poor ( ≥4 poor factors)	52/155	(33.5)	1.17	(0.91–1.49)
	Intermediate	Ideal ( ≤1 poor factors)	199/728	(27.3)	1.05	(0.89–1.23)
		Intermediate (2–3 poor factors)	312/1104	(28.3)	1.08	(0.94–1.24)
		Poor ( ≥4 poor factors)	71/188	(37.8)	1.41	(1.14–1.75)
	High	Ideal ( ≤1 poor factors)	179/585	(30.6)	1.19	(1.01–1.40)
		Intermediate (2–3 poor factors)	351/1091	(32.2)	1.28	(1.11–1.46)
		Poor ( ≥4 poor factors)	90/199	(45.2)	1.69	(1.40–2.05)
Yes	Low	Ideal ( ≤1 poor factors)	132/403	(32.8)	1.32	(1.11–1.57)
		Intermediate (2–3 poor factors)	136/499	(27.3)	1.13	(0.95–1.36)
		Poor ( ≥4 poor factors)	17/61	(27.9)	1.21	(0.80–1.82)
	Intermediate	Ideal ( ≤1 poor factors)	104/338	(30.8)	1.25	(1.03–1.51)
		Intermediate (2–3 poor factors)	188/545	(34.5)	1.51	(1.29–1.76)
		Poor ( ≥4 poor factors)	34/97	(35.1)	1.41	(1.05–1.88)
	High	Ideal ( ≤1 poor factors)	131/394	(33.2)	1.38	(1.16–1.65)
		Intermediate (2–3 poor factors)	224/620	(36.1)	1.63	(1.41–1.89)
		Poor ( ≥4 poor factors)	48/111	(43.2)	1.99	(1.58–2.50)

The model is adjusted for age, sex, and the first ten principal components

Hypertension is defined as systolic/diastolic blood pressure of 140/90 mmHg or higher measured at a community-support center and/or self-reported treatment for hypertension

Lifestyle is categorized as ideal (having at least three ideal lifestyle factors), poor (having 0–1 ideal lifestyle factors), intermediate (having 2–3 ideal lifestyle factors)

Lifestyle score includes the following factors: obesity, defined as BMI ≥ 25.0 kg/m^2^ based on the Western Pacific Region of World Health Organization criteria in Japanese individuals, high Na/K ratio, defined as ≥4.0, insufficient regular physical activity, defined as not meeting the American Heart Association recommendations of at least 150 min of moderate activity per week or 75 min of vigorous activity per week, smoking, defined as ex-smoker or current smoker, drinking, defined as ex-drinker or current drinker

Genetic risk is classified based on the tertile of polygenic risk score

*CI* confidence interval, *RR* relative risk

The AUROC values (95%CI) for family history, PRS, and lifestyle were 0.668 (0.656–0.679), 0.668 (0.657–0.679), and 0.666 (0.654–0.677), respectively (Table 4). The AUROC for integrated family history and lifestyle information was 0.671 (0.659–0.685). Integrating PRS to the model that included lifestyle and family history significantly improved the AUROC to 0.674 (0.663–0.685) compared to the model including lifestyle and family history (P for difference = 0.04).

**Table 4:** Area under the receiver operating characteristic curve for family history, genetic, and lifestyle risk to predict hypertension incidence

	Area under the receiver operating characteristic curve (95% CI)
	Hypertension
Model 1^a^	0.663 (0.651–0.674)
Model 1 + lifestyle score risks	0.666 (0.654–0.677)
Model 1 + PRS	0.668 (0.657–0.679)
Model 1 + family history	0.668 (0.656–0.679)
Model 1 + PRS + lifestyle score	0.670 (0.661–0.684)
Model 1 + family history + PRS	0.673 (0.661–0.684)
Model 1 + family history + lifestyle score	0.671 (0.659–0.685)
Model 1 + family history + lifestyle score + PRS	0.674 (0.663–0.685)

The area under the receiver operating characteristics curve is calculated using the multivariable logistic regression model

Hypertension is defined as systolic/diastolic blood pressure 140/90 mmHg or higher measured at a community-support center and/or self-reported treatment for hypertension

Lifestyle score includes the following factors: obesity, defined as BMI ≥ 25.0 kg/m^2^ based on the Western Pacific Region of World Health Organization criteria in Japanese individuals, high Na/K ratio, defined as ≥4.0, insufficient regular physical activity, defined as not meeting the American Heart Association recommendations of at least 150 min of moderate activity per week or 75 min of vigorous activity per week, smoking, defined as ex-smoker or current smoker, drinking, defined as ex-drinker or current drinker

Genetic risk is classified based on the tertile of polygenic risk score

*CI* confidence interval, *PRS* polygenic risk score

^a^Model 1 includes age, sex, education status, and the first 10 principal components as predictor variables

Supplementary Table 3 shows the adjusted least-square means of SBP, showing that both PRS and lifestyle risk correlated with SBP, with higher SBP observed in participants with a family history of hypertension. When stratified by age, similar association patterns were observed, although these were more apparent in participants below the median age group ( <60 years) and appeared attenuated in those above the median age group (Supplementary Tables 4–5). We performed analyses using additional *P*-value thresholds, but the results were substantially unchanged (Supplementary Tables 6–13). The association tended to similar both men and women (Supplemental Tables 14–15).

## Discussion

This study showed that in a general sample of approximately 9000 Japanese adults, participants with a higher PRS had a higher hypertension risk, irrespective of lifestyle or family history. Similarly, a poor lifestyle was consistently associated with increased hypertension risk across all PRS levels and family history categories. Notably, family history of hypertension was linked to a significantly higher hypertension risk, even among participants with low PRS and an ideal lifestyle. Incorporating PRS into models that included family history and lifestyle improved the predictive ability for hypertension incidence.

Previous studies conducted in the UK and China showed that genetic risk is associated with a higher risk of hypertension, while a healthy lifestyle is linked to a reduced risk [2, 11, 14]. The current study confirmed these associations in a Japanese population. Given that most genetic research has been conducted in individuals of European ancestry, and that PRSs tend to have lower predictive performance in underrepresented populations—such as East Asian, African, and Hispanic/Latino populations [27], these findings may contribute to the decreased genomic medical disparity between ancestry.

A family history of hypertension is often used as a surrogate for genetic risk. The Framingham Heart Study found that parental early-onset hypertension (age <55 years) was associated with hypertension incidence in offspring [19]. Similarly, other studies have shown that family history of hypertension is linked to elevated BP [20, 21]. With advancements in genomic science, PRSs now enable the direct assessment of genetic risk, providing a more precise alternative to family history as a surrogate indicator. Previous studies showed that higher genetic risk was associated with hypertension incidence independently of traditional risk factors, excluding family history [2, 11–13]. However, whether PRS and family history independently contribute to hypertension risk remains unclear. The current study showed that even among participants with low genetic risk and an ideal lifestyle, family history of hypertension was associated with a higher hypertension risk. Furthermore, a higher genetic risk was associated with a higher hypertension risk, even among participants with an ideal lifestyle and no family history of hypertension. Family history may reflect not only inherited genetic factors but also shared environmental exposures, epigenetic modifications, and gene–environment interactions [28]. It is also possible that the apparent independence between PRS and family history arises in part from limitations in current measures. PRS accounts for only a portion of the total heritability of hypertension, and our assessment of lifestyle factors was relatively broad (e.g., current smoking and ex-smoking vs. non-smoking), potentially missing more nuanced environmental influences. However, family history may thus serve as a surrogate for complex and unmeasured genetic and environmental factors that are not fully captured by current PRS or lifestyle measurements. These findings underscore the importance of careful BP monitoring in individuals with family history of hypertension and/or high genetic risk. As effective prevention strategies for these populations remain unclear, further studies are required to elucidate underlying mechanisms and identify potential intervention targets.

The predictive ability for hypertension was similar between models using family history alone and those using PRS alone, whereas the model based solely on lifestyle factors showed the lowest discrimination. A model combining family history and lifestyle—both of which are readily available in clinical practice—outperformed the model combining lifestyle and PRS. However, the model integrating family history, lifestyle, and PRS achieved the highest predictive performance, although the improvement was modest. These findings are consistent with previous reports demonstrating that the effects of family history and PRS are largely independent across various diseases, including cancer, diabetes, hypercholesterolemia, coronary artery disease, gout, glaucoma, and asthma [28–32]. The integration of PRS and family history has been shown to improve predictive accuracy, likely because they capture different but complementary aspects of genetic susceptibility. While PRS provides a direct estimate of inherited genetic risk, family history may reflect shared environmental exposures, epigenetic mechanisms, and gene–environment interactions [28]. Although the addition of PRS led to a statistically significant improvement in AUROC, the overall gain in discrimination was limited. Given that BP can be measured non-invasively and easily in routine clinical settings, the clinical need for highly discriminative risk models for hypertension may be inherently limited. Therefore, the clinical value of PRS may lie not in enhancing short-term risk prediction, but in enabling early identification of genetically predisposed individuals and promoting timely intervention before BP elevation becomes clinically apparent.

This study showed that an ideal lifestyle lowers the risk of hypertension, regardless of genetic risk or family history. This finding supports the idea that healthy behaviors can mitigate inherited susceptibility. A plausible mechanism is that genetic variants influence key physiological systems—such as hormonal regulation and vascular function—in ways that lifestyle changes cannot fully reverse. However, individuals with high genetic risk or a family history of hypertension still faced increased risk, even when maintaining ideal lifestyles. GWASs have identified BP–related variants in genes regulating the renin–angiotensin–aldosterone system (RAAS) and the sympathetic nervous system [7, 33]. These systems control vascular tone, fluid balance, and hormonal responses. Genetic disruptions in these pathways may cause lasting physiological changes that lifestyle interventions alone cannot fully counteract. This could explain why some individuals remain vulnerable despite favorable behaviors. Several circulating metabolites, such as branched-chain amino acids and lipid species, have also been linked to blood pressure and hypertension risk [34, 35]. Yet the mechanisms connecting genetic variants to these metabolic patterns remain unclear. Certain metabolites may act as intermediates, transmitting genetic influences into physiological states that elevate blood pressure, independent of lifestyle. Although our study did not examine molecular mediators directly, future work should aim to identify specific metabolites that bridge genetic risk and hypertension. Mediation analysis using multi-omics data—particularly genomics and metabolomics—could help quantify the role of these intermediates. Clarifying these pathways may also reveal targets for therapies that address residual genetic risk through metabolic intervention [36–38]. Therefore, even individuals with ideal lifestyles may require continued BP monitoring, including home measurements, if they carry high genetic risk or a family history of hypertension.

The associations of family history, genetic risk, and lifestyle with hypertension incidence showed similar trends in both men and women. X-linked genes, however, may also influence blood pressure regulation, and sex-specific differences in genetic susceptibility cannot be ruled out. A case–control study in a northeastern Chinese Han population identified significant associations between hypertension and polymorphisms in three X-linked genes: *ACE2*, *AGTR2*, and *apelin* [39]. Although these associations were observed in both sexes, the location of these genes on the X chromosome suggests that gene dosage or X-inactivation may contribute to individual susceptibility. Future research that includes sex chromosome analysis may help clarify the role of sex-linked genetic variation in hypertension.

This study had several strengths. To our knowledge, this is the first study to quantitatively evaluate the combined impact of family history of hypertension, polygenic risk, and lifestyle on the incidence of hypertension in a general population. Its prospective design minimizes concerns about retrospective reporting bias and reverse causality. Furthermore, this is the first study to explore the joint impact of genetic and lifestyle risks on hypertension incidence in Japan.

However, our study had some limitations. First, we analyzed participants who completed both the baseline and follow-up surveys, which may have introduced selection bias. To address this, we applied inverse probability weighting. Second, our study population included only Japanese participants. Given well-documented differences in linkage disequilibrium, allele frequency, and hypertension risk factors across ancestries [17, 18], our findings may not be generalizable to other populations. Third, the PRS in this study included only 1786 SNPs, which cannot fully explain hypertension heritability. Rare variants were also excluded. Therefore, our PRS may not fully capture individual genetic risk, which might explain why family history could not be entirely accounted for. Future GWASs, exome sequencing, and whole-genome sequencing may identify additional hypertension-associated variants, allowing for a more precise genetic risk assessment. Further studies are necessary to determine the usefulness of genetic information in conjunction with family history information. Fourth, we defined hypertension based on office BP. Home BP is superior to office measurement as a predictor of cardiovascular disease. We previously examined the association between PRS and prevalence of hypertension, but it might be useful to examine the association with the new onset of home hypertension [40, 41]. Fifth, family history of hypertension may be misclassified, particularly among younger participants. Younger individuals may not be aware of their parents’ hypertension status if their parents have not yet been diagnosed or have not disclosed their condition. This could lead to underreporting of family history and attenuation of the observed association. Therefore, caution is needed when interpreting the strength of the association between family history and incident hypertension, especially in younger populations. Finally, participants who undergo health check-ups may have higher health consciousness than those who do not [42], which could have introduced volunteer bias in our study. However, this potential bias is likely to have been evenly distributed across participants, and we therefore consider that the internal validity of the study is preserved. In addition, in some subgroups—such as participants with low genetic risk, no family history, and an ideal lifestyle—the expected risk gradient was not clearly observed, despite a relatively high incidence of hypertension. This may reflect misclassification bias in lifestyle and family history variables, both of which were assessed using broad categories. Moreover, the PRS used in this study explains only part of the heritability. These measurement limitations may have attenuated true associations in certain subgroups.

## Perspective of Asia

This study demonstrates that both polygenic risk scores and family history are independently associated with the risk of developing hypertension in an Asian population. Given that genetic architecture and lifestyle patterns differ substantially between Asian and Western populations [17, 18], our findings highlight the importance of evaluating both genetic and familial risk alongside lifestyle factors to enable meaningful risk stratification and personalized prevention strategies in Asia.

## Conclusion

Our study quantitatively estimated the associations of family history of hypertension, genetic, and lifestyle risks with hypertension incidence in the general Japanese population. The findings showed that poor lifestyle was associated with higher hypertension risk regardless of genetics and family history of hypertension. This finding supports the notion that a healthy lifestyle is essential for preventing hypertension. Family history of hypertension was associated with elevated risk, even among individuals with a low PRS and ideal lifestyle. Similarly, a higher PRS was linked to increased risk, even among those without a family history and with a healthy lifestyle. These findings suggest that genetic and familial risk factors contribute independently to hypertension development. Integrating PRS with lifestyle and family history information improved the predictive ability for hypertension incidence, but modestly. From a clinical perspective, individuals identified as having both high genetic risk and a family history of hypertension should be encouraged to monitor their blood pressure regularly, regardless of their lifestyle. In such individuals, early preventive measures—such as lifestyle counseling, health education, and more frequent monitoring—may help delay or prevent the onset of hypertension. Importantly, incorporating PRS into clinical or public health settings may help identify individuals at high risk who might otherwise be overlooked by traditional assessment methods, thereby serving as a complementary tool for developing personalized prevention strategies.

## Supplementary information

Supplementary Online Content

## Data Availability

The authors, Masato Takase and Atsushi Hozawa have full access to all data in the study and take responsibility for the integrity and accuracy of the data analysis.

## References

[1] World Health Organization. Global report on hypertension: the race against a silent killer. Geneva, Switzerland: World Health Organization. 2023:1–276.

[2] Pazoki R, Dehghan A, Evangelou E, Warren H, Gao H, Caulfield M, et al. Genetic predisposition to high blood pressure and lifestyle factors: associations with midlife blood pressure levels and cardiovascular events. Circulation. 2018;137:653–61. 29254930 10.1161/CIRCULATIONAHA.117.030898

[3] Umemura S, Arima H, Arima S, Asayama K, Dohi Y, Hirooka Y, et al. The Japanese Society of Hypertension guidelines for the management of hypertension (JSH 2019). Hypertens Res. 2019;42:1235–481. 31375757 10.1038/s41440-019-0284-9

[4] Whelton PK, Carey RM, Aronow WS, Casey DE Jr, Collins KJ, Dennison Himmelfarb C, et al. ACC/AHA/AAPA/ABC/ACPM/AGS/APhA/ASH/ASPC/NMA/PCNA Guideline for the prevention, detection, evaluation, and management of high blood pressure in adults: a report of the American College of Cardiology/American Heart Association Task Force on Clinical Practice Guidelines. Hypertension. 2018;71:e13–e115. 29133356 10.1161/HYP.0000000000000065

[5] Williams B, Mancia G, Spiering W, Agabiti Rosei E, Azizi M, Burnier M, et al. 2018 ESC/ESH Guidelines for the management of arterial hypertension. Eur Heart J. 2018;39:3021–104. 30165516 10.1093/eurheartj/ehy339

[6] US Preventive Services Task Force, Krist AH, Davidson KW, Mangione CM, Cabana M, Caughey AB, et al. Screening for hypertension in adults: US Preventive Services Task Force reaffirmation recommendation statement. JAMA. 2021;325:1650–6. 33904861 10.1001/jama.2021.4987

[7] International Consortium for Blood Pressure Genome-Wide Association Studies, Ehret GB, Munroe PB, Rice KM, Bochud M, Johnson AD, et al. Genetic variants in novel pathways influence blood pressure and cardiovascular disease risk. Nature. 2011;478:103–9. 21909115 10.1038/nature10405 PMC3340926

[8] Evangelou E, Warren HR, Mosen-Ansorena D, Mifsud B, Pazoki R, Gao H, et al. Genetic analysis of over 1 million people identifies 535 new loci associated with blood pressure traits. Nat Genet. 2018;50:1412–25. 30224653 10.1038/s41588-018-0205-x PMC6284793

[9] Giri A, Hellwege JN, Keaton JM, Park J, Qiu C, Warren HR, et al. Trans-ethnic association study of blood pressure determinants in over 750,000 individuals. Nat Genet. 2019;51:51–62. 30578418 10.1038/s41588-018-0303-9 PMC6365102

[10] Surendran P, Feofanova EV, Lahrouchi N, Ntalla I, Karthikeyan S, Cook J, et al. Discovery of rare variants associated with blood pressure regulation through meta-analysis of 1.3 million individuals. Nat Genet. 2020;52:1314–32. 33230300 10.1038/s41588-020-00713-x PMC7610439

[11] Said MA, Verweij N, van der Harst P. Associations of combined genetic and lifestyle risks with incident cardiovascular disease and diabetes in the UK Biobank Study. JAMA Cardiol. 2018;3:693–702. 29955826 10.1001/jamacardio.2018.1717 PMC6143077

[12] Vaura F, Kauko A, Suvila K, Havulinna AS, Mars N, Salomaa V, et al. Polygenic risk scores predict hypertension onset and cardiovascular risk. Hypertension. 2021;77:1119–27. 33611940 10.1161/HYPERTENSIONAHA.120.16471 PMC8025831

[13] Kurniansyah N, Goodman MO, Kelly TN, Elfassy T, Wiggins KL, Bis JC, et al. A multi-ethnic polygenic risk score is associated with hypertension prevalence and progression throughout adulthood. Nat Commun. 2022;13:3549. 35729114 10.1038/s41467-022-31080-2 PMC9213527

[14] Niu M, Zhang L, Wang Y, Tu R, Liu X, Wang C, et al. Lifestyle score and genetic factors with hypertension and blood pressure among adults in rural China. Front Public Health. 2021;9:687174. 34485217 10.3389/fpubh.2021.687174 PMC8416040

[15] Takase M, Hirata T, Nakaya N, Nakamura T, Kogure M, Hatanaka R, et al. Associations of combined genetic and lifestyle risks with hypertension and home hypertension. Hypertens Res. 2024;47:2064–74. 38914703 10.1038/s41440-024-01705-8 PMC11298407

[16] Fujii R, Hishida A, Nakatochi M, Tsuboi Y, Suzuki K, Kondo T, et al. Associations of genome-wide polygenic risk score and risk factors with hypertension in a Japanese population. Circ Genom Precis Med. 2022;15:e003612. 35666837 10.1161/CIRCGEN.121.003612

[17] Tsugane S. Why has Japan become the world’s most long-lived country: insights from a food and nutrition perspective. Eur J Clin Nutr. 2021;75:921–8. 32661353 10.1038/s41430-020-0677-5 PMC8189904

[18] Martin AR, Kanai M, Kamatani Y, Okada Y, Neale BM, Daly MJ. Clinical use of current polygenic risk scores may exacerbate health disparities. Nat Genet. 2019;51:584–91. 30926966 10.1038/s41588-019-0379-x PMC6563838

[19] Parikh NI, Pencina MJ, Wang TJ, Benjamin EJ, Lanier KJ, Levy D, et al. A risk score for predicting near-term incidence of hypertension: the Framingham Heart Study. Ann Intern Med. 2008;148:102–10. 18195335 10.7326/0003-4819-148-2-200801150-00005

[20] Niiranen TJ, McCabe EL, Larson MG, Henglin M, Lakdawala NK, Vasan RS, et al. Heritability and risks associated with early onset hypertension: multigenerational, prospective analysis in the Framingham Heart Study. BMJ. 2017;357:j1949. 28500036 10.1136/bmj.j1949 PMC5430541

[21] Niiranen TJ, McCabe EL, Larson MG, Henglin M, Lakdawala NK, Vasan RS, et al. Risk for hypertension crosses generations in the community: a multi-generational cohort study. Eur Heart J. 2017;38:2300–8. 28430902 10.1093/eurheartj/ehx134 PMC6075041

[22] Hozawa A, Tanno K, Nakaya N, Nakamura T, Tsuchiya N, Hirata T, et al. Study profile of the Tohoku Medical Megabank community-based cohort study. J Epidemiol. 2021;31:65–76. 31932529 10.2188/jea.JE20190271 PMC7738642

[23] Kuriyama S, Yaegashi N, Nagami F, Arai T, Kawaguchi Y, Osumi N, et al. The Tohoku Medical Megabank Project: design and mission. J Epidemiol. 2016;26:493–511. 27374138 10.2188/jea.JE20150268 PMC5008970

[24] Hozawa A, Nakaya K, Nakaya N, Nakamura T, Kogure M, Hatanaka R, et al. Progress report of the Tohoku Medical Megabank community-based cohort study: study profile of the repeated center-based survey during second period in Miyagi Prefecture. J Epidemiol. 2024;34:434–43. 38403692 10.2188/jea.JE20230241 PMC11330705

[25] Takase M, Nakaya N, Tanno K, Kogure M, Hatanaka R, Nakaya K, et al. Relationship between traditional risk factors for hypertension and systolic blood pressure in the Tohoku Medical Megabank community-based cohort study. Hypertens Res. 2024;47:1533–45. 38424250 10.1038/s41440-024-01582-1 PMC11150157

[26] Sakaue S, Kanai M, Tanigawa Y, Karjalainen J, Kurki M, Koshiba S, et al. A cross-population Atlas of genetic associations for 220 human phenotypes. Nature Genetics. 2021;53:1415–24. 34594039 10.1038/s41588-021-00931-x PMC12208603

[27] Polygenic Risk Score Task Force of the International Common Disease Alliance. Responsible use of polygenic risk scores in the clinic: potential benefits, risks and gaps. Nat Med. 2021;27:1876–84. 34782789 10.1038/s41591-021-01549-6

[28] Saadatagah S, Naderian M, Dikilitas O, Hamed ME, Bangash H, Kullo IJ. Polygenic risk, rare variants, and family history: independent and additive effects on coronary heart disease. JACC Adv. 2023;2:100567. 38939477 10.1016/j.jacadv.2023.100567 PMC11198423

[29] Hujoel MLA, Loh PR, Neale BM, Price AL. Incorporating family history of disease improves polygenic risk scores in diverse populations. Cell Genom. 2022;2:100152. 35935918 10.1016/j.xgen.2022.100152 PMC9351615

[30] Mars N, Lindbohm JV, Della Briotta Parolo P, Widén E, Kaprio J, Palotie A, et al. Systematic comparison of family history and polygenic risk across 24 common diseases. Am J Hum Genet. 2022;109:2152–62. 36347255 10.1016/j.ajhg.2022.10.009 PMC9748261

[31] Takase M, Nakaya N, Nakamura T, Kogure M, Hatanaka R, Nakaya K, et al. Influence of diabetes family history on the associations of combined genetic and lifestyle risks with diabetes in the Tohoku Medical Megabank community-based cohort study. J Atheroscler Thromb. 2023;30:1950–65. 37813642 10.5551/jat.64425 PMC10703570

[32] Takase M, Nakaya N, Nakamura T, Kogure M, Hatanaka R, Nakaya K, et al. Genetic risk, healthy lifestyle adherence, and risk of developing diabetes in the Japanese population. J Atheroscler Thromb. 2024;31:1717–32. 38910120 10.5551/jat.64906 PMC11620841

[33] Kato N, Loh M, Takeuchi F, Verweij N, Wang X, Zhang W, et al. Trans-ancestry genome-wide association study identifies 12 genetic loci influencing blood pressure and implicates a role for DNA methylation. Nat Genet. 2015;47:1282–93. 26390057 10.1038/ng.3405 PMC4719169

[34] Lin YT, Salihovic S, Fall T, Hammar U, Ingelsson E, Ärnlöv J, et al. Global plasma metabolomics to identify potential biomarkers of blood pressure progression. Arterioscler Thromb Vasc Biol. 2020;40:e227–237. 32460578 10.1161/ATVBAHA.120.314356

[35] Dietrich S, Floegel A, Weikert C, Prehn C, Adamski J, Pischon T, et al. Identification of Serum Metabolites Associated With Incident Hypertension in the European Prospective Investigation into Cancer and Nutrition-Potsdam Study. Hypertension. 2016;68:471–7. 10.1161/HYPERTENSIONAHA.116.07292 27245178

[36] Sun BB, Chiou J, Traylor M, Benner C, Hsu YH, Richardson TG, et al. Plasma proteomic associations with genetics and health in the UK Biobank. Nature. 2023;622:329–38. 37794186 10.1038/s41586-023-06592-6 PMC10567551

[37] Surendran P, Stewart ID, Au Yeung VPW, Pietzner M, Raffler J, Wörheide MA, et al. Rare and common genetic determinants of metabolic individuality and their effects on human health. Nat Med. 2022;28:2321–32. 36357675 10.1038/s41591-022-02046-0 PMC9671801

[38] Han X, Lains I, Li J, Li J, Chen Y, Yu B, et al. Integrating genetics and metabolomics from multi-ethnic and multi-fluid data reveals putative mechanisms for age-related macular degeneration. Cell Rep Med. 2023;4:101085. 37348500 10.1016/j.xcrm.2023.101085 PMC10394104

[39] Li J, Feng M, Wang Y, Li Y, Zhang Y, Li L, et al. The relationship between three X-linked genes and the risk for hypertension among northeastern Han Chinese. J Renin Angiotensin Aldosterone Syst. 2015;16:1321–28. 25143330 10.1177/1470320314534510

[40] Ohkubo T, Imai Y, Tsuji I, Nagai K, Kato J, Kikuchi N, et al. Home blood pressure measurement has a stronger predictive power for mortality than does screening blood pressure measurement: a population-based observation in Ohasama, Japan. J Hypertens. 1998;16:971–75. 9794737 10.1097/00004872-199816070-00010

[41] Hozawa A, Ohkubo T, Nagai K, Kikuya M, Matsubara M, Tsuji I, et al. Prognosis of isolated systolic and isolated diastolic hypertension as assessed by self-measurement of blood pressure at home: the Ohasama study. Arch Intern Med. 2000;160:3301–06. 11088093 10.1001/archinte.160.21.3301

[42] Hozawa A, Kuriyama S, Watanabe I, Kakizaki M, Ohmori-Matsuda K, Sone T, et al. Participation in health check-ups and mortality using propensity score matched cohort analyses. Prev Med. 2010;51:397–402. 20828583 10.1016/j.ypmed.2010.08.017

