# Supplementary material for: Associations of family history of hypertension, genetic, and lifestyle risks with incident hypertension
Source: Hypertens Res. 2025 Aug 13;48(10):2606–17. doi: 10.1038/s41440-025-02314-9 (PMC12497643; doi:10.1038/s41440-025-02314-9)
Supplement: Supplementary file 1 — Supplementary Online Content [file 41440_2025_2314_MOESM1_ESM.docx]

**Supplementary Online Content**

**Associations of family history of hypertension, genetic and lifestyle risks with risk of hypertension: the Tohoku Medical Megabank Community-Based Cohort Study**

**Author list**

Masato Takase^a,b^, Takumi Hirata^b,c^, Naoki Nakaya^a,b^, Mana Kogure^a,b^, Rieko Hatanaka^a,b^, Kumi Nakaya^a,b^, Ippei Chiba^a,b^, Sayuri Tokioka^a,b^, Kotaro Nochioka^a,b,d^, Tomohiro Nakamura^b,e^,Naho Tsuchiya^a,b^, Hirohito Metoki^b,f^, Michihiro Satoh^b,f^, Akira Narita^a,b^, Taku Obara^a,b^, Mami Ishikuro^a,b^, Hisashi Ohseto^a,b^, Ippei Takahashi ^a,b^, Tomoko Kobayashi^a,b,d^ Eiichi N Kodama^b,g^, Yohei Hamanaka^a,b^, Masatsugu Orui^a,b^, Soichi Ogishima^a,b^ Satoshi Nagaie^a,b^, Nobuo Fuse^a,b^, Junichi Sugawara^a,b,d,h^, Shinichi Kuriyama^a,b,g^, Gen Tamiya^a,b,i^, Atsushi Hozawa^a,b^_,_ Masayuki Yamamoto^a,b^_,_ and the ToMMo investigators

Supplement 1.

eMethods

**eMethods**

**Healthy lifestyle factors**

Five well-known modifiable risk factors were used and scored: obesity, smoking, drinking, regular physical activity, and low urinary Na/K ratio [1-4]. Height and weight data were collected during municipal health check-ups, with obesity defined as a body mass index (BMI) of ≥25.0 kg/m^2^, following the Western Pacific Region criteria of the World Health Organization for the Japanese population [5]. Data on smoking status and drinking status was collected by a self-reported questionnaire. Smoking status was classified into the following three categories: never-smokers (had smoked <100 cigarettes in their lifestyle), ex-smokers (had smoked ≥100 cigarettes in their lifetime and were not current smokers), current smokers (had smoked ≥100 cigarettes in their lifetime and were current smokers) [6]. Drinking status was classified into the following three categories: never drinkers (had consumed little or no alcohol or were constitutionally incapable of alcohol consumption), ex-drinkers (had stopped drinking alcohol), and current drinkers (were currently drinking).

Data on physical activity were gathered through a self-reported questionnaire, where participants indicated the hours spent on various activities (sitting, standing, walking, and strenuous work) per average day over the past years [7]. We assigned time values (0, 30, 120, 240, 360, 480, 600, and 660 min) corresponding to different activity levels (none, <1 h, 1–3 h, 3–5 h, 5–7 h, 7–9 h, 9–11 h, and ≥11 h, respectively). Information was collected on the frequency (times/week) and duration (min/time) of normal walking, brisk walking, moderate-intensity exercise, and hard-intensity exercise during leisure time [7-9]. Frequency was classified into the following categories: <once per month, 1–3 times per month, 1–2 times per week, 3–4 times per week, 6–8 times per week, and almost every day. Duration was classified into <0 min, 30–59 min, 1–2 h, 2–3 h, 3–4 h, and ≥4 h. This study classified "walking," "normal walking," "brisk walking," and "moderate-intensity exercise" as moderate physical activity, and "strenuous work" and "hard-intensity exercise" as vigorous physical activity [7-10]. The average time spent on moderate and vigorous physical activities during leisure time was calculated by multiplying the duration and frequency of these activities. Regular physical activity was defined as engaging in at least 150 min of moderate activity per week or 75 min of vigorous activity per week [11].

Casual spot urine samples were collected from each participant to estimate the 24-h urinary excretion of sodium and potassium, calculated using the Tanaka formula [12]. Subsequently, we calculated the Na/K by dividing 24-h sodium excretion by 24-h potassium excretion. A low Na/K ratio was defined as a ratio < 4.0 because the Japanese Society of Hypertension Working Group on Urine Sodium-to-Potassium Ratio suggested that an average urinary Na/k ratio of 4 is a feasible target value [13]. Overall lifestyle was categorized into ideal (having at least four ideal lifestyle factors), intermediate (having two or three ideal lifestyle factors), or poor (having less than two ideal lifestyle factors).

**Genotyping and quality control**

Study participants underwent genotyping using an Affymetrix Axiom Japonica Array (v2) in 21 separate batches [14,15]. The raw genotype data were pre-phased using SHAPEIT2 [16]; the phased genotypes were imputed using IMPUTE 4 [17], employing a cross-imputed haplotype reference panel comprising the 3.5KJPNv2 [18] and the 1000 Genomes phase 3 panel [19]. Cross-imputation of the two reference panels was performed using IMPUTE2 with the merge_ref_panels_output_ref option [20]. For quality control, we excluded plates with an average call rate < 0.95 and removed samples with a dish QC metric < 0.82 or step 1 call rate < 0.97 before batch genotyping. Subsequently, variants with a Hardy–Weinberg Equilibrium test P-value < 1.00 × 10-6, minor allele frequency (MAF) < 0.01, or missing rate > 0.01 were removed from each batch. The imputed genotype datasets for the 21 batches were merged using QCTOOL (v2.0.4) (https://www.well.ox.ac.uk~gavqctool), resulting in imputed and direct genotype datasets in Oxford BGEN and PLINK BED formats, respectively, from TMM CommCohort study participants. Quality control for the imputed genotype dataset removed variants with an MAF < 0.01 and IMPUTE2 info score < 0.8.

To compute genetic principal components (PCs) for population stratification adjustment, we first filtered variants based on genotype missingness (geno > 0.01), Hardy–Weinberg equilibrium (P < 0.05), and minor allele frequency (MAF < 0.05). We then applied linkage disequilibrium (LD) pruning using the --indep-pairwise 1500 150 0.03 option in PLINK to retain a set of approximately independent variants. To avoid bias due to relatedness, one individual from each pair with a genetic relatedness (PI_HAT > 0.1875) was excluded. PCA was performed on the remaining unrelated individuals, and the top 10 PCs were calculated. Subsequently, PCs for the excluded related individuals were computed by projecting their genotype data onto the eigenvectors obtained from the unrelated subset. This projection ensured that population structure was represented consistently across the entire cohort. The resulting PCs were included as covariates in regression models to adjust for underlying population structure.

**Reference**

1. Umemura S, Arima H, Arima S, Asayama K, Dohi Y, Hirooka Y, et al. The Japanese Society of Hypertension Guidelines for the Management of Hypertension (JSH 2019). Hypertens Res. 2019;42:1235–481.
2. Whelton PK, Carey RM, Aronow WS, Casey DE, Collins KJ, Dennison Himmelfarb C, et al. 2017 ACC/AHA/AAPA/ABC/ACPM/AGS/APhA/ASH/ASPC/NMA/PCNA Guideline for the Prevention, Detection, Evaluation, and Management of High Blood Pressure in Adults: A Report of the American College of Cardiology/American Heart Association Task Force on Clinical Practice Guidelines. Hypertension. 2018;71:e13–e115.
3. Williams B, Mancia G, Spiering W, Agabiti Rosei EA, Azizi M, Burnier M, et al. 2018 ESC/ESH Guidelines for the Management of Arterial Hypertension. Eur Heart J. 2018;39:3021–104.
4. US Preventive Services Task Force, Krist AH, Davidson KW, Mangione CM, Cabana M, Caughey AB, et al. Screening for hypertension in adults: US Preventive Services Task Force reaffirmation recommendation statement. JAMA. 2021;325:1650–6.
5. WHO/IASO/IOTF. The Asia-Pacific perspective: redefining obesity and its treatment. Health Commun Aust Pty Ltd: Australia, 2000.
6. Hamilton CM, Strader LC, Pratt JG, Maiese D, Hendershot T, Kwok RK, et al. The PhenX toolkit: get the most from your measures. Am J Epidemiol. 2011;174:253–60.
7. Fujii H, Yamamoto S, Takeda-Imai F, Inoue M, Tsugane S, Kadowaki T, et al. Validity and applicability of a simple questionnaire for the estimation of total and domain-specific physical activity. Diabetol Int 2011; 2: 47–54.
8. Kikuchi H, Inoue S, Lee IM, Odagiri Y, Sawada N, Inoue M, et al. Impact of moderate-intensity and vigorous-intensity physical activity on mortality. Med Sci Sports Exerc 2018; 50: 715–21.
9. Kikuchi H, Inoue S, Odagiri Y, Ihira H, Inoue M, Sawada N, et al. Intensity-specific validity and reliability of the Japan Public Health Center-based prospective study-physical activity questionnaire. Prev Med Rep 2020; 20: 101169.
10. MacIntosh BR, Murias JM, Keir DA, Weir JM. What is moderate to vigorous exercise intensity? Front Physiol 2021; 12: 682233.
11. WHO Guidelines on physical activity and sedentary behavior <https://iris.who.int/bitstream/handle/10665/336656/9789240015128-eng.pdf?sequence=1>
12. Tanaka T, Okamura T, Miura K, Kadowaki T, Ueshima H, Nakagawa H, et al. A simple method to estimate populational 24-h urinary sodium and potassium excretion using a casual urine specimen. J Hum Hypertens 2002; 16: 97–103.
13. Hisamatsu T, Kogure M, Tabara Y, et al. Practical use and target value of urine sodium-to-potassium ratio in assessment of hypertension risk for Japanese: Consensus Statement by the Japanese Society of Hypertension Working Group on Urine Sodium-to-Potassium Ratio. Hypertens Res. 2024 Oct 8.
14. Yamada M, Motoike IN, Kojima K, Fuse N, Hozawa A, Kuriyama S, et al. Genetic loci for lung function in Japanese adults with adjustment for exhaled nitric oxide levels as airway inflammation indicator. Commun Biol 2021; 4: 1288.
15. Fuse N, Sakurai M, Motoike IN, Kojima K, Takai-Igarashi T, Nakaya N, et al. Genome-wide association study of axial length in population-based cohorts in Japan: the Tohoku Medical Megabank Organization Eye Study. Ophthalmol Sci 2022; 2: 100113.
16. Delaneau O, Zagury JF, Marchini J. Improved whole-chromosome phasing for disease and population genetic studies. Nat Methods 2013; 10: 5–6.
17. Bycroft C, Freeman C, Petkova D, Band G, Elliott LT, Sharp K, et al. The UK biobank resource with deep phenotyping and genomic data. Nature 2018; 562: 203–9.
18. Tadaka S, Katsuoka F, Ueki M, Kojima K, Makino S, Saitō S, et al. 3.5KJPNv2: an allele frequency panel of 3552 Japanese individuals including the X chromosome. Hum Genome Var 2019; 6: 28.
19. 1000 Genomes Project Consortium, Auton A, Brooks LD, Durbin RM, Garrison EP, Kang HM et al. A global reference for human genetic variation. Nature 2015; 526: 68–74.
20. Howie BN, Donnelly P, Marchini J. A flexible and accurate genotype imputation method for the next generation of genome-wide association studies. PLOS Genet 2009; 5: e1000529.

| Supplemental Table 1. Characteristics of study participants according to the family history, genetic, and lifestyle risk | | | | | | | | | | | | | | | | | | | | | |
| --- | --- | --- | --- | --- | --- | --- | --- | --- | --- | --- | --- | --- | --- | --- | --- | --- | --- | --- | --- | --- | --- |
| Family history | No | | | | | | | | | Yes | | | | | | | | | | | |
| Genetic risk | Low | | | Intermediate | | | High | | | Low | | | Intermediate | | | | High | | |  | |
| Lifestyle score | Ideal | Intermediate | Poor | Ideal | Intermediate | Poor | Ideal | Intermediate | Poor | Ideal | Intermediate | Poor | Ideal | Intermediate | Poor | Ideal | | Intermediate | Poor | | Overall |
| Number | 849 | 1034 | 155 | 728 | 1104 | 188 | 585 | 1091 | 199 | 403 | 499 | 61 | 338 | 545 | 97 | 394 | | 620 | 111 | | 9001 |
| Age, years | 60.8 (10.6) | 59.4 (11.7) | 58.6 (11.9) | 59.9 (11.2) | 58.0 (12.3) | 57.2 (12.7) | 59.0 (11.1) | 57.1 (12.7) | 57.0 (12.8) | 58.7 (10.4) | 56.0 (11.9) | 55.1 (11.2) | 58.3 (11.1) | 55.4 (11.6) | 54.7 (13.0) | 57.6 (10.6) | | 54.5 (11.7) | 53.0 (10.9) | | 57.9 (11.8) |
| Women, % | 707 (83.3) | 563 (54.4) | 39 (25.2) | 645 (88.6) | 666 (60.3) | 66 (35.1) | 527 (90.1) | 683 (62.6) | 60 (30.2) | 365 (90.6) | 331 (66.3) | 20 (32.8) | 314 (92.9) | 406 (74.5) | 40 (41.2) | 362 (91.9) | | 463 (74.7) | 61 (55.0) | | 6318 (70.2) |
| SBP, mmHg | 116.0 (12.9) | 117.1 (12.5) | 121.1 (12.0) | 116.3 (12.8) | 117.6 (12.6) | 119.5 (12.0) | 117.0 (12.7) | 118.3 (12.4) | 121.8 (10.3) | 118.2 (12.4) | 118.3 (12.6) | 120.5 (11.1) | 116.7 (13.3) | 117.9 (12.8) | 120.7 (12.1) | 118.2 (12.4) | | 118.6 (12.3) | 120.2 (11.0) | | 117.8 (12.6) |
| DBP, mmHg | 69.7 (8.7) | 71.4 (8.8) | 75.4 (8.6) | 69.7 (8.7) | 71.4 (8.9) | 73.6 (8.4) | 70.3 (8.7) | 71.7 (8.9) | 75.2 (7.9) | 70.9 (8.6) | 71.9 (8.9) | 74.4 (8.8) | 70.7 (8.5) | 72.2 (8.8) | 73.9 (9.2) | 71.3 (8.4) | | 72.2 (8.8) | 73.4 (8.9) | | 71.4 (8.8) |
| Smoking Status, % |  |  |  |  |  |  |  |  |  |  |  |  |  |  |  |  | |  |  | |  |
| Never smoker | 761 (89.6) | 496 (48.0) | 7 (4.5) | 698 (95.9) | 618 (56.0) | 20 (10.6) | 572 (97.8) | 634 (58.1) | 22 (11.1) | 375 (93.1) | 268 (53.7) | 5  (8.2) | 321 (95.0) | 341 (62.6) | 14 (14.4) | 380 (96.4) | | 387 (62.4) | 15 (13.5) | | 5934 (65.9) |
| Ex-smoker | 52 (6.1) | 354 (34.2) | 99 (63.9) | 21 ( 2.9) | 315 (28.5) | 123 (65.4) | 10  (1.7) | 294 (26.9) | 121 (60.8) | 18 (4.5) | 150 (30.1) | 33 (54.1) | 10 (3.0) | 151 (27.7) | 53 ( 54.6) | 3 (0.8) | | 143 (23.1) | 57 (51.4) | | 2007 (22.3) |
| Current smoker | 36 (4.2) | 184  (17.8) | 49  (31.6) | 9  (1.2) | 171  (15.5) | 45  (23.9) | 3  (0.5) | 163  (14.9) | 56  (28.1) | 10  (2.5) | 81  (16.2) | 23  (37.7) | 7  (2.1) | 53  (9.7) | 30  (30.9) | 11  (2.8) | | 90  (14.5) | 39  (35.1) | | 1060  (11.8) |
| Drinking Status, % |  |  |  |  |  |  |  |  |  |  |  |  |  |  |  |  | |  |  | |  |
| Never drinker | 742 (87.4) | 460 (44.5) | 17 (11.0) | 565 (77.6) | 322 (29.2) | 9  (4.8) | 431 (73.7) | 236 (21.6) | 5  (2.5) | 350 (86.8) | 251 (50.3) | 5  (8.2) | 277 (82.0) | 177 (32.5) | 4  (4.1) | 299 (75.9) | | 140 (22.6) | 3  (2.7) | | 4293 (47.7) |
| Ex-drinker | 3 (0.4) | 35  (3.4) | 7 (4.5) | 1 (0.1) | 23 (2.1) | 8  (4.3) | 5  (0.9) | 36 (3.3) | 13 (6.5) | 1  (0.2) | 13 (2.6) | 4  (6.6) | 2 (0.6) | 18  (3.3) | 2  (2.1) | 3  (0.8) | | 26 (4.2) | 7  (6.3) | | 207 (2.3) |
| Current drinker | 104 (12.2) | 539 (52.1) | 131 (84.5) | 162 (22.3) | 759 (68.8) | 171 (91.0) | 149 (25.5) | 819 (75.1) | 181 (91.0) | 52 (12.9) | 235 (47.1) | 52 (85.2) | 59 (17.5) | 350 (64.2) | 91 (93.8) | 92 (23.4) | | 454 (73.2) | 101 (91.0) | | 4501 (50.0) |
| Moderate intensity physical activity, min/week | 180.0 [132.6, 264.4] | 131.7 [52.5, 205.7] | 97.5 [37.0, 139.3] | 171.4 [123.2, 262.8] | 130.7 [52.5, 211.0] | 111.4 [37.5, 137.3] | 177.6 [130.5, 268.9] | 139.3 [51.9, 210.1] | 97.5 [33.2, 139.6] | 178.9 [120.0, 258.7] | 123.0 [46.0, 201.5] | 55.3 [39.0, 120.0] | 171.8 [126.0, 247.6] | 124.0 [49.3, 176.4] | 75.0 [36.0, 138.0] | 175.3 [123.0, 262.5] | | 126.0 [49.6, 174.6] | 45.6 [31.0, 120.0] | | 142.5 [62.1, 229.3] |
| Vigorous intensity physical activity, min/week | 54.5 [30.0, 120.0] | 30.0 [19.3, 120.0] | 30.0 [12.6, 50.0] | 50.8 [30.0, 120.0] | 30.0 [9.6, 120.0] | 30.0 [0.0, 39.8] | 49.3 [30.0, 120.0] | 30.0 [19.3, 120.0] | 30.0 [0.0, 49.1] | 45.0 [30.0, 120.0] | 30.0 [0.0, 120.0] | 30.0 [0.0, 30.0] | 37.8 [30.0, 120.0] | 30.0 [1.0, 75.0] | 30.0 [0.0, 33.0] | 30.0 [22.5, 120.0] | | 30.0 [0.0, 90.8] | 30.0 [0.0, 30.0] | | 30.0 [19.3, 120.0] |
| BMI, kg/m^2^ | 21.7 (2.6) | 22.8 (3.2) | 25.5 (3.2) | 21.4 (2.6) | 22.7 (3.2) | 25.1 (3.4) | 21.6 (2.4) | 22.7 (3.2) | 25.5 (3.3) | 21.4 (2.4) | 22.7 (3.3) | 24.4 (3.4) | 21.5 (2.4) | 22.7 (3.4) | 24.8 (3.4) | 21.5 (2.6) | | 22.2 (3.1) | 25.0 (3.7) | | 22.5 (3.2) |
| Na/K ratio | 3.7 (0.9) | 4.3 (1.0) | 4.7  (0.9) | 3.6  (0.8) | 4.2 (1.0) | 4.7  (0.9) | 3.6  (0.8) | 4.2  (1.0) | 4.7  (0.9) | 3.7  (0.9) | 4.2  (0.9) | 4.7  (0.8) | 3.6  (0.9) | 4.2  (1.0) | 4.7  (0.7) | 3.6  (0.8) | | 4.1  (1.0) | 4.7  (0.8) | | 4.0 (1.0) |
| Estimated sodium intake, g/day | 9.0 (1.9) | 9.6 (2.0) | 10.3 (2.1) | 8.9 (2.1) | 9.6 (2.2) | 10.2 (2.1) | 8.8 (1.9) | 9.5 (2.1) | 10.4 (2.1) | 9.0 (2.0) | 9.4 (2.0) | 10.5 (1.9) | 8.8 (2.0) | 9.5 (2.1) | 10.5 (2.1) | 8.9 (1.9) | | 9.3 (2.0) | 10.2 (1.9) | | 9.4 (2.1) |
| Education levels, % |  |  |  |  |  |  |  |  |  |  |  |  |  |  |  |  | |  |  | |  |
| Below high school | 557 (65.6) | 661 (63.9) | 104 (67.1) | 474 (65.1) | 725 (65.7) | 127 (67.6) | 361 (61.7) | 717 (65.7) | 144 (72.4) | 222 (55.1) | 265 (53.1) | 34 (55.7) | 190 (56.2) | 320 (58.7) | 55 (56.7) | 236 (59.9) | | 351 (56.6) | 67 (60.4) | | 5610 (62.3) |
| Vocational school or junior college or technical college | 218 (25.7) | 228 (22.1) | 23 (14.8) | 204 (28.0) | 242 (21.9) | 36 (19.1) | 173 (29.6) | 252 (23.1) | 33 (16.6) | 119 (29.5) | 142 (28.5) | 14 (23.0) | 114 (33.7) | 147 (27.0) | 22 (22.7) | 125 (31.7) | | 180 (29.0) | 31 (27.9) | | 2303 (25.6) |
| University or graduate school | 62 (7.3) | 142 (13.7) | 28 (18.1) | 44 (6.0) | 133 (12.0) | 25 (13.3) | 45  (7.7) | 110 (10.1) | 21 (10.6) | 57 (14.1) | 88 (17.6) | 13 (21.3) | 31 (9.2) | 76 (13.9) | 18 (18.6) | 32 (8.1) | | 85 (13.7) | 12 (10.8) | | 1022 (11.4) |
| Other | 12 (1.4) | 3  (0.3) | 0 (0.0) | 6 (0.8) | 4  (0.4) | 0  (0.0) | 6  (1.0) | 12 (1.1) | 1  (0.5) | 5  (1.2) | 4  (0.8) | 0  (0.0) | 3 (0.9) | 2  (0.4) | 2  (2.1) | 1  (0.3) | | 4  (0.6) | 1  (0.9) | | 66 (0.7) |
| Obesity, % | 48 (5.7) | 223 (21.6) | 102 (65.8) | 33 (4.5) | 214 (19.4) | 121 (64.4) | 26  (4.4) | 210 (19.2) | 131 (65.8) | 16 (4.0) | 119 (23.8) | 31 (50.8) | 12 (3.6) | 118 (21.7) | 58 (59.8) | 22 (5.6) | | 106 (17.1) | 60 (54.1) | | 1650 (18.3) |
| High Na/K ratio | 233 (27.4) | 628 (60.7) | 137 (88.4) | 169 (23.2) | 634 (57.4) | 163 (86.7) | 121 (20.7) | 619 (56.7) | 172 (86.4) | 99 (24.6) | 312 (62.5) | 55 (90.2) | 83 (24.6) | 315 (57.8) | 87 (89.7) | 79 (20.1) | | 338 (54.5) | 99 (89.2) | | 4343 (48.3) |
| Regular physical activity, % | 151 (17.8) | 509 (49.2) | 117 (75.5) | 146 (20.1) | 540 (48.9) | 143 (76.1) | 115 (19.7) | 526 (48.2) | 146 (73.4) | 88 (21.8) | 262 (52.5) | 53 (186.9) | 73 (21.6) | 312 (57.2) | 78 (80.4) | 95 (24.1) | | 348 (6.1) | 95 (85.6) | | 3797 (42.2) |

BMI, body mass index; DBP, diastolic blood pressure; Na/K ratio, sodium-to-potassium ratio, SBP, systolic blood pressure.

Obesity was defined as BMI ≥25.0 kg/m^2^ based on the Western Pacific Region of World Health Organization criteria in Japanese individuals.

The high Na/K ratio was defined as ≥ 4.0.

Insufficient regular physical activity was defined as not meeting the American Heart Association recommendations of at least 150 min of moderate activity per week or 75min of vigorous activity per week.

| Supplemental Table 2. Characteristics of participants with and without follow-up | | |
| --- | --- | --- |
|  | Not included | Included |
| Number | 10580 | 9001 |
| Age, years | 55.5 (13.4) | 57.9 (11.8) |
| Women, % | 6678 (63.1) | 6318 (70.2) |
| SBP, mmHg | 118.7 (12.3) | 117.8 (12.6) |
| DBP, mmHg | 72.1 (8.8) | 71.4 (8.8) |
| Smoking Status, % |  |  |
| Never smoker | 6249 (59.1) | 5934 (65.9) |
| Ex-smoker | 2281 (21.6) | 2007 (22.3) |
| Current smoker | 2050 (19.4) | 1060 (11.8) |
| Drinking Status, % |  |  |
| Never drinker | 5071 (47.9) | 4293 (47.7) |
| Ex-drinker | 268 (2.5) | 207 (3.3) |
| Current drinker | 5241 (49.5) | 4501 (50.0) |
| Moderate intensity physical activity, min/week | 153.5 [78.0, 240.0] | 142.5 [62.1, 229.3] |
| Vigorous intensity physical activity, min/week | 49.3 [30.0, 120.0] | 30.0 [19.3, 120.0] |
| BMI, kg/m^2^ | 22.8 (3.5) | 22.5 (3.2) |
| Na/K ratio | 4.1 (1.0) | 4.0 (1.0) |
| Education levels, % |  |  |
| Below high school | 7345 (69.4) | 5610 (62.3) |
| Vocational school or junior college or technical college | 2310 (21.8) | 2303 (25.6) |
| University or graduate school | 874 (8.3) | 1022 (11.4) |
| Other | 51 (0.5) | 66 (0.7) |
| Obesity, % | 2408 (22.8) | 1650 (18.3) |
| High Na/K ratio | 5456 (51.6) | 4343 (48.3) |
| Regular physical activity, % | 3866 (36.5) | 3797 (42.2) |

BMI, body mass index; DBP, diastolic blood pressure; Na/K ratio, sodium-to-potassium ratio, SBP, systolic blood pressure.

Obesity was defined as BMI ≥25.0 kg/m^2^ based on the Western Pacific Region of World Health Organization criteria in Japanese individuals.

The high Na/K ratio was defined as ≥ 4.0.

Insufficient regular physical activity was defined as not meeting the American Heart Association recommendations of at least 150 min of moderate activity per week or 75min of vigorous activity per week.

| Supplemental Table 2. Adjusted LS means and 95% CI of SBP in family history, genetic risk and lifestyle risk. | | | | |
| --- | --- | --- | --- | --- |
| Family history | Genetic risk | Lifestyle score | LS means, 95% CI | |
| No | Low | Ideal (≤1 poor factors) | 127 | (126-129) |
|  |  | Intermediate (2-3 poor factors) | 127 | (126-129) |
|  |  | Poor (≥4 poor factors) | 129 | (127-132) |
|  | Intermediate | Ideal (≤1 poor factors) | 129 | (127-130) |
|  |  | Intermediate (2-3 poor factors) | 128 | (127-130) |
|  |  | Poor (≥4 poor factors) | 130 | (127-132) |
|  | High | Ideal (≤1 poor factors) | 130 | (129-132) |
|  |  | Intermediate (2-3 poor factors) | 130 | (129-131) |
|  |  | Poor (≥4 poor factors) | 133 | (131-135) |
| Yes | Low | Ideal (≤1 poor factors) | 130 | (128-132) |
|  |  | Intermediate (2-3 poor factors) | 129 | (127-131) |
|  |  | Poor (≥4 poor factors) | 130 | (126-134) |
|  | Intermediate | Ideal (≤1 poor factors) | 129 | (127-131) |
|  |  | Intermediate (2-3 poor factors) | 131 | (129-133) |
|  |  | Poor (≥4 poor factors) | 131 | (128-135) |
|  | High | Ideal (≤1 poor factors) | 132 | (120-134) |
|  |  | Intermediate (2-3 poor factors) | 132 | (131-134) |
|  |  | Poor (≥4 poor factors) | 134 | (131-137) |

CI, confidence interval; LS means, least square means; SBP, systolic blood pressure

The model was adjusted for age, sex, education status, and the first ten principal components.

Lifestyle is categorized as ideal (having at least three ideal lifestyle factors), poor (having 0–1 ideal lifestyle factors), intermediate (having 2–3 ideal lifestyle factors).

Lifestyle score included the following factors: Obesity, defined as BMI ≥25.0 kg/m^2^ based on the Western Pacific Region of World Health Organization criteria in Japanese individuals, high Na/K ratio, defined as ≥ 4.0, insufficient regular physical activity, defined as not meeting the American Heart Association recommendations of at least 150 min of moderate activity per week or 75min of vigorous activity per week, smoking, defined as ex-smoker or current smoker, drinking, defined as ex-drinker or current drinker.

Genetic risk was classified based on the tertile of polygenic risk score.

| Supplemental Table 3. Association between genetic and lifestyle risk with hypertension incidence | | | | | |
| --- | --- | --- | --- | --- | --- |
| Family history | Lifestyle score | Hypertension/number of participants | % | RR, 95% CI | |
| No | Ideal (<1 poor factors) | 613/2162 | (28.4) | Ref | |
|  | Intermediate (2-3 poor factors) | 982/3229 | (30.4) | 1.08 | (0.99-1.18) |
|  | Poor (≥4 poor factors) | 213/542 | (39.3) | 1.37 | (1.20-1.56) |
| Yes | Ideal (<1 poor factors) | 367/1135 | (32.3) | 1.24 | (1.11-1.38) |
|  | Intermediate (2-3 poor factors) | 548/1664 | (32.9) | 1.35 | (1.23-1.49) |
|  | Poor (≥4 poor factors) | 99/269 | (36.8) | 1.50 | (1.26-1.78) |

The model was adjusted for age, sex, education status, and the first ten principal components.

Hypertension was defined as systolic/diastolic blood pressure of 140/90mmHg or higher measured at a community-support center and/or self-reported treatment for hypertension.

Lifestyle is categorized as ideal (having at least three ideal lifestyle factors), poor (having 0–1 ideal lifestyle factors), intermediate (having 2–3 ideal lifestyle factors).

Lifestyle score included the following factors: Obesity, defined as BMI ≥25.0 kg/m^2^ based on the Western Pacific Region of World Health Organization criteria in Japanese individuals, high Na/K ratio, defined as ≥ 4.0, insufficient regular physical activity, defined as not meeting the American Heart Association recommendations of at least 150 min of moderate activity per week or 75min of vigorous activity per week, smoking, defined as ex-smoker or current smoker, drinking, defined as ex-drinker or current drinker.

| Supplemental Table S4. Association between genetic and lifestyle risk with hypertension incidence in participants aged under 60 years old | | | | | | |
| --- | --- | --- | --- | --- | --- | --- |
| Family history | Genetic risk | Lifestyle score | case /number of participants | % | RR, 95% CI | |
| No | Low | Ideal (≤1 poor factors) | 49/321 | (15.3) | Ref | |
|  |  | Intermediate (2-3 poor factors) | 77/450 | (17.1) | 1.26 | (0.82-1.95) |
|  |  | Poor (≥4 poor factors) | 14/67 | (20.9) | 1.62 | (0.92-2.86) |
|  | Intermediate | Ideal (≤1 poor factors) | 51/302 | (16.9) | 1.26 | (0.78-2.03) |
|  |  | Intermediate (2-3 poor factors) | 92/512 | (18.0) | 1.61 | (1.07-2.43) |
|  |  | Poor (≥4 poor factors) | 21/90 | (23.3) | 1.95 | (1.14-3.36) |
|  | High | Ideal (≤1 poor factors) | 49/262 | (18.7) | 1.26 | (0.78-2.03) |
|  |  | Intermediate (2-3 poor factors) | 117/543 | (21.5) | 1.80 | (1.20-2.69) |
|  |  | Poor (≥4 poor factors) | 35/100 | (35.0) | 2.62 | (1.62-4.23) |
| Yes | Low | Ideal (≤1 poor factors) | 47/199 | (23.6) | 1.69 | (1.05-2.71) |
|  |  | Intermediate (2-3 poor factors) | 60/282 | (21.3) | 1.74 | (1.12-2.69) |
|  |  | Poor (≥4 poor factors) | 10/40 | (25.0) | 2.20 | (1.22-3.96) |
|  | Intermediate | Ideal (≤1 poor factors) | 34/169 | (20.1) | 1.29 | (0.76-2.20) |
|  |  | Intermediate (2-3 poor factors) | 89/325 | (27.4) | 2.13 | (1.42-3.21) |
|  |  | Poor (≥4 poor factors) | 11/56 | (19.6) | 1.47 | (0.69-3.16) |
|  | High | Ideal (≤1 poor factors) | 65/226 | (28.8) | 2.31 | (1.50-3.54) |
|  |  | Intermediate (2-3 poor factors) | 120/400 | (30.0) | 2.47 | (1.67-3.66) |
|  |  | Poor (≥4 poor factors) | 30/80 | (37.5) | 2.97 | (1.88-4.70) |

CI, confidence interval; LS means, least square means; SBP, systolic blood pressure

The model was adjusted for age, sex, education status, and the first ten principal components.

Hypertension was defined as systolic/diastolic blood pressure of 140/90mmHg or higher measured at a community-support center and/or self-reported treatment for hypertension.

Lifestyle is categorized as ideal (having at least three ideal lifestyle factors), poor (having 0–1 ideal lifestyle factors), intermediate (having 2–3 ideal lifestyle factors).

Lifestyle score included the following factors: Obesity, defined as BMI ≥25.0 kg/m^2^ based on the Western Pacific Region of World Health Organization criteria in Japanese individuals, high Na/K ratio, defined as ≥ 4.0, insufficient regular physical activity, defined as not meeting the American Heart Association recommendations of at least 150 min of moderate activity per week or 75min of vigorous activity per week, smoking, defined as ex-smoker or current smoker, drinking, defined as ex-drinker or current drinker.

| Supplemental Table 5. Association between genetic and lifestyle risk with hypertension incidence in participants aged over 60 years | | | | | | |
| --- | --- | --- | --- | --- | --- | --- |
| Family history | Genetic risk | Lifestyle score | case /number of participants | % | RR, 95% CI | |
| No | Low | Ideal (≤1 poor factors) | 186/528 | (35.2) | Ref | |
|  |  | Intermediate (2-3 poor factors) | 242/584 | (41.4) | 1.09 | (0.94-1.26) |
|  |  | Poor (≥4 poor factors) | 38/88 | (43.2) | 1.10 | (0.84-1.44) |
|  | Intermediate | Ideal (≤1 poor factors) | 148/426 | (34.7) | 1.02 | (0.87-1.20) |
|  |  | Intermediate (2-3 poor factors) | 220/592 | (37.2) | 1.00 | (0.86-1.16) |
|  |  | Poor (≥4 poor factors) | 50/98 | (51.0) | 1.34 | (1.07-1.69) |
|  | High | Ideal (≤1 poor factors) | 130/323 | (40.2) | 1.20 | (1.02-1.42) |
|  |  | Intermediate (2-3 poor factors) | 234/548 | (42.7) | 1.21 | (1.04-1.40) |
|  |  | Poor (≥4 poor factors) | 55/99 | (55.6) | 1.52 | (1.24-1.86) |
| Yes | Low | Ideal (≤1 poor factors) | 85/204 | (41.7) | 1.26 | (1.05-1.52) |
|  |  | Intermediate (2-3 poor factors) | 76/217 | (35.0) | 1.01 | (0.83-1.24) |
|  |  | Poor (≥4 poor factors) | 7/21 | (33.3) | 0.81 | (0.43-1.51) |
|  | Intermediate | Ideal (≤1 poor factors) | 70/169 | (41.4) | 1.29 | (1.06-1.57) |
|  |  | Intermediate (2-3 poor factors) | 99/220 | (45.0) | 1.37 | (1.15-1.63) |
|  |  | Poor (≥4 poor factors) | 23/41 | (56.1) | 1.50 | (1.12-2.02) |
|  | High | Ideal (≤1 poor factors) | 66/168 | (39.3) | 1.14 | (0.93-1.41) |
|  |  | Intermediate (2-3 poor factors) | 104/220 | (47.3) | 1.40 | (1.18-1.65) |
|  |  | Poor (≥4 poor factors) | 18/31 | (58.1) | 1.69 | (1.27-2.25) |

CI, confidence interval; LS means, least square means; SBP, systolic blood pressure

The model was adjusted for age, sex, and the first ten principal components.

Hypertension was defined as systolic/diastolic blood pressure of 140/90mmHg or higher measured at a community-support center and/or self-reported treatment for hypertension.

Lifestyle is categorized as ideal (having at least three ideal lifestyle factors), poor (having 0–1 ideal lifestyle factors), intermediate (having 2–3 ideal lifestyle factors).

Lifestyle score included the following factors: Obesity, defined as BMI ≥25.0 kg/m^2^ based on the Western Pacific Region of World Health Organization criteria in Japanese individuals, high Na/K ratio, defined as ≥ 4.0, insufficient regular physical activity, defined as not meeting the American Heart Association recommendations of at least 150 min of moderate activity per week or 75min of vigorous activity per week, smoking, defined as ex-smoker or current smoker, drinking, defined as ex-drinker or current drinker.

| Supplemental Table 6. Association between genetic and lifestyle risk with hypertension incidence in participants Using PRS Constructed with a P-value Threshold < 5e-8 | | | | | | |
| --- | --- | --- | --- | --- | --- | --- |
| Family history | Genetic risk | Lifestyle score | case /number of participants | % | RR, 95% CI | |
| No | Low | Ideal (≤1 poor factors) | 228/871 | (26.2) | Ref | |
|  |  | Intermediate (2-3 poor factors) | 209/647 | (32.3) | 1.22 | (1.04-1.43) |
|  |  | Poor (≥4 poor factors) | 47/140 | (33.6) | 1.29 | (1.00-1.65) |
|  | Intermediate | Ideal (≤1 poor factors) | 182/667 | (27.3) | 1.07 | (0.91-1.26) |
|  |  | Intermediate (2-3 poor factors) | 178/625 | (28.5) | 1.14 | (0.97-1.35) |
|  |  | Poor (≥4 poor factors) | 83/196 | (42.3) | 1.57 | (1.28-1.93) |
|  | High | Ideal (≤1 poor factors) | 203/624 | (32.5) | 1.28 | (1.10-1.50) |
|  |  | Intermediate (2-3 poor factors) | 179/620 | (28.9) | 1.13 | (0.96-1.33) |
|  |  | Poor (≥4 poor factors) | 83/206 | (40.3) | 1.65 | (1.35-2.03) |
| Yes | Low | Ideal (≤1 poor factors) | 140/437 | (32.0) | 1.35 | (1.14-1.61) |
|  |  | Intermediate (2-3 poor factors) | 96/314 | (30.6) | 1.37 | (1.12-1.67) |
|  |  | Poor (≥4 poor factors) | 21/53 | (39.6) | 1.57 | (1.12-2.21) |
|  | Intermediate | Ideal (≤1 poor factors) | 119/358 | (33.2) | 1.39 | (1.16-1.67) |
|  |  | Intermediate (2-3 poor factors) | 114/321 | (35.5) | 1.60 | (1.33-1.91) |
|  |  | Poor (≥4 poor factors) | 32/107 | (29.9) | 1.30 | (0.95-1.78) |
|  | High | Ideal (≤1 poor factors) | 108/340 | (31.8) | 1.40 | (1.16-1.69) |
|  |  | Intermediate (2-3 poor factors) | 98/363 | (27.0) | 1.32 | (1.08-1.61) |
|  |  | Poor (≥4 poor factors) | 46/109 | (42.2) | 2.23 | (1.76-2.83) |

CI, confidence interval; LS means, least square means; SBP, systolic blood pressure

The model was adjusted for age, sex, and the first ten principal components.

Hypertension was defined as systolic/diastolic blood pressure of 140/90mmHg or higher measured at a community-support center and/or self-reported treatment for hypertension.

Lifestyle is categorized as ideal (having at least three ideal lifestyle factors), poor (having 0–1 ideal lifestyle factors), intermediate (having 2–3 ideal lifestyle factors).

Lifestyle score included the following factors: Obesity, defined as BMI ≥25.0 kg/m^2^ based on the Western Pacific Region of World Health Organization criteria in Japanese individuals, high Na/K ratio, defined as ≥ 4.0, insufficient regular physical activity, defined as not meeting the American Heart Association recommendations of at least 150 min of moderate activity per week or 75min of vigorous activity per week, smoking, defined as ex-smoker or current smoker, drinking, defined as ex-drinker or current drinker.

| Supplemental Table 7. Association between genetic and lifestyle risk with hypertension incidence in participants Using PRS Constructed with a P-value Threshold < 0.01 | | | | | | |
| --- | --- | --- | --- | --- | --- | --- |
| Family history | Genetic risk | Lifestyle score | case /number of participants | % | RR, 95% CI | |
| No | Low | Ideal (≤1 poor factors) | 217/838 | (25.9) | Ref | |
|  |  | Intermediate (2-3 poor factors) | 210/634 | (33.1) | 1.22 | (1.05-1.43) |
|  |  | Poor (≥4 poor factors) | 56/170 | (32.9) | 1.26 | (0.99-1.61) |
|  | Intermediate | Ideal (≤1 poor factors) | 203/708 | (28.7) | 1.14 | (0.97-1.34) |
|  |  | Intermediate (2-3 poor factors) | 181/648 | (27.9) | 1.19 | (1.01-1.40) |
|  |  | Poor (≥4 poor factors) | 63/174 | (36.2) | 1.39 | (1.11-1.74) |
|  | High | Ideal (≤1 poor factors) | 193/616 | (31.3) | 1.28 | (1.09-1.50) |
|  |  | Intermediate (2-3 poor factors) | 175/610 | (28.7) | 1.15 | (0.97-1.35) |
|  |  | Poor (≥4 poor factors) | 94/198 | (47.5) | 1.95 | (1.61-2.37) |
| Yes | Low | Ideal (≤1 poor factors) | 113/371 | (30.5) | 1.30 | (1.08-1.57) |
|  |  | Intermediate (2-3 poor factors) | 71/304 | (23.4) | 1.03 | (0.82-1.30) |
|  |  | Poor (≥4 poor factors) | 17/66 | (25.8) | 1.16 | (0.78-1.74) |
|  | Intermediate | Ideal (≤1 poor factors) | 104/344 | (30.2) | 1.29 | (1.07-1.57) |
|  |  | Intermediate (2-3 poor factors) | 110/354 | (31.1) | 1.50 | (1.24-1.81) |
|  |  | Poor (≥4 poor factors) | 36/88 | (40.9) | 1.69 | (1.28-2.23) |
|  | High | Ideal (≤1 poor factors) | 150/420 | (35.7) | 1.60 | (1.34-1.90) |
|  |  | Intermediate (2-3 poor factors) | 127/340 | (37.4) | 1.82 | (1.53-2.17) |
|  |  | Poor (≥4 poor factors) | 46/115 | (40.0) | 2.18 | (1.70-2.79) |

CI, confidence interval; LS means, least square means; SBP, systolic blood pressure

The model was adjusted for age, sex, and the first ten principal components.

Hypertension was defined as systolic/diastolic blood pressure of 140/90mmHg or higher measured at a community-support center and/or self-reported treatment for hypertension.

Lifestyle is categorized as ideal (having at least three ideal lifestyle factors), poor (having 0–1 ideal lifestyle factors), intermediate (having 2–3 ideal lifestyle factors).

Lifestyle score included the following factors: Obesity, defined as BMI ≥25.0 kg/m^2^ based on the Western Pacific Region of World Health Organization criteria in Japanese individuals, high Na/K ratio, defined as ≥ 4.0, insufficient regular physical activity, defined as not meeting the American Heart Association recommendations of at least 150 min of moderate activity per week or 75min of vigorous activity per week, smoking, defined as ex-smoker or current smoker, drinking, defined as ex-drinker or current drinker.

| Supplemental Table 8. Association between genetic and lifestyle risk with hypertension incidence in participants Using PRS Constructed with a P-value Threshold < 0.05 | | | | | | |
| --- | --- | --- | --- | --- | --- | --- |
| Family history | Genetic risk | Lifestyle score | case /number of participants | % | RR, 95% CI | |
| No | Low | Ideal (≤1 poor factors) | 210/787 | (26.7) | Ref | |
|  |  | Intermediate (2-3 poor factors) | 206/653 | (31.5) | 1.15 | (0.99-1.35) |
|  |  | Poor (≥4 poor factors) | 60/177 | (33.9) | 1.25 | (0.98-1.59) |
|  | Intermediate | Ideal (≤1 poor factors) | 222/720 | (30.8) | 1.20 | (1.03-1.41) |
|  |  | Intermediate (2-3 poor factors) | 182/632 | (28.8) | 1.12 | (0.95-1.32) |
|  |  | Poor (≥4 poor factors) | 66/181 | (36.5) | 1.43 | (1.14-1.79) |
|  | High | Ideal (≤1 poor factors) | 181/655 | (27.6) | 1.10 | (0.94-1.30) |
|  |  | Intermediate (2-3 poor factors) | 178/607 | (29.3) | 1.211 | (1.02-1.42) |
|  |  | Poor (≥4 poor factors) | 87/184 | (47.3) | 1.86 | (1.52-2.26) |
| Yes | Low | Ideal (≤1 poor factors) | 111/360 | (30.8) | 1.26 | (1.05-1.51) |
|  |  | Intermediate (2-3 poor factors) | 74/309 | (23.9) | 1.06 | (0.85-1.33) |
|  |  | Poor (≥4 poor factors) | 23/72 | (31.9) | 1.35 | (0.96-1.91) |
|  | Intermediate | Ideal (≤1 poor factors) | 101/348 | (29.0) | 1.24 | (1.01-1.51) |
|  |  | Intermediate (2-3 poor factors) | 104/341 | (30.5) | 1.38 | (1.14-1.68) |
|  |  | Poor (≥4 poor factors) | 34/89 | (38.2) | 1.64 | (1.25-2.15) |
|  | High | Ideal (≤1 poor factors) | 155/427 | (36.3) | 1.59 | (1.34-1.88) |
|  |  | Intermediate (2-3 poor factors) | 130/348 | (37.4) | 1.81 | (1.52-2.15) |
|  |  | Poor (≥4 poor factors) | 42/108 | (38.9) | 2.04 | (1.55-2.67) |

CI, confidence interval; LS means, least square means; SBP, systolic blood pressure

The model was adjusted for age, sex, and the first ten principal components.

Hypertension was defined as systolic/diastolic blood pressure of 140/90mmHg or higher measured at a community-support center and/or self-reported treatment for hypertension.

Lifestyle is categorized as ideal (having at least three ideal lifestyle factors), poor (having 0–1 ideal lifestyle factors), intermediate (having 2–3 ideal lifestyle factors).

Lifestyle score included the following factors: Obesity, defined as BMI ≥25.0 kg/m^2^ based on the Western Pacific Region of World Health Organization criteria in Japanese individuals, high Na/K ratio, defined as ≥ 4.0, insufficient regular physical activity, defined as not meeting the American Heart Association recommendations of at least 150 min of moderate activity per week or 75min of vigorous activity per week, smoking, defined as ex-smoker or current smoker, drinking, defined as ex-drinker or current drinker.

| Supplemental Table 9. Association between genetic and lifestyle risk with hypertension incidence in participants Using PRS Constructed with a P-value Threshold < 0.1 | | | | | | |
| --- | --- | --- | --- | --- | --- | --- |
| Family history | Genetic risk | Lifestyle score | case /number of participants | % | RR, 95% CI | |
| No | Low | Ideal (≤1 poor factors) | 198/784 | (25.3) | Ref | |
|  |  | Intermediate (2-3 poor factors) | 209/679 | (30.8) | 1.17 | (0.99-1.38) |
|  |  | Poor (≥4 poor factors) | 66/167 | (32.9) | 1.26 | (0.98-1.62) |
|  | Intermediate | Ideal (≤1 poor factors) | 221/734 | (30.1) | 1.20 | (1.02-1.41) |
|  |  | Intermediate (2-3 poor factors) | 178/608 | (29.3) | 1.22 | (1.03-1.44) |
|  |  | Poor (≥4 poor factors) | 73/187 | (39.0) | 1.69 | (1.36-2.11) |
|  | High | Ideal (≤1 poor factors) | 194/644 | (30.1) | 1.25 | (1.06-1.47) |
|  |  | Intermediate (2-3 poor factors) | 179/605 | (29.6) | 1.24 | (1.05-1.46) |
|  |  | Poor (≥4 poor factors) | 85/188 | (45.2) | 1.76 | (1.44-2.16) |
| Yes | Low | Ideal (≤1 poor factors) | 94/345 | (27.2) | 1.17 | (0.91-1.82) |
|  |  | Intermediate (2-3 poor factors) | 86/316 | (27.2) | 1.29 | (1.04-1.59)- |
|  |  | Poor (≥4 poor factors) | 22/74 | (29.7) | 1.29 | (0.91-1.82) |
|  | Intermediate | Ideal (≤1 poor factors) | 113/364 | (31.0) | 1.34 | (1.11-1.62) |
|  |  | Intermediate (2-3 poor factors) | 92/332 | (27.7) | 1.30 | (1.06-1.60) |
|  |  | Poor (≥4 poor factors) | 35/81 | (43.2) | 2.04 | (1.53-2.71) |
|  | High | Ideal (≤1 poor factors) | 160/426 | (37.6) | 1.72 | (1.45-2.04) |
|  |  | Intermediate (2-3 poor factors) | 130/350 | (37.1) | 1.83 | (1.53-2.19) |
|  |  | Poor (≥4 poor factors) | 42/114 | (36.8) | 1.92 | (1.47-2.51) |

CI, confidence interval; LS means, least square means; SBP, systolic blood pressure

The model was adjusted for age, sex, and the first ten principal components.

Hypertension was defined as systolic/diastolic blood pressure of 140/90mmHg or higher measured at a community-support center and/or self-reported treatment for hypertension.

Lifestyle is categorized as ideal (having at least three ideal lifestyle factors), poor (having 0–1 ideal lifestyle factors), intermediate (having 2–3 ideal lifestyle factors).

Lifestyle score included the following factors: Obesity, defined as BMI ≥25.0 kg/m^2^ based on the Western Pacific Region of World Health Organization criteria in Japanese individuals, high Na/K ratio, defined as ≥ 4.0, insufficient regular physical activity, defined as not meeting the American Heart Association recommendations of at least 150 min of moderate activity per week or 75min of vigorous activity per week, smoking, defined as ex-smoker or current smoker, drinking, defined as ex-drinker or current drinker.

| Supplemental Table 10. Association between genetic and lifestyle risk with hypertension incidence in participants Using PRS Constructed with a P-value Threshold < 0.2 | | | | | | |
| --- | --- | --- | --- | --- | --- | --- |
| Family history | Genetic risk | Lifestyle score | case /number of participants | % | RR, 95% CI | |
| No | Low | Ideal (≤1 poor factors) | 203/801 | (25.3) | Ref | |
|  |  | Intermediate (2-3 poor factors) | 201/666 | (30.2) | 1.16 | (0.99-1.36) |
|  |  | Poor (≥4 poor factors) | 61/173 | (35.3) | 1.41 | (1.11-1.79) |
|  | Intermediate | Ideal (≤1 poor factors) | 218/713 | (30.6) | 1.26 | (1.07-1.47) |
|  |  | Intermediate (2-3 poor factors) | 189/630 | (30.0) | 1.23 | (1.05-1.45) |
|  |  | Poor (≥4 poor factors) | 61/179 | (34.1) | 1.45 | (1.14-1.85) |
|  | High | Ideal (≤1 poor factors) | 192/648 | (29.6) | 1.22 | (1.03-1.44) |
|  |  | Intermediate (2-3 poor factors) | 176/596 | (29.5) | 1.26 | (1.06-1.49) |
|  |  | Poor (≥4 poor factors) | 91/190 | (47.9) | 1.86 | (1.54-2.26) |
| Yes | Low | Ideal (≤1 poor factors) | 98/342 | (28.7) | 1.26 | (1.03-1.53) |
|  |  | Intermediate (2-3 poor factors) | 83/318 | (26.1) | 1.23 | (0.99-1.52) |
|  |  | Poor (≥4 poor factors) | 21/70 | (30.0) | 1.28 | (0.91-1.80) |
|  | Intermediate | Ideal (≤1 poor factors) | 103/350 | (29.4) | 1.26 | (1.04-1.54) |
|  |  | Intermediate (2-3 poor factors) | 100/334 | (29.9) | 1.42 | (1.17-1.74) |
|  |  | Poor (≥4 poor factors) | 38/85 | (44.7) | 2.17 | (1.66-2.82) |
|  | High | Ideal (≤1 poor factors) | 166/443 | (37.5) | 1.72 | (1.46-2.04) |
|  |  | Intermediate (2-3 poor factors) | 125/346 | (36.1) | 1.80 | (1.50-2.15) |
|  |  | Poor (≥4 poor factors) | 40/114 | (35.1) | 1.83 | (1.38-2.43) |

CI, confidence interval; LS means, least square means; SBP, systolic blood pressure

The model was adjusted for age, sex, and the first ten principal components.

Hypertension was defined as systolic/diastolic blood pressure of 140/90mmHg or higher measured at a community-support center and/or self-reported treatment for hypertension.

Lifestyle is categorized as ideal (having at least three ideal lifestyle factors), poor (having 0–1 ideal lifestyle factors), intermediate (having 2–3 ideal lifestyle factors).

Lifestyle score included the following factors: Obesity, defined as BMI ≥25.0 kg/m^2^ based on the Western Pacific Region of World Health Organization criteria in Japanese individuals, high Na/K ratio, defined as ≥ 4.0, insufficient regular physical activity, defined as not meeting the American Heart Association recommendations of at least 150 min of moderate activity per week or 75min of vigorous activity per week, smoking, defined as ex-smoker or current smoker, drinking, defined as ex-drinker or current drinker.

| Supplemental Table 11. Association between genetic and lifestyle risk with hypertension incidence in participants Using PRS Constructed with a P-value Threshold < 0.3 | | | | | | |
| --- | --- | --- | --- | --- | --- | --- |
| Family history | Genetic risk | Lifestyle score | case /number of participants | % | RR, 95% CI | |
| No | Low | Ideal (≤1 poor factors) | 204/787 | (25.9) | Ref | |
|  |  | Intermediate (2-3 poor factors) | 196/677 | (29.0) | 1.08 | (0.92-1.27) |
|  |  | Poor (≥4 poor factors) | 64/175 | (36.6) | 1.38 | (1.09-1.74) |
|  | Intermediate | Ideal (≤1 poor factors) | 209/728 | (28.7) | 1.12 | (0.96-1.32) |
|  |  | Intermediate (2-3 poor factors) | 192/627 | (30.6) | 1.22 | (1.04-1.44) |
|  |  | Poor (≥4 poor factors) | 60/180 | (33.3) | 1.43 | (1.12-1.82) |
|  | High | Ideal (≤1 poor factors) | 200/647 | (30.9) | 1.24 | (1.06-1.46) |
|  |  | Intermediate (2-3 poor factors) | 178/588 | (30.3) | 1.24 | (1.05-1.47) |
|  |  | Poor (≥4 poor factors) | 89/187 | (47.6) | 1.79 | (1.47-2.18) |
| Yes | Low | Ideal (≤1 poor factors) | 100/348 | (28.7) | 1.21 | (0.99-1.47) |
|  |  | Intermediate (2-3 poor factors) | 79/316 | (25.0) | 1.15 | (0.93-1.44) |
|  |  | Poor (≥4 poor factors) | 230/69 | (29.0) | 1.27 | (0.89-1.84) |
|  | Intermediate | Ideal (≤1 poor factors) | 96/331 | (29.0) | 1.20 | (0.98-1.47) |
|  |  | Intermediate (2-3 poor factors) | 101/335 | (30.1) | 1.39 | (1.14-1.70) |
|  |  | Poor (≥4 poor factors) | 42/98 | (42.9) | 1.87 | (1.44-2.42) |
|  | High | Ideal (≤1 poor factors) | 171/456 | (37.5) | 1.69 | (1.43-1.99) |
|  |  | Intermediate (2-3 poor factors) | 128/347 | (36.9) | 1.75 | (1.47-2.09) |
|  |  | Poor (≥4 poor factors) | 37/102 | (36.3) | 1.90 | (1.43-2.54) |

CI, confidence interval; LS means, least square means; SBP, systolic blood pressure

The model was adjusted for age, sex, and the first ten principal components.

Hypertension was defined as systolic/diastolic blood pressure of 140/90mmHg or higher measured at a community-support center and/or self-reported treatment for hypertension.

Lifestyle is categorized as ideal (having at least three ideal lifestyle factors), poor (having 0–1 ideal lifestyle factors), intermediate (having 2–3 ideal lifestyle factors).

Lifestyle score included the following factors: Obesity, defined as BMI ≥25.0 kg/m^2^ based on the Western Pacific Region of World Health Organization criteria in Japanese individuals, high Na/K ratio, defined as ≥ 4.0, insufficient regular physical activity, defined as not meeting the American Heart Association recommendations of at least 150 min of moderate activity per week or 75min of vigorous activity per week, smoking, defined as ex-smoker or current smoker, drinking, defined as ex-drinker or current drinker.

| Supplemental Table 12. Association between genetic and lifestyle risk with hypertension incidence in participants Using PRS Constructed with a P-value Threshold < 0.4 | | | | | | |
| --- | --- | --- | --- | --- | --- | --- |
| Family history | Genetic risk | Lifestyle score | case /number of participants | % | RR, 95% CI | |
| No | Low | Ideal (≤1 poor factors) | 202/791 | (25.5) | Ref | |
|  |  | Intermediate (2-3 poor factors) | 196/664 | (29.5) | 1.14 | (0.96-1.34) |
|  |  | Poor (≥4 poor factors) | 65/177 | (36.7) | 1.43 | (1.14-1.81) |
|  | Intermediate | Ideal (≤1 poor factors) | 221/732 | (30.2) | 1.20 | (1.03-1.41) |
|  |  | Intermediate (2-3 poor factors) | 195/630 | (31.0) | 1.23 | (1.05-1.44) |
|  |  | Poor (≥4 poor factors) | 55/172 | (32.0) | 1.33 | (1.03-1.71) |
|  | High | Ideal (≤1 poor factors) | 190/639 | (29.7) | 1.23 | (1.04-1.45) |
|  |  | Intermediate (2-3 poor factors) | 175/598 | (29.3) | 1.23 | (1.04-1.46) |
|  |  | Poor (≥4 poor factors) | 93/193 | (48.2) | 1.88 | (1.55-2.29) |
| Yes | Low | Ideal (≤1 poor factors) | 100/345 | (29.0) | 1.23 | (1.01-1.49) |
|  |  | Intermediate (2-3 poor factors) | 76/310 | (24.5) | 1.15 | (0.92-1.44) |
|  |  | Poor (≥4 poor factors) | 22/70 | (31.4) | 1.46 | (1.03-2.05) |
|  | Intermediate | Ideal (≤1 poor factors) | 103/343 | (30.0) | 1.29 | (1.06-1.57) |
|  |  | Intermediate (2-3 poor factors) | 106/345 | (30.7) | 1.43 | (1.18-1.73) |
|  |  | Poor (≥4 poor factors) | 43/99 | (43.4) | 1.89 | (1.46-2.44) |
|  | High | Ideal (≤1 poor factors) | 164/447 | (36.7) | 1.68 | (1.41-1.98) |
|  |  | Intermediate (2-3 poor factors) | 126/343 | (36.7) | 1.80 | (1.50-2.15) |
|  |  | Poor (≥4 poor factors) | 34/100 | (34.0) | 1.83 | (1.35-2.47) |

CI, confidence interval; LS means, least square means; SBP, systolic blood pressure

The model was adjusted for age, sex, and the first ten principal components.

Hypertension was defined as systolic/diastolic blood pressure of 140/90mmHg or higher measured at a community-support center and/or self-reported treatment for hypertension.

Lifestyle is categorized as ideal (having at least three ideal lifestyle factors), poor (having 0–1 ideal lifestyle factors), intermediate (having 2–3 ideal lifestyle factors).

Lifestyle score included the following factors: Obesity, defined as BMI ≥25.0 kg/m^2^ based on the Western Pacific Region of World Health Organization criteria in Japanese individuals, high Na/K ratio, defined as ≥ 4.0, insufficient regular physical activity, defined as not meeting the American Heart Association recommendations of at least 150 min of moderate activity per week or 75min of vigorous activity per week, smoking, defined as ex-smoker or current smoker, drinking, defined as ex-drinker or current drinker.

| Supplemental Table 13. Association between genetic and lifestyle risk with hypertension incidence in participants Using PRS Constructed with a P-value Threshold < 0.5 | | | | | | |
| --- | --- | --- | --- | --- | --- | --- |
| Family history | Genetic risk | Lifestyle score | case /number of participants | % | RR, 95% CI | |
| No | Low | Ideal (≤1 poor factors) | 195/781 | (25.0) | Ref | |
|  |  | Intermediate (2-3 poor factors) | 200/680 | (29.4) | 1.16 | (0.99-1.37) |
|  |  | Poor (≥4 poor factors) | 65/174 | (37.4) | 1.47 | (1.17-1.85) |
|  | Intermediate | Ideal (≤1 poor factors) | 228/747 | (30.5) | 1.23 | (1.05-1.45) |
|  |  | Intermediate (2-3 poor factors) | 184/600 | (30.7) | 1.22 | (1.04-1.44) |
|  |  | Poor (≥4 poor factors) | 57/173 | (32.9) | 1.45 | (1.13-1.87) |
|  | High | Ideal (≤1 poor factors) | 190/634 | (30.0) | 1.26 | (1.06-1.49) |
|  |  | Intermediate (2-3 poor factors) | 182/612 | (29.7) | 1.29 | (1.09-1.52) |
|  |  | Poor (≥4 poor factors) | 91/195 | (46.7) | 1.84 | (1.51-2.24) |
| Yes | Low | Ideal (≤1 poor factors) | 96/343 | (28.0) | 1.22 | (0.99-1.49) |
|  |  | Intermediate (2-3 poor factors) | 79/306 | (25.8) | 1.22 | (0.98-1.53) |
|  |  | Poor (≥4 poor factors) | 21/68 | (30.9) | 1.43 | (1.00-2.04) |
|  | Intermediate | Ideal (≤1 poor factors) | 108/344 | (31.4) | 1.37 | (1.12-1.66) |
|  |  | Intermediate (2-3 poor factors) | 102/348 | (29.3) | 1.40 | (1.15-1.71) |
|  |  | Poor (≥4 poor factors) | 45/102 | (44.1) | 1.97 | (1.53-2.53) |
|  | High | Ideal (≤1 poor factors) | 163/448 | (36.4) | 1.68 | (1.42-2.00) |
|  |  | Intermediate (2-3 poor factors) | 127/344 | (36.9) | 1.84 | (1.54-2.21) |
|  |  | Poor (≥4 poor factors) | 33/99 | (33.3) | 1.85 | (1.36-2.52) |

CI, confidence interval; LS means, least square means; SBP, systolic blood pressure

The model was adjusted for age, sex, and the first ten principal components.

Hypertension was defined as systolic/diastolic blood pressure of 140/90mmHg or higher measured at a community-support center and/or self-reported treatment for hypertension.

Lifestyle is categorized as ideal (having at least three ideal lifestyle factors), poor (having 0–1 ideal lifestyle factors), intermediate (having 2–3 ideal lifestyle factors).

Lifestyle score included the following factors: Obesity, defined as BMI ≥25.0 kg/m^2^ based on the Western Pacific Region of World Health Organization criteria in Japanese individuals, high Na/K ratio, defined as ≥ 4.0, insufficient regular physical activity, defined as not meeting the American Heart Association recommendations of at least 150 min of moderate activity per week or 75min of vigorous activity per week, smoking, defined as ex-smoker or current smoker, drinking, defined as ex-drinker or current drinker.

| Supplemental Table 14. Association between genetic and lifestyle risk with hypertension incidence in men | | | | | | |
| --- | --- | --- | --- | --- | --- | --- |
| Family history | Genetic risk | Lifestyle score | case /number of participants | % | RR, 95% CI | |
| No | Low | Ideal (≤1 poor factors) | 51/142 | (35.9) | Ref | |
|  |  | Intermediate (2-3 poor factors) | 179/471 | (38.0) | 1.06 | (0.83-1.35) |
|  |  | Poor (≥4 poor factors) | 45/116 | (38.8) | 1.17 | (0.85-1.59) |
|  | Intermediate | Ideal (≤1 poor factors) | 32/83 | (38.6) | 1.18 | (0.83-1.67) |
|  |  | Intermediate (2-3 poor factors) | 152/438 | (34.7) | 1.05 | (0.81-1.35) |
|  |  | Poor (≥4 poor factors) | 52/122 | (42.6) | 1.30 | (0.96-1.75) |
|  | High | Ideal (≤1 poor factors) | 15/58 | (25.9) | 0.80 | (0.48-1.33) |
|  |  | Intermediate (2-3 poor factors) | 176/408 | (43.1) | 1.30 | (1.02-1.66) |
|  |  | Poor (≥4 poor factors) | 69/139 | (49.6) | 1.56 | (1.19-2.05) |
| Yes | Low | Ideal (≤1 poor factors) | 13/38 | (34.2) | 1.00 | (0.61-1.65) |
|  |  | Intermediate (2-3 poor factors) | 49/168 | (29.2) | 0.92 | (0.67-1.27) |
|  |  | Poor (≥4 poor factors) | 13/41 | (31.7) | 1.14 | (0.70-1.87) |
|  | Intermediate | Ideal (≤1 poor factors) | 11/24 | (45.8) | 1.26 | (0.76-2.08) |
|  |  | Intermediate (2-3 poor factors) | 66/139 | (47.5) | 1.49 | (1.13-1.96) |
|  |  | Poor (≥4 poor factors) | 24/57 | (42.1) | 1.29 | (0.88-1.88) |
|  | High | Ideal (≤1 poor factors) | 9/32 | (28.1) | 0.88 | (0.48-1.62) |
|  |  | Intermediate (2-3 poor factors) | 74/157 | (47.1) | 1.52 | (1.16-2.00) |
|  |  | Poor (≥4 poor factors) | 34/50 | (68.0) | 2.27 | (1.69-3.07) |

CI, confidence interval; LS means, least square means; SBP, systolic blood pressure

The model was adjusted for age, sex, and the first ten principal components.

Hypertension was defined as systolic/diastolic blood pressure of 140/90mmHg or higher measured at a community-support center and/or self-reported treatment for hypertension.

Lifestyle is categorized as ideal (having at least three ideal lifestyle factors), poor (having 0–1 ideal lifestyle factors), intermediate (having 2–3 ideal lifestyle factors).

Lifestyle score included the following factors: Obesity, defined as BMI ≥25.0 kg/m^2^ based on the Western Pacific Region of World Health Organization criteria in Japanese individuals, high Na/K ratio, defined as ≥ 4.0, insufficient regular physical activity, defined as not meeting the American Heart Association recommendations of at least 150 min of moderate activity per week or 75min of vigorous activity per week, smoking, defined as ex-smoker or current smoker, drinking, defined as ex-drinker or current drinker.

| Supplemental Table 15. Association between genetic and lifestyle risk with hypertension incidence in women | | | | | | |
| --- | --- | --- | --- | --- | --- | --- |
| Family history | Genetic risk | Lifestyle score | case /number of participants | % | RR, 95% CI | |
| No | Low | Ideal (≤1 poor factors) | 184/707 | (26.0) | Ref | |
|  |  | Intermediate (2-3 poor factors) | 140/563 | (24.9) | 1.11 | (0.93-1.34) |
|  |  | Poor (≥4 poor factors) | 7/39 | (17.9) | 1.07 | (0.56-2.02) |
|  | Intermediate | Ideal (≤1 poor factors) | 167/645 | (25.9) | 1.02 | (0.86-1.22) |
|  |  | Intermediate (2-3 poor factors) | 160/666 | (24.0) | 1.11 | (0.93-1.33) |
|  |  | Poor (≥4 poor factors) | 19/66 | (28.8) | 1.87 | (1.28-2.75) |
|  | High | Ideal (≤1 poor factors) | 164/527 | (31.1) | 1.27 | (1.07-1.51) |
|  |  | Intermediate (2-3 poor factors) | 175/683 | (25.6) | 1.22 | (1.02-1.45) |
|  |  | Poor (≥4 poor factors) | 21/60 | (35.0) | 2.01 | (1.37-2.95) |
| Yes | Low | Ideal (≤1 poor factors) | 119/365 | (32.6) | 1.39 | (1.15-1.68) |
|  |  | Intermediate (2-3 poor factors) | 87/331 | (26.3) | 1.34 | (1.08-1.66) |
|  |  | Poor (≥4 poor factors) | 4/20 | (20.0) | 1.30 | (0.55-3.09) |
|  | Intermediate | Ideal (≤1 poor factors) | 93/314 | (29.6) | 1.26 | (1.03-1.55) |
|  |  | Intermediate (2-3 poor factors) | 122/406 | (30.0) | 1.57 | (1.29-1.90) |
|  |  | Poor (≥4 poor factors) | 10/40 | (25.0) | 1.82 | (1.06-3.13) |
|  | High | Ideal (≤1 poor factors) | 122/362 | (33.7) | 1.49 | (1.23-1.80) |
|  |  | Intermediate (2-3 poor factors) | 150/463 | (32.4) | 1.73 | (1.44-2.07) |
|  |  | Poor (≥4 poor factors) | 14/61 | (23.0) | 1.47 | (0.92-2.36) |

CI, confidence interval; LS means, least square means; SBP, systolic blood pressure

The model was adjusted for age, sex, and the first ten principal components.

Hypertension was defined as systolic/diastolic blood pressure of 140/90mmHg or higher measured at a community-support center and/or self-reported treatment for hypertension.

Lifestyle is categorized as ideal (having at least three ideal lifestyle factors), poor (having 0–1 ideal lifestyle factors), intermediate (having 2–3 ideal lifestyle factors).

Lifestyle score included the following factors: Obesity, defined as BMI ≥25.0 kg/m^2^ based on the Western Pacific Region of World Health Organization criteria in Japanese individuals, high Na/K ratio, defined as ≥ 4.0, insufficient regular physical activity, defined as not meeting the American Heart Association recommendations of at least 150 min of moderate activity per week or 75min of vigorous activity per week, smoking, defined as ex-smoker or current smoker, drinking, defined as ex-drinker or current drinker.

| Supplemental Table 16. Summary of PRSs included in the polygenic risk score calculation | | | | | | | | |
| --- | --- | --- | --- | --- | --- | --- | --- | --- |
| CHR | SNP | BP | P | rsid | REF | ALT | SE | BETA |
| 12 | 12:112241766_G_A | 112241766 | 6E-56 | rs671 | G | A | 0.00388378 | 0.0615904 |
| 12 | 12:112736118_A_G | 112736118 | 3.2E-51 | rs77768175 | A | G | 0.00449803 | 0.0684523 |
| 12 | 12:111414461_T_G | 111414461 | 3.1E-33 | rs12229654 | T | G | 0.00417639 | 0.0504329 |
| 12 | 12:111705893_A_G | 111705893 | 8.5E-30 | rs3858704 | A | G | 0.00358382 | -0.0411341 |
| 4 | 4:81174592_G_A | 81174592 | 2.9E-27 | rs13125101 | G | A | 0.00352747 | -0.0387704 |
| 12 | 12:89989599_G_A | 89989599 | 1.1E-26 | rs1401982 | G | A | 0.00345175 | -0.0372519 |
| 12 | 12:113045654_C_T | 113045654 | 3E-26 | rs11066359 | C | T | 0.0039266 | 0.042339 |
| 12 | 12:111942493_T_C | 111942493 | 5.5E-24 | rs11065933 | T | C | 0.00351266 | 0.0362547 |
| 2 | 2:26932031_C_T | 26932031 | 3.1E-23 | rs2586886 | C | T | 0.00406206 | 0.0410333 |
| 10 | 10:104958900_G_A | 104958900 | 2.3E-19 | rs112913898 | G | A | 0.00387128 | 0.0348413 |
| 1 | 1:10796547_G_A | 10796547 | 5.1E-19 | rs17035646 | G | A | 0.00357452 | -0.0327724 |
| 12 | 12:113196733_G_A | 113196733 | 3E-17 | rs11614295 | G | A | 0.00378845 | 0.0320517 |
| 10 | 10:104682602_G_A | 104682602 | 5.1E-17 | rs77602510 | G | A | 0.00377438 | 0.0316086 |
| 19 | 19:11526765_G_T | 11526765 | 6.9E-17 | 19:11526765:G:T | G | T | 0.00339708 | 0.0291776 |
| 12 | 12:115553034_G_A | 115553034 | 3.5E-14 | rs35442 | G | A | 0.00381354 | 0.0292885 |
| 3 | 3:53590465_C_T | 53590465 | 6.8E-14 | rs9814480 | C | T | 0.00507763 | -0.0387302 |
| 1 | 1:113046879_C_A | 113046879 | 3.9E-13 | rs3790604 | C | A | 0.00369119 | -0.0273165 |
| 2 | 2:165005726_T_C | 165005726 | 1.7E-12 | rs10194493 | T | C | 0.00339391 | 0.0246367 |
| 8 | 8:144035499_G_A | 144035499 | 2.3E-12 | rs28469769 | G | A | 0.00348591 | 0.0237962 |
| 6 | 6:43279721_G_A | 43279721 | 3.3E-12 | rs4398731 | G | A | 0.00349051 | -0.0246723 |
| 12 | 12:113891729_T_C | 113891729 | 4.8E-12 | rs11066566 | T | C | 0.00562574 | 0.0396554 |
| 11 | 11:100579854_C_T | 100579854 | 6.2E-12 | rs1847149 | C | T | 0.00334488 | -0.022255 |
| 3 | 3:27562988_C_T | 27562988 | 8E-12 | rs2643826 | C | T | 0.00386834 | -0.0267769 |
| 12 | 12:111673387_T_C | 111673387 | 1E-11 | rs4766452 | T | C | 0.0034064 | 0.0236304 |
| 12 | 12:113263518_A_C | 113263518 | 1.4E-11 | 12:113263518:A:C | A | C | 0.00334012 | -0.0228562 |
| 17 | 17:78517950_G_A | 78517950 | 2.6E-11 | rs112220804 | G | A | 0.0161555 | -0.10593 |
| 12 | 12:110697330_G_A | 110697330 | 2.9E-11 | rs117121174 | G | A | 0.00581991 | 0.0389858 |
| 7 | 7:27242617_T_C | 27242617 | 1.1E-10 | 7:27242617:T:C | T | C | 0.00341804 | -0.0214974 |
| 12 | 12:113519816_T_G | 113519816 | 1.9E-10 | rs1732803 | T | G | 0.00365084 | 0.023391 |
| 5 | 5:32832474_C_A | 32832474 | 2.7E-10 | rs10059884 | C | A | 0.00339136 | -0.0223565 |
| 18 | 18:715714_T_C | 715714 | 3.6E-10 | rs6506469 | T | C | 0.00333571 | 0.020949 |
| 11 | 11:9776567_C_A | 9776567 | 3.7E-10 | rs360140 | C | A | 0.00352577 | -0.0219043 |
| 12 | 12:113327018_C_T | 113327018 | 4E-10 | rs7966149 | C | T | 0.00340122 | -0.0215863 |
| 3 | 3:14932502_A_G | 14932502 | 7E-10 | rs17040514 | A | G | 0.00352816 | -0.0222871 |
| 15 | 15:91416550_C_A | 91416550 | 2E-09 | 15:91416550:C:A | C | A | 0.00475205 | -0.0278042 |
| 6 | 6:43153836_G_A | 43153836 | 5.4E-09 | rs141303988 | G | A | 0.0114296 | -0.0667722 |
| 11 | 11:1892585_G_A | 1892585 | 6.6E-09 | rs4980389 | G | A | 0.00393767 | -0.0230438 |
| 12 | 12:112953031_G_A | 112953031 | 7.2E-09 | rs60902179 | G | A | 0.00518463 | -0.0301692 |
| 10 | 10:104361711_C_T | 104361711 | 8.7E-09 | rs3934495 | C | T | 0.00333785 | 0.0193172 |
| 2 | 2:164445182_T_G | 164445182 | 1.2E-08 | rs16848671 | T | G | 0.00335879 | -0.0194778 |
| 2 | 2:55023600_G_A | 55023600 | 1.5E-08 | rs17046344 | G | A | 0.00674635 | -0.0388408 |
| 3 | 3:53594413_A_C | 53594413 | 1.9E-08 | rs56021416 | A | C | 0.00372767 | -0.0220555 |
| 15 | 15:85696481_C_T | 85696481 | 1.9E-08 | rs146435478 | C | T | 0.0135494 | 0.072907 |
| 19 | 19:19266848_C_T | 19266848 | 2.1E-08 | rs7360000 | C | T | 0.00359337 | -0.0215495 |
| 19 | 19:2224387_A_C | 2224387 | 2.3E-08 | rs12459507 | A | C | 0.00375272 | -0.0208472 |
| 12 | 12:112295190_T_C | 112295190 | 3.2E-08 | rs10774640 | T | C | 0.00405265 | 0.0218544 |
| 6 | 6:31330969_C_A | 31330969 | 3.4E-08 | rs2523559 | C | A | 0.00475403 | 0.0270292 |
| 12 | 12:110390979_T_C | 110390979 | 4.5E-08 | rs925368 | T | C | 0.00590959 | 0.0322304 |
| 20 | 20:42797358_A_G | 42797358 | 5E-08 | rs6031435 | A | G | 0.00364284 | -0.020388 |
| 20 | 20:50110558_T_G | 50110558 | 5E-08 | rs3787192 | T | G | 0.00341022 | -0.0188692 |
| 12 | 12:133099570_G_A | 133099570 | 6.6E-08 | rs74971075 | G | A | 0.00567053 | -0.0314495 |
| 17 | 17:59241155_T_C | 59241155 | 6.9E-08 | rs8073894 | T | C | 0.0038146 | 0.020994 |
| 2 | 2:240251634_C_T | 240251634 | 7.1E-08 | rs62182108 | C | T | 0.0034446 | -0.017429 |
| 12 | 12:112475403_T_C | 112475403 | 8.2E-08 | rs77419179 | T | C | 0.00749148 | -0.0413215 |
| 8 | 8:76844491_G_A | 76844491 | 9E-08 | rs1462446 | G | A | 0.00333798 | 0.0180199 |
| 6 | 6:127175523_A_C | 127175523 | 9.2E-08 | rs4897192 | A | C | 0.0034204 | -0.0190022 |
| 6 | 6:32409242_C_A | 32409242 | 1E-07 | rs3135392 | C | A | 0.00353172 | -0.0188048 |
| 10 | 10:96038686_G_A | 96038686 | 1E-07 | rs11187838 | G | A | 0.00334583 | 0.0173948 |
| 7 | 7:1092005_C_T | 1092005 | 1.3E-07 | rs76654767 | C | T | 0.0039093 | -0.019855 |
| 3 | 3:53842251_C_T | 53842251 | 1.4E-07 | rs62250902 | C | T | 0.00446108 | 0.0239972 |
| 12 | 12:111773687_A_G | 111773687 | 1.7E-07 | rs58250653 | A | G | 0.0048276 | -0.0271452 |
| 7 | 7:27332936_C_T | 27332936 | 1.8E-07 | rs9770721 | C | T | 0.00375103 | -0.0195396 |
| 4 | 4:81177929_A_G | 81177929 | 1.9E-07 | rs17004853 | A | G | 0.0036884 | 0.0189854 |
| 2 | 2:54734484_G_A | 54734484 | 2E-07 | rs139100345 | G | A | 0.00790405 | -0.0416477 |
| 4 | 4:106910958_C_T | 106910958 | 2E-07 | rs56388530 | C | T | 0.00353863 | -0.018839 |
| 10 | 10:115722736_A_C | 115722736 | 2E-07 | rs527979842 | A | C | 0.00553189 | 0.0296546 |
| 6 | 6:31106893_A_G | 31106893 | 2.1E-07 | rs1265094 | A | G | 0.00347034 | 0.0187425 |
| 12 | 12:112186643_T_C | 112186643 | 2.1E-07 | rs79290585 | T | C | 0.00751885 | -0.0399519 |
| 13 | 13:41522272_T_G | 41522272 | 2.3E-07 | rs75719223 | T | G | 0.0034681 | 0.0175719 |
| 10 | 10:105671683_C_T | 105671683 | 2.4E-07 | rs79342925 | C | T | 0.00626295 | -0.0309973 |
| 3 | 3:63537061_C_T | 63537061 | 3E-07 | rs146275468 | C | T | 0.0125871 | 0.0657872 |
| 12 | 12:111623270_G_A | 111623270 | 3E-07 | rs75956519 | G | A | 0.00873214 | -0.0457938 |
| 12 | 12:12877692_T_C | 12877692 | 3.2E-07 | rs34325 | T | C | 0.00334804 | -0.0166748 |
| 4 | 4:86716496_T_G | 86716496 | 3.4E-07 | rs6829822 | T | G | 0.00403448 | 0.0200318 |
| 15 | 15:74222043_A_G | 74222043 | 3.4E-07 | rs8034017 | A | G | 0.00463186 | -0.0238071 |
| 12 | 12:113006345_C_T | 113006345 | 3.9E-07 | rs6489849 | C | T | 0.00513268 | 0.0257491 |
| 3 | 3:53657992_G_A | 53657992 | 4E-07 | 3:53657992:G:A | G | A | 0.00353329 | -0.0180333 |
| 3 | 3:27501249_C_T | 27501249 | 4.2E-07 | rs148397457 | C | T | 0.00939151 | -0.0501027 |
| 3 | 3:53617916_A_G | 53617916 | 4.5E-07 | rs537945357 | A | G | 0.012942 | -0.0664234 |
| 6 | 6:27773664_T_C | 27773664 | 4.7E-07 | rs200480 | T | C | 0.00335015 | -0.016925 |
| 5 | 5:124303176_G_A | 124303176 | 4.9E-07 | rs35879477 | G | A | 0.00335263 | -0.016502 |
| 8 | 8:76570956_T_G | 76570956 | 6E-07 | rs16939124 | T | G | 0.00465062 | -0.0230653 |
| 7 | 7:27196113_C_T | 27196113 | 6.1E-07 | rs2301721 | C | T | 0.00450554 | 0.0219421 |
| 14 | 14:27574225_C_T | 27574225 | 6.5E-07 | rs12882283 | C | T | 0.00353459 | 0.0179508 |
| 19 | 19:45907522_C_A | 45907522 | 6.5E-07 | rs2336218 | C | A | 0.0033376 | 0.0164441 |
| 12 | 12:89885942_T_C | 89885942 | 6.8E-07 | rs6538190 | T | C | 0.00520489 | 0.0248982 |
| 17 | 17:45687762_G_T | 45687762 | 6.9E-07 | 17:45687762:G:T | G | T | 0.00607665 | -0.0298184 |
| 19 | 19:11442811_T_C | 11442811 | 6.9E-07 | rs448743 | T | C | 0.00377165 | -0.018266 |
| 3 | 3:56950852_G_A | 56950852 | 7.1E-07 | rs6445838 | G | A | 0.00353292 | -0.0177286 |
| 6 | 6:134949581_C_T | 134949581 | 7.2E-07 | rs4323320 | C | T | 0.0050786 | -0.0252835 |
| 12 | 12:80088578_T_C | 80088578 | 7.2E-07 | rs12827748 | T | C | 0.00368343 | 0.0189352 |
| 11 | 11:2019174_C_T | 2019174 | 7.8E-07 | rs76989783 | C | T | 0.00684619 | 0.0330688 |
| 11 | 11:1949470_G_A | 1949470 | 8.5E-07 | rs643753 | G | A | 0.00352212 | -0.0182606 |
| 4 | 4:111338277_T_C | 111338277 | 9.3E-07 | rs9994289 | T | C | 0.00333872 | 0.0162734 |
| 11 | 11:1983282_G_A | 1983282 | 9.7E-07 | rs60220081 | G | A | 0.00457594 | -0.0228943 |
| 1 | 1:27895517_A_G | 27895517 | 9.9E-07 | rs12749647 | A | G | 0.00377321 | 0.0188966 |
| 6 | 6:25179357_T_C | 25179357 | 9.9E-07 | rs55916993 | T | C | 0.00557167 | 0.0266601 |
| 8 | 8:77117692_T_G | 77117692 | 9.9E-07 | rs13271198 | T | G | 0.00332877 | -0.0160929 |
| 17 | 17:64530887_C_A | 64530887 | 1.1E-06 | rs9303509 | C | A | 0.0034014 | -0.0163613 |
| 2 | 2:182168010_C_T | 182168010 | 1.2E-06 | rs186105559 | C | T | 0.0108015 | 0.0531162 |
| 6 | 6:26451553_A_G | 26451553 | 1.2E-06 | 6:26451553:A:G | A | G | 0.00356887 | -0.0179141 |
| 17 | 17:59472123_G_A | 59472123 | 1.2E-06 | rs2286525 | G | A | 0.00345073 | -0.0172583 |
| 3 | 3:51758820_C_T | 51758820 | 1.3E-06 | rs73080658 | C | T | 0.00637172 | -0.0296858 |
| 19 | 19:10680621_G_A | 10680621 | 1.3E-06 | rs1465700 | G | A | 0.00450284 | 0.0213603 |
| 22 | 22:29250505_T_G | 29250505 | 1.3E-06 | rs5762861 | T | G | 0.00336772 | -0.016154 |
| 6 | 6:30788191_T_C | 30788191 | 1.4E-06 | rs3094111 | T | C | 0.00518103 | 0.0261328 |
| 10 | 10:115722411_A_G | 115722411 | 1.4E-06 | rs180940 | A | G | 0.00334558 | -0.0168924 |
| 3 | 3:51503887_C_T | 51503887 | 1.5E-06 | rs73078654 | C | T | 0.00596221 | -0.0276337 |
| 9 | 9:118357126_C_T | 118357126 | 1.5E-06 | rs875069 | C | T | 0.0047475 | -0.0222225 |
| 12 | 12:110138074_A_G | 110138074 | 1.6E-06 | rs147891190 | A | G | 0.0119439 | 0.0566187 |
| 8 | 8:140923682_C_T | 140923682 | 1.7E-06 | rs36002174 | C | T | 0.00385257 | 0.0191309 |
| 10 | 10:104313766_C_T | 104313766 | 1.7E-06 | 10:104313766:C:T | C | T | 0.0109169 | 0.0540777 |
| 6 | 6:32042322_T_C | 32042322 | 1.9E-06 | rs3130286 | T | C | 0.00424313 | -0.0195677 |
| 12 | 12:90215621_G_A | 90215621 | 1.9E-06 | rs7136711 | G | A | 0.00370738 | 0.0175745 |
| 2 | 2:99392745_T_C | 99392745 | 2.2E-06 | rs2276604 | T | C | 0.00347949 | -0.0169884 |
| 11 | 11:133167123_G_A | 133167123 | 2.2E-06 | rs7940198 | G | A | 0.00332327 | 0.0149602 |
| 12 | 12:113490077_G_A | 113490077 | 2.2E-06 | rs2255284 | G | A | 0.0033768 | -0.015405 |
| 12 | 12:115510546_G_A | 115510546 | 2.3E-06 | 12:115510546:G:A | G | A | 0.00742632 | 0.0349159 |
| 20 | 20:57472174_C_T | 57472174 | 2.3E-06 | rs1407040 | C | T | 0.00372706 | 0.0173497 |
| 6 | 6:43509490_T_G | 43509490 | 2.4E-06 | rs117326207 | T | G | 0.0102373 | -0.0485304 |
| 17 | 17:45317499_T_C | 45317499 | 2.4E-06 | rs17605348 | T | C | 0.00565803 | -0.0264821 |
| 5 | 5:174242826_A_C | 174242826 | 2.5E-06 | rs62390991 | A | C | 0.00781621 | 0.0390668 |
| 3 | 3:169477506_A_G | 169477506 | 2.6E-06 | rs12638862 | A | G | 0.00347359 | 0.0172893 |
| 6 | 6:137064933_T_C | 137064933 | 2.7E-06 | rs72983506 | T | C | 0.00335065 | 0.015526 |
| 12 | 12:123611741_G_A | 123611741 | 2.8E-06 | rs11057162 | G | A | 0.00397389 | -0.0172866 |
| 3 | 3:52843337_T_C | 52843337 | 2.9E-06 | rs1041825763 | T | C | 0.0136658 | -0.0641375 |
| 10 | 10:18361329_T_G | 18361329 | 3E-06 | rs2488155 | T | G | 0.00333201 | 0.0154633 |
| 3 | 3:27486065_C_T | 27486065 | 3.1E-06 | rs28407792 | C | T | 0.00757962 | -0.0363133 |
| 5 | 5:158220193_A_G | 158220193 | 3.1E-06 | rs31864 | A | G | 0.00351641 | 0.0168424 |
| 3 | 3:53861137_A_G | 53861137 | 3.3E-06 | rs4687590 | A | G | 0.00336146 | 0.0160433 |
| 16 | 16:53845169_T_G | 53845169 | 3.4E-06 | rs11649091 | T | G | 0.00413868 | -0.0190743 |
| 2 | 2:162592328_T_C | 162592328 | 3.6E-06 | rs1006427 | T | C | 0.00537834 | 0.0241761 |
| 1 | 1:113064468_G_A | 113064468 | 3.7E-06 | rs6668533 | G | A | 0.007214 | 0.0342734 |
| 14 | 14:76569483_T_C | 76569483 | 3.8E-06 | rs11159173 | T | C | 0.00336214 | 0.0151681 |
| 4 | 4:15387255_G_A | 15387255 | 3.9E-06 | rs13146941 | G | A | 0.00359793 | 0.0172052 |
| 7 | 7:33861180_C_A | 33861180 | 3.9E-06 | rs7796633 | C | A | 0.00417768 | -0.0211876 |
| 17 | 17:46953258_A_G | 46953258 | 3.9E-06 | rs55762970 | A | G | 0.00856407 | 0.0410607 |
| 15 | 15:75070196_A_G | 75070196 | 4.3E-06 | rs12905199 | A | G | 0.00416936 | 0.0197908 |
| 20 | 20:19874222_G_A | 19874222 | 4.3E-06 | rs17301932 | G | A | 0.00346601 | 0.0164985 |
| 20 | 20:54173873_G_A | 54173873 | 4.5E-06 | rs3859599 | G | A | 0.00398091 | -0.0181291 |
| 8 | 8:17428495_G_A | 17428495 | 4.7E-06 | rs6982625 | G | A | 0.00575182 | -0.0258625 |
| 2 | 2:26938216_G_T | 26938216 | 5E-06 | rs1731246 | G | T | 0.0035813 | 0.0165092 |
| 10 | 10:10226730_A_G | 10226730 | 5.1E-06 | rs78414725 | A | G | 0.00794713 | -0.0355516 |
| 3 | 3:53296320_T_C | 53296320 | 5.3E-06 | rs931254 | T | C | 0.00332984 | -0.0156886 |
| 7 | 7:26008233_T_C | 26008233 | 6.1E-06 | rs2391211 | T | C | 0.0035978 | -0.0162165 |
| 9 | 9:90299558_A_C | 90299558 | 6.2E-06 | rs59243137 | A | C | 0.00539428 | 0.0233523 |
| 15 | 15:64271754_C_A | 64271754 | 6.4E-06 | rs12911272 | C | A | 0.00895803 | 0.0398907 |
| 13 | 13:22692292_A_G | 22692292 | 6.5E-06 | rs9316766 | A | G | 0.00342689 | -0.0152287 |
| 8 | 8:95700688_C_T | 95700688 | 6.8E-06 | rs2554399 | C | T | 0.00369341 | 0.0168911 |
| 5 | 5:30897450_C_T | 30897450 | 6.9E-06 | rs10940928 | C | T | 0.00341566 | 0.0148832 |
| 10 | 10:127335782_C_T | 127335782 | 6.9E-06 | rs527313009 | C | T | 0.0103198 | -0.0452785 |
| 16 | 16:56512769_A_G | 56512769 | 7E-06 | rs918784 | A | G | 0.00350447 | -0.0152031 |
| 12 | 12:90301665_C_T | 90301665 | 7.1E-06 | rs4240744 | C | T | 0.00342256 | 0.0142341 |
| 6 | 6:80252244_C_T | 80252244 | 7.4E-06 | rs72901812 | C | T | 0.00410963 | 0.0189282 |
| 3 | 3:50956139_A_G | 50956139 | 7.5E-06 | rs148431393 | A | G | 0.00956004 | -0.0411006 |
| 8 | 8:76312318_A_G | 76312318 | 7.7E-06 | rs55958919 | A | G | 0.00499595 | -0.0224208 |
| 2 | 2:191488020_C_T | 191488020 | 7.8E-06 | rs62182899 | C | T | 0.00361371 | 0.0160017 |
| 11 | 11:89301382_A_G | 89301382 | 7.8E-06 | rs1813212 | A | G | 0.00342658 | 0.0152496 |
| 10 | 10:60293320_A_C | 60293320 | 8E-06 | rs1649078 | A | C | 0.00335581 | -0.0156557 |
| 6 | 6:55845332_A_G | 55845332 | 8.2E-06 | rs146358762 | A | G | 0.012849 | -0.0587876 |
| 6 | 6:126078361_A_G | 126078361 | 8.2E-06 | rs3799709 | A | G | 0.00368737 | -0.0161176 |
| 9 | 9:4129917_C_T | 4129917 | 8.5E-06 | rs1770394 | C | T | 0.00348367 | -0.0159316 |
| 1 | 1:212857905_A_G | 212857905 | 8.8E-06 | rs7533204 | A | G | 0.00370279 | -0.0171424 |
| 4 | 4:109004126_A_C | 109004126 | 8.8E-06 | rs3797000 | A | C | 0.00768454 | 0.0353778 |
| 17 | 17:46672154_T_C | 46672154 | 8.8E-06 | rs6504411 | T | C | 0.00471779 | -0.0209222 |
| 17 | 17:75308306_C_T | 75308306 | 9E-06 | rs9910855 | C | T | 0.00336608 | -0.0151863 |
| 19 | 19:11299384_G_A | 11299384 | 9.1E-06 | rs34307310 | G | A | 0.00939271 | -0.0422577 |
| 13 | 13:31045391_G_A | 31045391 | 9.4E-06 | rs57850142 | G | A | 0.00723484 | 0.0316053 |
| 6 | 6:55589427_T_C | 55589427 | 9.7E-06 | rs143659774 | T | C | 0.0128633 | -0.0577211 |
| 11 | 11:120139487_G_A | 120139487 | 9.7E-06 | rs10892558 | G | A | 0.00389623 | -0.0179928 |
| 2 | 2:98343254_C_T | 98343254 | 1.1E-05 | rs6543040 | C | T | 0.00384057 | -0.016181 |
| 6 | 6:31315648_A_G | 31315648 | 1.1E-05 | rs2523625 | A | G | 0.0041749 | 0.0173928 |
| 12 | 12:54441498_C_T | 54441498 | 1.1E-05 | rs7134677 | C | T | 0.00344877 | 0.014317 |
| 12 | 12:131627985_C_T | 131627985 | 1.1E-05 | rs11061346 | C | T | 0.00877864 | 0.0409338 |
| 16 | 16:49619279_T_C | 49619279 | 1.1E-05 | rs12599722 | T | C | 0.00337016 | 0.014468 |
| 1 | 1:155162067_C_T | 155162067 | 1.2E-05 | rs4072037 | C | T | 0.0044155 | 0.0198013 |
| 6 | 6:33652880_G_A | 33652880 | 1.2E-05 | rs753890 | G | A | 0.0040367 | 0.0178475 |
| 9 | 9:97785248_G_A | 97785248 | 1.2E-05 | rs7023719 | G | A | 0.00564492 | -0.0253561 |
| 10 | 10:123271146_G_A | 123271146 | 1.2E-05 | rs3750815 | G | A | 0.00505034 | 0.0215968 |
| 12 | 12:112648981_T_C | 112648981 | 1.2E-05 | rs10161225 | T | C | 0.00545613 | 0.0229524 |
| 3 | 3:52323220_T_C | 52323220 | 1.3E-05 | rs149554319 | T | C | 0.0131589 | -0.0573646 |
| 4 | 4:139139293_T_C | 139139293 | 1.3E-05 | rs62324387 | T | C | 0.00414607 | -0.0173103 |
| 4 | 4:157191510_C_T | 157191510 | 1.3E-05 | rs139894069 | C | T | 0.01132 | 0.0491478 |
| 5 | 5:1278584_C_T | 1278584 | 1.3E-05 | rs2075785 | C | T | 0.00378057 | 0.0159092 |
| 5 | 5:102474811_A_G | 102474811 | 1.3E-05 | rs17532917 | A | G | 0.00746277 | -0.0316202 |
| 6 | 6:32720219_G_A | 32720219 | 1.3E-05 | rs56144236 | G | A | 0.00535064 | 0.0233344 |
| 6 | 6:43791080_T_C | 43791080 | 1.3E-05 | 6:43791080:T:C | T | C | 0.00477542 | -0.0203711 |
| 2 | 2:26848046_T_C | 26848046 | 1.4E-05 | rs1662992 | T | C | 0.00364083 | 0.0152189 |
| 8 | 8:51695984_A_G | 51695984 | 1.4E-05 | rs11777427 | A | G | 0.0039681 | -0.0167365 |
| 17 | 17:7631336_T_C | 7631336 | 1.4E-05 | rs78541136 | T | C | 0.0130515 | 0.0565505 |
| 7 | 7:27249379_G_A | 27249379 | 1.5E-05 | rs28357162 | G | A | 0.0132914 | -0.0576163 |
| 10 | 10:104834876_C_T | 104834876 | 1.5E-05 | rs17115419 | C | T | 0.00484194 | -0.0227857 |
| 14 | 14:100842377_A_G | 100842377 | 1.5E-05 | rs17554326 | A | G | 0.0053338 | 0.0238695 |
| 5 | 5:148400668_A_G | 148400668 | 1.6E-05 | rs79209537 | A | G | 0.00676442 | 0.0291254 |
| 2 | 2:165195888_G_T | 165195888 | 1.7E-05 | 2:165195888:G:T | G | T | 0.00702321 | 0.0287777 |
| 3 | 3:52069409_C_T | 52069409 | 1.7E-05 | rs191411511 | C | T | 0.0132278 | -0.0558274 |
| 5 | 5:55643642_T_C | 55643642 | 1.7E-05 | rs75804422 | T | C | 0.0108465 | 0.0467868 |
| 8 | 8:76554124_T_C | 76554124 | 1.7E-05 | rs4291242 | T | C | 0.00366286 | -0.015029 |
| 9 | 9:112330129_A_C | 112330129 | 1.7E-05 | rs10979938 | A | C | 0.00427805 | -0.0182431 |
| 14 | 14:70500973_G_T | 70500973 | 1.7E-05 | rs17107651 | G | T | 0.00340028 | -0.0149778 |
| 18 | 18:13009365_T_C | 13009365 | 1.7E-05 | rs150716289 | T | C | 0.0159627 | 0.0629516 |
| 3 | 3:143864465_C_T | 143864465 | 1.8E-05 | rs56406865 | C | T | 0.00610892 | -0.0265564 |
| 4 | 4:88021537_G_T | 88021537 | 1.8E-05 | 4:88021537:G:T | G | T | 0.00834323 | 0.036393 |
| 6 | 6:32113312_G_A | 32113312 | 1.8E-05 | rs205002 | G | A | 0.00357835 | -0.01594 |
| 11 | 11:65384727_C_T | 65384727 | 1.8E-05 | 11:65384727:C:T | C | T | 0.0033416 | 0.0143324 |
| 11 | 11:108046457_T_C | 108046457 | 1.8E-05 | rs77007190 | T | C | 0.00916511 | -0.0382684 |
| 2 | 2:122038783_G_A | 122038783 | 1.9E-05 | rs36096257 | G | A | 0.00779889 | -0.0334706 |
| 3 | 3:168814206_G_A | 168814206 | 1.9E-05 | rs56743174 | G | A | 0.00602274 | 0.0252501 |
| 4 | 4:44639983_T_C | 44639983 | 1.9E-05 | rs149081917 | T | C | 0.0160603 | -0.067706 |
| 6 | 6:32208324_C_T | 32208324 | 1.9E-05 | rs424232 | C | T | 0.00379946 | 0.0163906 |
| 7 | 7:71707388_T_C | 71707388 | 1.9E-05 | rs76345064 | T | C | 0.0127664 | -0.0549513 |
| 11 | 11:13281557_C_A | 13281557 | 1.9E-05 | rs11022733 | C | A | 0.00334555 | -0.0136824 |
| 19 | 19:29369130_G_T | 29369130 | 1.9E-05 | rs4805283 | G | T | 0.00333285 | -0.0154115 |
| 7 | 7:40477363_A_G | 40477363 | 2E-05 | rs1319467 | A | G | 0.00348821 | -0.0137295 |
| 9 | 9:116002565_C_T | 116002565 | 2E-05 | rs7851623 | C | T | 0.005311 | -0.0240325 |
| 10 | 10:134283875_T_C | 134283875 | 2E-05 | rs7897741 | T | C | 0.00498008 | 0.0220698 |
| 11 | 11:61277885_G_A | 61277885 | 2E-05 | rs10897165 | G | A | 0.00333555 | 0.0148091 |
| 8 | 8:137936393_T_G | 137936393 | 2.1E-05 | rs2325909 | T | G | 0.00452036 | 0.0202234 |
| 22 | 22:23505480_G_A | 23505480 | 2.1E-05 | rs142317698 | G | A | 0.0107223 | 0.0456939 |
| 3 | 3:73294988_T_C | 73294988 | 2.2E-05 | rs143927075 | T | C | 0.00868809 | 0.0356282 |
| 6 | 6:1627219_T_C | 1627219 | 2.2E-05 | rs12193783 | T | C | 0.003691 | 0.015745 |
| 8 | 8:80218071_G_A | 80218071 | 2.2E-05 | 8:80218071:G:A | G | A | 0.0147753 | 0.062568 |
| 14 | 14:42328828_T_C | 42328828 | 2.2E-05 | rs144643665 | T | C | 0.0115752 | 0.0469943 |
| 2 | 2:18174391_C_T | 18174391 | 2.3E-05 | rs72782519 | C | T | 0.00561596 | -0.022049 |
| 6 | 6:164540422_G_A | 164540422 | 2.3E-05 | rs35639127 | G | A | 0.011097 | -0.0438127 |
| 1 | 1:93349046_A_G | 93349046 | 2.4E-05 | rs7536563 | A | G | 0.00947804 | 0.0392209 |
| 1 | 1:159045169_A_G | 159045169 | 2.4E-05 | rs75491800 | A | G | 0.00414131 | -0.0169395 |
| 1 | 1:200541573_T_C | 200541573 | 2.4E-05 | rs11584563 | T | C | 0.0033524 | 0.0135596 |
| 2 | 2:184139206_T_C | 184139206 | 2.4E-05 | rs148359138 | T | C | 0.0110596 | -0.04714 |
| 5 | 5:56986160_C_A | 56986160 | 2.4E-05 | rs10487614 | C | A | 0.0109053 | -0.0458323 |
| 10 | 10:104489375_G_A | 104489375 | 2.4E-05 | rs138845717 | G | A | 0.0100081 | 0.042092 |
| 14 | 14:64893313_C_T | 64893313 | 2.4E-05 | rs142595416 | C | T | 0.0122474 | 0.0507644 |
| 9 | 9:6980688_A_C | 6980688 | 2.5E-05 | rs818912 | A | C | 0.00339973 | 0.0141385 |
| 12 | 12:117578161_A_G | 117578161 | 2.5E-05 | rs7302633 | A | G | 0.00341165 | -0.0141792 |
| 15 | 15:51062741_A_G | 51062741 | 2.5E-05 | rs8038437 | A | G | 0.00416164 | 0.0182394 |
| 15 | 15:52696097_G_A | 52696097 | 2.5E-05 | rs8024439 | G | A | 0.00357318 | 0.0154554 |
| 4 | 4:132584076_C_A | 132584076 | 2.6E-05 | rs117906362 | C | A | 0.0144366 | 0.0609328 |
| 5 | 5:114165361_C_A | 114165361 | 2.6E-05 | rs6594839 | C | A | 0.00352053 | 0.0150833 |
| 11 | 11:9692148_A_G | 9692148 | 2.6E-05 | rs73408298 | A | G | 0.00434129 | -0.018853 |
| 16 | 16:24759131_A_G | 24759131 | 2.6E-05 | rs200528 | A | G | 0.00634025 | -0.027511 |
| 16 | 16:72885718_G_A | 72885718 | 2.6E-05 | rs139404768 | G | A | 0.0039863 | -0.0167037 |
| 3 | 3:115043624_A_G | 115043624 | 2.7E-05 | 3:115043624:A:G | A | G | 0.00364423 | -0.0146225 |
| 10 | 10:90984731_C_T | 90984731 | 2.7E-05 | rs2297474 | C | T | 0.00781634 | 0.0310609 |
| 11 | 11:100736508_G_T | 100736508 | 2.7E-05 | rs6590824 | G | T | 0.00366775 | 0.0150148 |
| 17 | 17:57680210_C_T | 57680210 | 2.7E-05 | rs555692562 | C | T | 0.011307 | 0.0476984 |
| 2 | 2:162313036_G_T | 162313036 | 2.8E-05 | rs62188153 | G | T | 0.0055882 | 0.0235407 |
| 3 | 3:47453340_C_T | 47453340 | 2.8E-05 | rs150134286 | C | T | 0.00975492 | -0.04372 |
| 10 | 10:21210283_G_A | 21210283 | 2.8E-05 | rs12356048 | G | A | 0.00336153 | 0.0143367 |
| 19 | 19:17199314_T_C | 17199314 | 2.8E-05 | rs11669463 | T | C | 0.00356299 | -0.0153458 |
| 20 | 20:5577000_C_T | 5577000 | 2.8E-05 | rs6053525 | C | T | 0.00363613 | 0.0154997 |
| 22 | 22:33584336_G_A | 33584336 | 2.8E-05 | rs117473930 | G | A | 0.01538 | 0.0609759 |
| 1 | 1:214318748_C_A | 214318748 | 2.9E-05 | rs10864086 | C | A | 0.00334493 | -0.0135586 |
| 2 | 2:224943376_C_T | 224943376 | 2.9E-05 | rs74825684 | C | T | 0.0116629 | 0.0482443 |
| 19 | 19:4684642_A_G | 4684642 | 2.9E-05 | rs8105807 | A | G | 0.00413886 | 0.0178028 |
| 8 | 8:23397266_T_C | 23397266 | 3E-05 | rs10503726 | T | C | 0.00350505 | -0.0147795 |
| 22 | 22:23490990_A_G | 23490990 | 3E-05 | rs2267006 | A | G | 0.00340503 | -0.0149869 |
| 22 | 22:28306607_T_C | 28306607 | 3E-05 | rs5762399 | T | C | 0.00336238 | -0.014488 |
| 2 | 2:95822447_A_G | 95822447 | 3.1E-05 | rs3112998 | A | G | 0.00333593 | -0.0141591 |
| 8 | 8:143481193_G_A | 143481193 | 3.1E-05 | rs68095309 | G | A | 0.00349109 | 0.0151744 |
| 12 | 12:50476158_G_T | 50476158 | 3.1E-05 | 12:50476158:G:T | G | T | 0.00524224 | 0.0222679 |
| 3 | 3:25625821_G_A | 25625821 | 3.2E-05 | rs9809535 | G | A | 0.00522174 | -0.0210772 |
| 11 | 11:69704736_G_A | 69704736 | 3.2E-05 | rs28456383 | G | A | 0.00424352 | 0.0175259 |
| 2 | 2:62761855_G_T | 62761855 | 3.3E-05 | 2:62761855:G:T | G | T | 0.00344403 | -0.0137048 |
| 9 | 9:36458378_G_A | 36458378 | 3.3E-05 | rs10972916 | G | A | 0.00859915 | -0.0361996 |
| 4 | 4:162099158_C_T | 162099158 | 3.4E-05 | rs68062673 | C | T | 0.00428952 | 0.0170817 |
| 5 | 5:96552919_T_C | 96552919 | 3.4E-05 | rs181810864 | T | C | 0.0117692 | 0.0451392 |
| 10 | 10:64689926_C_A | 64689926 | 3.4E-05 | rs185179272 | C | A | 0.012184 | 0.0505903 |
| 12 | 12:113425282_T_G | 113425282 | 3.4E-05 | rs1293766 | T | G | 0.00350183 | -0.0145649 |
| 2 | 2:135594699_C_T | 135594699 | 3.5E-05 | rs1954874 | C | T | 0.00338175 | 0.013513 |
| 3 | 3:70163806_A_G | 70163806 | 3.5E-05 | rs6549269 | A | G | 0.00417518 | -0.0164692 |
| 6 | 6:28152567_G_A | 28152567 | 3.5E-05 | rs2622315 | G | A | 0.00397228 | -0.0169639 |
| 9 | 9:103509787_C_T | 103509787 | 3.5E-05 | rs141229389 | C | T | 0.014672 | -0.0590891 |
| 22 | 22:20014152_A_G | 20014152 | 3.5E-05 | rs5748504 | A | G | 0.00373206 | 0.0161188 |
| 14 | 14:69272842_C_T | 69272842 | 3.6E-05 | rs59686841 | C | T | 0.00343035 | 0.0142676 |
| 15 | 15:50770254_A_G | 50770254 | 3.6E-05 | rs17696277 | A | G | 0.00417049 | 0.0184444 |
| 3 | 3:23753135_T_C | 23753135 | 3.7E-05 | rs6550784 | T | C | 0.00373831 | 0.0156043 |
| 4 | 4:22496542_T_C | 22496542 | 3.7E-05 | rs118023067 | T | C | 0.00681241 | 0.027151 |
| 8 | 8:57341785_G_A | 57341785 | 3.7E-05 | rs56344606 | G | A | 0.00376409 | -0.01489 |
| 16 | 16:81621422_A_G | 81621422 | 3.7E-05 | rs8047876 | A | G | 0.00390789 | 0.0160679 |
| 18 | 18:54291372_T_C | 54291372 | 3.7E-05 | rs140566570 | T | C | 0.00707197 | -0.0287639 |
| 1 | 1:153961784_G_A | 153961784 | 3.8E-05 | rs12563539 | G | A | 0.00403454 | -0.0172311 |
| 6 | 6:6975894_G_T | 6975894 | 3.8E-05 | rs2802414 | G | T | 0.007789 | -0.0303772 |
| 8 | 8:113540151_G_A | 113540151 | 3.8E-05 | rs80134738 | G | A | 0.0124629 | -0.0477754 |
| 10 | 10:102751706_G_A | 102751706 | 3.8E-05 | rs112309064 | G | A | 0.00334226 | 0.0137713 |
| 12 | 12:38614835_G_A | 38614835 | 3.8E-05 | rs7971425 | G | A | 0.00734485 | -0.0304585 |
| 13 | 13:22427160_T_C | 22427160 | 3.8E-05 | rs685926 | T | C | 0.00988934 | -0.0436291 |
| 3 | 3:69150577_G_A | 69150577 | 3.9E-05 | rs7621852 | G | A | 0.0059398 | -0.0252945 |
| 16 | 16:80769791_G_A | 80769791 | 3.9E-05 | rs78407176 | G | A | 0.00693753 | 0.0277024 |
| 17 | 17:61538148_G_A | 61538148 | 3.9E-05 | rs138190086 | G | A | 0.00851749 | 0.0352252 |
| 1 | 1:2130496_G_A | 2130496 | 4E-05 | rs117392468 | G | A | 0.0149169 | -0.0599405 |
| 13 | 13:81335537_C_T | 81335537 | 4E-05 | rs2759238 | C | T | 0.0041408 | -0.0158786 |
| 18 | 18:55787050_G_A | 55787050 | 4E-05 | rs4940643 | G | A | 0.00819399 | 0.0336775 |
| 20 | 20:52695621_A_C | 52695621 | 4E-05 | rs141891960 | A | C | 0.00858387 | 0.0350638 |
| 2 | 2:58849989_G_A | 58849989 | 4.1E-05 | rs6545695 | G | A | 0.00332709 | 0.0132859 |
| 2 | 2:145947417_G_A | 145947417 | 4.1E-05 | rs36032963 | G | A | 0.0034277 | 0.013929 |
| 6 | 6:114408902_C_T | 114408902 | 4.1E-05 | rs117566129 | C | T | 0.0144827 | 0.0582165 |
| 8 | 8:24613284_C_T | 24613284 | 4.1E-05 | rs190420388 | C | T | 0.0120527 | -0.0534398 |
| 12 | 12:20153206_G_A | 20153206 | 4.1E-05 | rs17369625 | G | A | 0.00551281 | -0.0225952 |
| 13 | 13:69557671_A_G | 69557671 | 4.1E-05 | rs9564513 | A | G | 0.00357948 | -0.0145736 |
| 15 | 15:51351145_G_T | 51351145 | 4.1E-05 | rs1438926 | G | T | 0.00415977 | 0.0176024 |
| 3 | 3:122885838_C_T | 122885838 | 4.2E-05 | rs113613295 | C | T | 0.00494612 | -0.0192707 |
| 7 | 7:71407469_G_A | 71407469 | 4.2E-05 | rs80352603 | G | A | 0.0155994 | -0.0654753 |
| 12 | 12:120563292_T_C | 120563292 | 4.2E-05 | rs80102891 | T | C | 0.00612904 | -0.0254079 |
| 17 | 17:78558411_T_C | 78558411 | 4.2E-05 | rs12949279 | T | C | 0.00350081 | 0.0147354 |
| 2 | 2:146346331_A_G | 146346331 | 4.3E-05 | rs16825228 | A | G | 0.00333749 | 0.0135951 |
| 9 | 9:7336443_A_G | 7336443 | 4.3E-05 | rs12347615 | A | G | 0.00789884 | 0.0311418 |
| 19 | 19:56600771_C_T | 56600771 | 4.3E-05 | rs75898725 | C | T | 0.0037924 | -0.0151995 |
| 10 | 10:103829735_T_C | 103829735 | 4.4E-05 | rs140675306 | T | C | 0.0142702 | -0.059039 |
| 13 | 13:32351162_A_C | 32351162 | 4.4E-05 | 13:32351162:A:C | A | C | 0.00381531 | -0.0149064 |
| 16 | 16:9844685_C_T | 9844685 | 4.4E-05 | rs1819823 | C | T | 0.00460227 | 0.0186216 |
| 19 | 19:14273641_A_G | 14273641 | 4.4E-05 | rs3745462 | A | G | 0.00589331 | 0.0252204 |
| 19 | 19:58168582_C_T | 58168582 | 4.4E-05 | rs60739395 | C | T | 0.0129194 | -0.0489855 |
| 3 | 3:158206114_C_T | 158206114 | 4.5E-05 | rs11710813 | C | T | 0.0050667 | -0.0205999 |
| 6 | 6:26872409_C_T | 26872409 | 4.5E-05 | rs9348734 | C | T | 0.00400398 | 0.0167137 |
| 10 | 10:62390646_G_A | 62390646 | 4.5E-05 | rs10821808 | G | A | 0.00336331 | 0.0134382 |
| 4 | 4:107552462_T_C | 107552462 | 4.7E-05 | rs2098849 | T | C | 0.00647863 | 0.0264104 |
| 12 | 12:46086071_C_T | 46086071 | 4.7E-05 | rs4768657 | C | T | 0.00335806 | 0.012582 |
| 3 | 3:148696043_A_G | 148696043 | 4.8E-05 | rs76484642 | A | G | 0.00851189 | -0.0334046 |
| 7 | 7:56280558_G_A | 56280558 | 4.8E-05 | rs698623 | G | A | 0.00338637 | 0.0137185 |
| 7 | 7:144738973_T_C | 144738973 | 4.8E-05 | rs1534132 | T | C | 0.00398604 | -0.0162965 |
| 14 | 14:99478363_C_A | 99478363 | 4.8E-05 | rs1492307 | C | A | 0.00380166 | 0.0147868 |
| 5 | 5:148049239_C_T | 148049239 | 4.9E-05 | rs201842022 | C | T | 0.00350911 | 0.0144779 |
| 10 | 10:123329429_T_G | 123329429 | 5E-05 | rs2981428 | T | G | 0.00337566 | 0.0135623 |
| 22 | 22:44944258_G_A | 44944258 | 5E-05 | rs138751208 | G | A | 0.0156044 | -0.0634664 |
| 5 | 5:5866070_A_G | 5866070 | 5.1E-05 | rs1174905 | A | G | 0.00379122 | 0.0144385 |
| 11 | 11:125467176_C_T | 125467176 | 5.1E-05 | rs76225995 | C | T | 0.0108944 | -0.0432437 |
| 18 | 18:53404986_C_T | 53404986 | 5.1E-05 | rs17527878 | C | T | 0.00333797 | 0.0133007 |
| 18 | 18:56063861_T_G | 56063861 | 5.1E-05 | rs145003704 | T | G | 0.0127684 | 0.0528447 |
| 3 | 3:144633932_T_C | 144633932 | 5.2E-05 | rs79813695 | T | C | 0.018481 | -0.0796188 |
| 11 | 11:65362041_A_G | 65362041 | 5.2E-05 | rs58448771 | A | G | 0.00646831 | 0.0267101 |
| 11 | 11:69970412_G_A | 69970412 | 5.2E-05 | 11:69970412:G:A | G | A | 0.0039379 | 0.0153414 |
| 19 | 19:16053106_T_C | 16053106 | 5.2E-05 | 19:16053106:T:C | T | C | 0.00518012 | -0.0212629 |
| 15 | 15:67441750_C_A | 67441750 | 5.3E-05 | rs72743461 | C | A | 0.00814744 | 0.0327185 |
| 2 | 2:73910138_T_C | 73910138 | 5.4E-05 | rs35421685 | T | C | 0.00384507 | 0.0150786 |
| 4 | 4:136618495_G_A | 136618495 | 5.4E-05 | rs13127387 | G | A | 0.00342718 | -0.0129868 |
| 9 | 9:102305818_T_C | 102305818 | 5.4E-05 | rs116925378 | T | C | 0.0104331 | -0.0418798 |
| 17 | 17:18289956_C_T | 18289956 | 5.4E-05 | rs7221741 | C | T | 0.00387828 | -0.0156024 |
| 1 | 1:235120039_C_T | 235120039 | 5.5E-05 | rs16844319 | C | T | 0.00566547 | -0.0225939 |
| 18 | 18:21220681_A_C | 21220681 | 5.6E-05 | rs1030525 | A | C | 0.00462333 | -0.0181142 |
| 9 | 9:28937034_T_C | 28937034 | 5.7E-05 | rs1930738 | T | C | 0.00369699 | -0.0151201 |
| 2 | 2:55500289_T_C | 55500289 | 5.8E-05 | 2:55500289:T:C | T | C | 0.00789227 | -0.0313498 |
| 3 | 3:113912270_G_A | 113912270 | 5.8E-05 | rs17605608 | G | A | 0.00400457 | -0.0166596 |
| 5 | 5:87968864_A_G | 87968864 | 5.8E-05 | rs6882046 | A | G | 0.00332701 | -0.0128716 |
| 10 | 10:63264872_C_T | 63264872 | 5.8E-05 | rs10994785 | C | T | 0.00331981 | 0.013071 |
| 10 | 10:85954817_C_T | 85954817 | 5.9E-05 | rs11592253 | C | T | 0.00362919 | -0.0138032 |
| 10 | 10:24580519_G_A | 24580519 | 6E-05 | rs139955364 | G | A | 0.0189218 | 0.0760498 |
| 5 | 5:156666163_C_T | 156666163 | 6.1E-05 | rs6895925 | C | T | 0.00343206 | 0.01381 |
| 5 | 5:173807972_A_G | 173807972 | 6.1E-05 | rs117196348 | A | G | 0.00886448 | 0.035084 |
| 12 | 12:12597882_C_A | 12597882 | 6.1E-05 | rs7973069 | C | A | 0.00801592 | -0.0318577 |
| 3 | 3:64206741_A_G | 64206741 | 6.2E-05 | rs74764095 | A | G | 0.00874635 | 0.0329539 |
| 9 | 9:20583373_T_C | 20583373 | 6.2E-05 | rs1831458 | T | C | 0.00415608 | 0.0165605 |
| 10 | 10:104825846_C_T | 104825846 | 6.2E-05 | rs74468868 | C | T | 0.0139232 | -0.0551793 |
| 10 | 10:127989115_T_C | 127989115 | 6.2E-05 | rs11597113 | T | C | 0.00375997 | -0.0149925 |
| 1 | 1:151512895_A_G | 151512895 | 6.3E-05 | rs3828054 | A | G | 0.00744201 | 0.0292338 |
| 16 | 16:88453010_G_A | 88453010 | 6.3E-05 | rs74036009 | G | A | 0.0073844 | -0.0278098 |
| 4 | 4:177420335_C_T | 177420335 | 6.4E-05 | rs309700 | C | T | 0.0033395 | 0.0132014 |
| 10 | 10:18654027_A_C | 18654027 | 6.4E-05 | rs11013860 | A | C | 0.003331 | -0.0128952 |
| 20 | 20:58243535_G_A | 58243535 | 6.4E-05 | rs6070900 | G | A | 0.00370029 | -0.0150267 |
| 1 | 1:210122787_A_G | 210122787 | 6.5E-05 | rs7518683 | A | G | 0.00493517 | 0.0191164 |
| 2 | 2:152039078_T_C | 152039078 | 6.5E-05 | rs16829715 | T | C | 0.00560085 | -0.0219449 |
| 3 | 3:46888198_T_C | 46888198 | 6.5E-05 | rs56387622 | T | C | 0.00459992 | -0.0191057 |
| 4 | 4:140187612_T_G | 140187612 | 6.6E-05 | rs116191353 | T | G | 0.0100277 | -0.0393732 |
| 7 | 7:21463041_T_C | 21463041 | 6.6E-05 | rs39299 | T | C | 0.00704686 | 0.0286097 |
| 11 | 11:95061176_C_T | 95061176 | 6.7E-05 | rs560489 | C | T | 0.00362908 | -0.0141392 |
| 3 | 3:14969197_G_A | 14969197 | 6.8E-05 | rs12491077 | G | A | 0.00464135 | 0.0182166 |
| 5 | 5:52210122_T_G | 52210122 | 6.8E-05 | rs16880493 | T | G | 0.00391708 | 0.016416 |
| 9 | 9:102584069_C_T | 102584069 | 6.8E-05 | rs151083947 | C | T | 0.0104499 | -0.0412478 |
| 10 | 10:90067305_G_A | 90067305 | 6.8E-05 | rs2312538 | G | A | 0.00393538 | 0.0157195 |
| 10 | 10:115824246_G_A | 115824246 | 6.8E-05 | rs80113039 | G | A | 0.010702 | 0.0391918 |
| 4 | 4:172034857_G_A | 172034857 | 6.9E-05 | rs7671063 | G | A | 0.00334003 | 0.0133908 |
| 7 | 7:150866852_T_C | 150866852 | 6.9E-05 | rs74827910 | T | C | 0.0136666 | -0.053117 |
| 10 | 10:60592816_C_T | 60592816 | 6.9E-05 | rs12783243 | C | T | 0.00441191 | 0.0174847 |
| 10 | 10:111023049_C_T | 111023049 | 6.9E-05 | rs7087451 | C | T | 0.00838634 | -0.0323076 |
| 17 | 17:26716917_G_T | 26716917 | 6.9E-05 | rs4795434 | G | T | 0.00391588 | 0.0148176 |
| 2 | 2:45501714_A_G | 45501714 | 7E-05 | rs187824475 | A | G | 0.0142823 | 0.0549671 |
| 4 | 4:7799619_A_G | 7799619 | 7E-05 | 4:7799619:A:G | A | G | 0.00488796 | 0.0206942 |
| 3 | 3:18408323_C_A | 18408323 | 7.1E-05 | rs34669921 | C | A | 0.00348749 | -0.0140216 |
| 1 | 1:164739573_A_G | 164739573 | 7.2E-05 | rs12736383 | A | G | 0.00399105 | 0.0155411 |
| 7 | 7:80286003_C_T | 80286003 | 7.2E-05 | rs75326924 | C | T | 0.00785365 | -0.0314952 |
| 9 | 9:23703442_G_A | 23703442 | 7.2E-05 | rs77133026 | G | A | 0.00943427 | 0.037784 |
| 7 | 7:114734388_C_T | 114734388 | 7.3E-05 | rs76216141 | C | T | 0.00707892 | 0.0279487 |
| 14 | 14:22051519_G_A | 22051519 | 7.4E-05 | rs17182599 | G | A | 0.00347184 | -0.013272 |
| 16 | 16:75328308_T_C | 75328308 | 7.4E-05 | rs11640018 | T | C | 0.00333854 | -0.0139976 |
| 2 | 2:225218490_G_A | 225218490 | 7.5E-05 | rs77987249 | G | A | 0.0125018 | 0.0486645 |
| 4 | 4:81140898_C_A | 81140898 | 7.5E-05 | rs2013945 | C | A | 0.00348423 | -0.0138056 |
| 12 | 12:59689469_T_C | 59689469 | 7.5E-05 | rs36011226 | T | C | 0.00363259 | 0.0136681 |
| 8 | 8:76917645_C_T | 76917645 | 7.6E-05 | rs80094549 | C | T | 0.0173604 | -0.0674515 |
| 9 | 9:103052671_G_A | 103052671 | 7.6E-05 | rs112505949 | G | A | 0.0135578 | -0.0547395 |
| 17 | 17:45190544_T_C | 45190544 | 7.6E-05 | 17:45190544:T:C | T | C | 0.0137697 | 0.0547952 |
| 21 | 21:40294024_A_G | 40294024 | 7.6E-05 | rs2836757 | A | G | 0.00337325 | -0.0131366 |
| 4 | 4:147888347_A_G | 147888347 | 7.7E-05 | rs57587608 | A | G | 0.00587549 | 0.0220699 |
| 6 | 6:23980526_C_T | 23980526 | 7.7E-05 | rs573580 | C | T | 0.00364694 | 0.0149487 |
| 6 | 6:43408105_C_T | 43408105 | 7.7E-05 | rs67861980 | C | T | 0.00529232 | 0.022135 |
| 6 | 6:170556797_G_A | 170556797 | 7.7E-05 | rs149236617 | G | A | 0.0150155 | 0.0568551 |
| 7 | 7:19052733_G_A | 19052733 | 7.7E-05 | rs57301765 | G | A | 0.0035238 | -0.014364 |
| 12 | 12:45484258_G_T | 45484258 | 7.7E-05 | rs78219485 | G | T | 0.0142716 | 0.0513706 |
| 12 | 12:111300010_G_A | 111300010 | 7.7E-05 | rs2283351 | G | A | 0.00387152 | 0.0151219 |
| 13 | 13:23166794_A_G | 23166794 | 7.7E-05 | rs4313676 | A | G | 0.00391226 | 0.0156051 |
| 22 | 22:28757950_C_T | 28757950 | 7.7E-05 | rs5752726 | C | T | 0.00339952 | -0.0134382 |
| 4 | 4:24182454_G_A | 24182454 | 7.9E-05 | rs16875252 | G | A | 0.0038644 | 0.0146237 |
| 4 | 4:115783993_T_C | 115783993 | 7.9E-05 | rs117265750 | T | C | 0.00901579 | -0.0341921 |
| 9 | 9:4298589_G_A | 4298589 | 8E-05 | rs35338539 | G | A | 0.0035222 | 0.0134259 |
| 1 | 1:64921341_A_G | 64921341 | 8.1E-05 | rs305541 | A | G | 0.00641935 | -0.0247843 |
| 5 | 5:141816615_A_C | 141816615 | 8.1E-05 | rs3853475 | A | C | 0.00339377 | 0.0135934 |
| 6 | 6:25292213_C_T | 25292213 | 8.1E-05 | rs6923529 | C | T | 0.00336619 | -0.0128098 |
| 4 | 4:21406883_G_A | 21406883 | 8.2E-05 | rs73104159 | G | A | 0.00564481 | 0.0228067 |
| 5 | 5:77917525_A_C | 77917525 | 8.2E-05 | rs6883793 | A | C | 0.00462541 | 0.0173144 |
| 10 | 10:123467204_A_G | 123467204 | 8.2E-05 | rs11200087 | A | G | 0.00333413 | 0.0127407 |
| 5 | 5:93909599_T_C | 93909599 | 8.3E-05 | rs6862592 | T | C | 0.00664593 | -0.0262878 |
| 20 | 20:56478627_G_A | 56478627 | 8.3E-05 | rs7261759 | G | A | 0.0108421 | 0.0428968 |
| 4 | 4:18465527_G_A | 18465527 | 8.5E-05 | rs139654041 | G | A | 0.0131431 | -0.0515435 |
| 5 | 5:52753814_A_G | 52753814 | 8.5E-05 | rs2042333 | A | G | 0.00338462 | -0.0135079 |
| 14 | 14:59678981_T_C | 59678981 | 8.6E-05 | rs17095879 | T | C | 0.00363537 | 0.0145631 |
| 18 | 18:833497_G_A | 833497 | 8.6E-05 | rs77849147 | G | A | 0.00392678 | -0.0151218 |
| 12 | 12:113629949_C_T | 113629949 | 8.7E-05 | rs1043845 | C | T | 0.00340312 | -0.0137214 |
| 3 | 3:30293682_T_C | 30293682 | 8.8E-05 | rs62242232 | T | C | 0.00351478 | -0.0140037 |
| 5 | 5:92346424_C_A | 92346424 | 8.8E-05 | rs345911 | C | A | 0.00355581 | -0.0136861 |
| 7 | 7:27089097_G_A | 27089097 | 8.9E-05 | rs200284884 | G | A | 0.0112198 | 0.0438627 |
| 20 | 20:2502533_G_A | 2502533 | 8.9E-05 | rs73892471 | G | A | 0.00656123 | -0.0260517 |
| 2 | 2:52306833_T_G | 52306833 | 9E-05 | rs970559441 | T | G | 0.013361 | -0.050735 |
| 2 | 2:55937887_G_A | 55937887 | 9E-05 | rs77924531 | G | A | 0.00682257 | 0.0265466 |
| 14 | 14:74219087_G_A | 74219087 | 9E-05 | rs62006080 | G | A | 0.00350601 | -0.0145813 |
| 1 | 1:100313077_C_T | 100313077 | 9.1E-05 | rs74102269 | C | T | 0.00636477 | 0.0251255 |
| 2 | 2:110263882_G_A | 110263882 | 9.1E-05 | rs76063523 | G | A | 0.0115222 | -0.0435401 |
| 8 | 8:76296131_A_G | 76296131 | 9.1E-05 | rs5005618 | A | G | 0.00334041 | -0.0133521 |
| 12 | 12:110981666_G_A | 110981666 | 9.1E-05 | rs141034698 | G | A | 0.00869614 | 0.0327307 |
| 6 | 6:111155400_T_G | 111155400 | 9.2E-05 | rs1279585 | T | G | 0.00444634 | -0.0185995 |
| 6 | 6:134144335_G_A | 134144335 | 9.2E-05 | rs35792945 | G | A | 0.0054855 | -0.0212943 |
| 1 | 1:244294710_G_T | 244294710 | 9.3E-05 | rs1578170 | G | T | 0.00333777 | 0.0120935 |
| 8 | 8:106062874_C_T | 106062874 | 9.3E-05 | rs285869 | C | T | 0.00344613 | 0.0140972 |
| 8 | 8:144130643_G_A | 144130643 | 9.3E-05 | rs78140118 | G | A | 0.0105659 | 0.0407438 |
| 17 | 17:18880268_C_A | 18880268 | 9.3E-05 | rs2472715 | C | A | 0.00347277 | 0.0134206 |
| 8 | 8:95635953_G_A | 95635953 | 9.5E-05 | rs3133629 | G | A | 0.00543772 | -0.0227098 |
| 11 | 11:47354787_C_T | 47354787 | 9.5E-05 | rs1052373 | C | T | 0.00361651 | 0.0140427 |
| 14 | 14:41844310_T_C | 41844310 | 9.5E-05 | rs72682814 | T | C | 0.00653254 | -0.0242484 |
| 2 | 2:85441399_A_G | 85441399 | 9.6E-05 | rs12619496 | A | G | 0.00350845 | -0.0141559 |
| 6 | 6:75916047_C_T | 75916047 | 9.6E-05 | 6:75916047:C:T | C | T | 0.0142977 | -0.0528437 |
| 9 | 9:90355814_T_C | 90355814 | 9.6E-05 | rs3128516 | T | C | 0.00372267 | -0.0143649 |
| 18 | 18:69137991_A_G | 69137991 | 9.6E-05 | rs150402334 | A | G | 0.0113846 | -0.0449915 |
| 3 | 3:29335426_A_G | 29335426 | 9.7E-05 | rs9878305 | A | G | 0.00411406 | -0.0158719 |
| 9 | 9:36984853_T_C | 36984853 | 9.7E-05 | rs7036385 | T | C | 0.00345367 | -0.012779 |
| 10 | 10:104438825_G_A | 104438825 | 9.7E-05 | rs146952142 | G | A | 0.0154392 | -0.0611044 |
| 1 | 1:10786459_G_A | 10786459 | 9.8E-05 | rs117615835 | G | A | 0.0140393 | 0.059922 |
| 2 | 2:31112621_T_G | 31112621 | 9.8E-05 | rs6733894 | T | G | 0.00824588 | -0.0311591 |
| 2 | 2:12405887_G_A | 12405887 | 9.9E-05 | rs1453496 | G | A | 0.00490093 | -0.0189843 |
| 4 | 4:147895803_A_G | 147895803 | 9.9E-05 | rs12650032 | A | G | 0.00339343 | 0.0125858 |
| 8 | 8:17419299_A_G | 17419299 | 9.9E-05 | rs2705062 | A | G | 0.00389862 | 0.0154608 |
| 1 | 1:7990367_G_A | 7990367 | 1E-04 | rs2131630 | G | A | 0.00332611 | -0.0127309 |
| 1 | 1:25409256_A_G | 25409256 | 1E-04 | rs12077761 | A | G | 0.0038038 | 0.0148979 |
| 2 | 2:111754548_T_C | 111754548 | 1E-04 | rs6760171 | T | C | 0.00344039 | 0.0135039 |
| 3 | 3:23590534_A_C | 23590534 | 1E-04 | rs73045615 | A | C | 0.00358844 | -0.0141191 |
| 3 | 3:54394121_C_A | 54394121 | 1E-04 | rs118076291 | C | A | 0.00829121 | -0.0318466 |
| 4 | 4:107251278_C_T | 107251278 | 1E-04 | rs74285705 | C | T | 0.00408637 | -0.0166633 |
| 6 | 6:35719327_C_T | 35719327 | 1E-04 | rs2766554 | C | T | 0.0035768 | 0.0140348 |
| 6 | 6:156772726_G_T | 156772726 | 1E-04 | rs117987651 | G | T | 0.0092927 | -0.0386212 |
| 7 | 7:1729633_C_T | 1729633 | 1E-04 | rs117122841 | C | T | 0.00994474 | -0.0367916 |
| 12 | 12:71585743_T_C | 71585743 | 1E-04 | 12:71585743:T:C | T | C | 0.00388928 | 0.0158554 |
| 12 | 12:110157492_A_G | 110157492 | 1E-04 | rs56848810 | A | G | 0.00403722 | -0.0153459 |
| 12 | 12:112993744_C_A | 112993744 | 1E-04 | rs76828983 | C | A | 0.0125192 | -0.0492516 |
| 12 | 12:120769657_G_A | 120769657 | 1E-04 | rs2701632 | G | A | 0.00351154 | -0.0130537 |
| 17 | 17:42311513_G_A | 42311513 | 1E-04 | rs76876339 | G | A | 0.00551094 | 0.0216639 |
| 18 | 18:69342915_A_G | 69342915 | 1E-04 | rs1942399 | A | G | 0.00386257 | -0.0142875 |
| 19 | 19:31778409_C_T | 31778409 | 1E-04 | rs35435920 | C | T | 0.00359053 | -0.013532 |
| 1 | 1:40714010_G_A | 40714010 | 0.00011 | rs12126903 | G | A | 0.00405268 | -0.0160003 |
| 2 | 2:45536897_C_T | 45536897 | 0.00011 | rs17393058 | C | T | 0.0108001 | 0.0423096 |
| 2 | 2:54016456_G_A | 54016456 | 0.00011 | rs186332641 | G | A | 0.0106518 | -0.0396383 |
| 2 | 2:97837911_C_T | 97837911 | 0.00011 | rs2924021 | C | T | 0.00344191 | -0.0128252 |
| 2 | 2:241842176_C_T | 241842176 | 0.00011 | 2:241842176:C:T | C | T | 0.00544396 | 0.0213112 |
| 2 | 2:242612909_A_G | 242612909 | 0.00011 | rs1130906 | A | G | 0.00365472 | -0.0151345 |
| 3 | 3:111150531_G_A | 111150531 | 0.00011 | rs138562480 | G | A | 0.0131615 | -0.0529445 |
| 3 | 3:148476977_A_G | 148476977 | 0.00011 | rs1492080 | A | G | 0.00377652 | 0.0145782 |
| 3 | 3:163494059_G_A | 163494059 | 0.00011 | rs16847256 | G | A | 0.00342296 | -0.0129833 |
| 4 | 4:3020584_C_T | 3020584 | 0.00011 | rs111683622 | C | T | 0.00543292 | 0.0201898 |
| 4 | 4:63921664_G_A | 63921664 | 0.00011 | 4:63921664:G:A | G | A | 0.0130649 | 0.0494888 |
| 4 | 4:102108914_A_G | 102108914 | 0.00011 | rs2850988 | A | G | 0.00353473 | -0.0129985 |
| 5 | 5:52193518_A_C | 52193518 | 0.00011 | rs17829997 | A | C | 0.00341033 | 0.0123784 |
| 5 | 5:87582787_G_A | 87582787 | 0.00011 | rs17481705 | G | A | 0.0130736 | 0.0479087 |
| 5 | 5:156742678_T_C | 156742678 | 0.00011 | rs62389529 | T | C | 0.016577 | -0.0647544 |
| 6 | 6:40394175_T_C | 40394175 | 0.00011 | rs12173741 | T | C | 0.00340079 | -0.0125854 |
| 6 | 6:106976109_G_T | 106976109 | 0.00011 | rs191367313 | G | T | 0.0155984 | 0.0604964 |
| 7 | 7:138627696_G_A | 138627696 | 0.00011 | rs12707403 | G | A | 0.00525914 | -0.0194775 |
| 10 | 10:8696718_A_C | 8696718 | 0.00011 | rs4749802 | A | C | 0.00363125 | -0.0138036 |
| 10 | 10:29875489_A_G | 29875489 | 0.00011 | rs4749466 | A | G | 0.00497194 | 0.0187066 |
| 11 | 11:16231935_A_C | 16231935 | 0.00011 | rs11023871 | A | C | 0.00422085 | -0.0162137 |
| 11 | 11:47752775_A_G | 47752775 | 0.00011 | rs17788930 | A | G | 0.00359568 | -0.0139365 |
| 11 | 11:61496657_C_T | 61496657 | 0.00011 | rs198417 | C | T | 0.00352137 | -0.0145533 |
| 12 | 12:111275201_A_G | 111275201 | 0.00011 | rs4766431 | A | G | 0.0034644 | -0.0133547 |
| 13 | 13:89816397_T_C | 89816397 | 0.00011 | rs9522449 | T | C | 0.00421021 | -0.0162735 |
| 14 | 14:21148504_C_T | 21148504 | 0.00011 | rs79624797 | C | T | 0.00452136 | -0.0178162 |
| 14 | 14:31479291_T_C | 31479291 | 0.00011 | rs179715 | T | C | 0.00335381 | -0.0123414 |
| 15 | 15:85446666_G_A | 85446666 | 0.00011 | rs12913378 | G | A | 0.00698885 | -0.0261208 |
| 17 | 17:46476346_G_A | 46476346 | 0.00011 | rs562259366 | G | A | 0.0149723 | 0.0614126 |
| 20 | 20:14154051_T_C | 14154051 | 0.00011 | rs192691984 | T | C | 0.01443 | 0.0523823 |
| 1 | 1:1960388_C_T | 1960388 | 0.00012 | rs74802917 | C | T | 0.00787691 | 0.028315 |
| 1 | 1:100383613_A_G | 100383613 | 0.00012 | rs117596745 | A | G | 0.00932415 | -0.0358418 |
| 1 | 1:177852580_T_C | 177852580 | 0.00012 | rs633715 | T | C | 0.00398177 | -0.0143843 |
| 1 | 1:220350751_C_T | 220350751 | 0.00012 | rs2577127 | C | T | 0.00668629 | 0.0263573 |
| 1 | 1:239132857_A_G | 239132857 | 0.00012 | rs59107905 | A | G | 0.00773689 | -0.0277679 |
| 2 | 2:28650506_G_A | 28650506 | 0.00012 | rs6731639 | G | A | 0.00471663 | 0.018792 |
| 2 | 2:79687252_A_C | 79687252 | 0.00012 | 2:79687252:A:C | A | C | 0.00331986 | 0.0131049 |
| 3 | 3:114778005_C_T | 114778005 | 0.00012 | 3:114778005:C:T | C | T | 0.00371459 | -0.0136189 |
| 4 | 4:115923950_A_C | 115923950 | 0.00012 | rs2620414 | A | C | 0.00336361 | 0.0129731 |
| 4 | 4:185925922_A_C | 185925922 | 0.00012 | rs11132270 | A | C | 0.00377266 | 0.0139751 |
| 5 | 5:32811218_A_G | 32811218 | 0.00012 | rs74686705 | A | G | 0.0169032 | 0.0644186 |
| 5 | 5:76158670_A_G | 76158670 | 0.00012 | rs78562330 | A | G | 0.0146456 | 0.0527626 |
| 6 | 6:55234368_G_A | 55234368 | 0.00012 | rs937637 | G | A | 0.012745 | 0.0500014 |
| 9 | 9:34188563_T_C | 34188563 | 0.00012 | rs10814077 | T | C | 0.00568704 | 0.0221076 |
| 10 | 10:7206424_C_T | 7206424 | 0.00012 | rs10905113 | C | T | 0.0034084 | 0.0133865 |
| 10 | 10:123347875_C_T | 123347875 | 0.00012 | rs1219644 | C | T | 0.00617721 | -0.0239477 |
| 12 | 12:66580735_G_A | 66580735 | 0.00012 | rs11176082 | G | A | 0.0035501 | -0.0139534 |
| 13 | 13:22496925_C_T | 22496925 | 0.00012 | rs74381890 | C | T | 0.00935882 | 0.0375106 |
| 14 | 14:80306299_C_T | 80306299 | 0.00012 | rs72698455 | C | T | 0.00463881 | -0.0177644 |
| 16 | 16:71434237_C_T | 71434237 | 0.00012 | rs78594369 | C | T | 0.00675208 | -0.0253764 |
| 17 | 17:46475894_C_T | 46475894 | 0.00012 | rs142350674 | C | T | 0.016349 | 0.0648788 |
| 18 | 18:45923445_G_T | 45923445 | 0.00012 | 18:45923445:G:T | G | T | 0.00929356 | -0.0364614 |
| 19 | 19:13757663_G_A | 13757663 | 0.00012 | rs12461326 | G | A | 0.00459653 | 0.0183399 |
| 20 | 20:40588547_C_T | 40588547 | 0.00012 | rs73261133 | C | T | 0.00579197 | -0.0226209 |
| 2 | 2:170204800_G_T | 170204800 | 0.00013 | 2:170204800:G:T | G | T | 0.00336085 | -0.0133414 |
| 2 | 2:176503622_G_A | 176503622 | 0.00013 | rs60439491 | G | A | 0.00404403 | 0.0151076 |
| 2 | 2:224964048_A_G | 224964048 | 0.00013 | rs2006944 | A | G | 0.00352164 | 0.0132804 |
| 3 | 3:23715184_C_T | 23715184 | 0.00013 | rs4591453 | C | T | 0.00411722 | 0.0152521 |
| 3 | 3:47338554_C_T | 47338554 | 0.00013 | rs11713101 | C | T | 0.00366563 | -0.014161 |
| 3 | 3:86036309_A_C | 86036309 | 0.00013 | rs56339824 | A | C | 0.00334501 | 0.0126256 |
| 6 | 6:47362439_C_T | 47362439 | 0.00013 | rs6934355 | C | T | 0.0040299 | -0.0159861 |
| 6 | 6:122229646_G_A | 122229646 | 0.00013 | rs373665414 | G | A | 0.0176994 | 0.0659155 |
| 7 | 7:11893275_G_A | 11893275 | 0.00013 | rs12234369 | G | A | 0.00578951 | 0.0222535 |
| 7 | 7:28652485_G_T | 28652485 | 0.00013 | rs34589594 | G | T | 0.00377495 | 0.0135259 |
| 8 | 8:59669002_C_T | 59669002 | 0.00013 | 8:59669002:C:T | C | T | 0.00454548 | 0.0167781 |
| 8 | 8:140093334_C_T | 140093334 | 0.00013 | rs73365921 | C | T | 0.0164442 | 0.0628285 |
| 8 | 8:143887054_A_G | 143887054 | 0.00013 | rs13255523 | A | G | 0.00364859 | 0.0140864 |
| 10 | 10:83606191_G_A | 83606191 | 0.00013 | rs11191528 | G | A | 0.00406917 | -0.0161475 |
| 11 | 11:44929533_G_A | 44929533 | 0.00013 | rs77837721 | G | A | 0.0135743 | -0.050601 |
| 12 | 12:67835821_C_T | 67835821 | 0.00013 | rs35583684 | C | T | 0.00446175 | 0.0172432 |
| 12 | 12:111738364_A_G | 111738364 | 0.00013 | rs12231269 | A | G | 0.00465031 | -0.0168025 |
| 12 | 12:113386343_G_T | 113386343 | 0.00013 | rs141701964 | G | T | 0.0143948 | 0.0542947 |
| 21 | 21:20802015_T_C | 20802015 | 0.00013 | rs117070430 | T | C | 0.00738991 | 0.028317 |
| 22 | 22:19975444_A_G | 19975444 | 0.00013 | rs2238786 | A | G | 0.00423322 | -0.0160222 |
| 22 | 22:29008417_T_C | 29008417 | 0.00013 | rs2224337 | T | C | 0.00344117 | -0.013064 |
| 1 | 1:111067762_T_C | 111067762 | 0.00014 | rs72976916 | T | C | 0.00437152 | -0.0163044 |
| 1 | 1:174988945_A_G | 174988945 | 0.00014 | rs150588877 | A | G | 0.0133204 | -0.0523661 |
| 2 | 2:97564284_G_A | 97564284 | 0.00014 | rs2314650 | G | A | 0.00338139 | 0.0122517 |
| 3 | 3:47950859_G_A | 47950859 | 0.00014 | rs140205036 | G | A | 0.0105585 | -0.039928 |
| 3 | 3:111439158_C_T | 111439158 | 0.00014 | rs2399394 | C | T | 0.00355 | 0.0126219 |
| 5 | 5:66776884_A_G | 66776884 | 0.00014 | rs6869587 | A | G | 0.00817783 | -0.0320429 |
| 5 | 5:122755334_G_A | 122755334 | 0.00014 | rs12719311 | G | A | 0.00335564 | -0.012047 |
| 7 | 7:77593973_C_T | 77593973 | 0.00014 | rs1211911 | C | T | 0.00346658 | -0.0137814 |
| 8 | 8:135506830_C_T | 135506830 | 0.00014 | rs11776162 | C | T | 0.00581797 | -0.0223111 |
| 9 | 9:38659593_A_G | 38659593 | 0.00014 | rs112109946 | A | G | 0.00964569 | -0.0360886 |
| 12 | 12:78929753_C_A | 78929753 | 0.00014 | rs187435279 | C | A | 0.016667 | -0.0619463 |
| 12 | 12:79821286_A_C | 79821286 | 0.00014 | rs12228474 | A | C | 0.00402437 | -0.0161272 |
| 13 | 13:99170520_G_A | 99170520 | 0.00014 | rs4772093 | G | A | 0.00388 | -0.0150598 |
| 1 | 1:110470592_G_A | 110470592 | 0.00015 | rs333948 | G | A | 0.00347284 | 0.0134169 |
| 4 | 4:137788289_G_T | 137788289 | 0.00015 | rs72726382 | G | T | 0.00462192 | -0.0182425 |
| 5 | 5:43551103_T_C | 43551103 | 0.00015 | rs145205073 | T | C | 0.00738052 | 0.0274657 |
| 5 | 5:57591153_T_C | 57591153 | 0.00015 | rs37490 | T | C | 0.00546032 | -0.0218627 |
| 6 | 6:39971284_C_T | 39971284 | 0.00015 | rs2092863 | C | T | 0.00344259 | 0.0135981 |
| 6 | 6:98157997_C_T | 98157997 | 0.00015 | 6:98157997:C:T | C | T | 0.00783517 | 0.029932 |
| 7 | 7:29103222_T_C | 29103222 | 0.00015 | rs695069 | T | C | 0.00356767 | -0.0131851 |
| 8 | 8:110136041_G_A | 110136041 | 0.00015 | rs12543246 | G | A | 0.00334493 | 0.0124503 |
| 9 | 9:135225120_T_C | 135225120 | 0.00015 | rs56204293 | T | C | 0.0136595 | -0.052441 |
| 10 | 10:67847971_T_C | 67847971 | 0.00015 | rs188241366 | T | C | 0.0129673 | -0.0496318 |
| 11 | 11:112843006_A_G | 112843006 | 0.00015 | rs11214440 | A | G | 0.00408781 | 0.0154475 |
| 13 | 13:109595090_A_G | 109595090 | 0.00015 | rs78813813 | A | G | 0.0113491 | -0.0401809 |
| 17 | 17:18918740_G_T | 18918740 | 0.00015 | rs75957782 | G | T | 0.00441661 | 0.0168254 |
| 1 | 1:28461661_T_C | 28461661 | 0.00016 | rs10751727 | T | C | 0.00336896 | 0.0130798 |
| 1 | 1:177843588_A_C | 177843588 | 0.00016 | rs2149184 | A | C | 0.00426089 | -0.015518 |
| 2 | 2:65358329_G_T | 65358329 | 0.00016 | rs2422437 | G | T | 0.00373592 | -0.0146772 |
| 4 | 4:111399119_G_A | 111399119 | 0.00016 | rs7670649 | G | A | 0.00384622 | 0.0148188 |
| 4 | 4:156642884_A_G | 156642884 | 0.00016 | rs3796581 | A | G | 0.00393987 | 0.0148607 |
| 5 | 5:122027308_A_G | 122027308 | 0.00016 | rs6891051 | A | G | 0.00493067 | 0.0181544 |
| 5 | 5:178570631_G_A | 178570631 | 0.00016 | rs10041147 | G | A | 0.00382561 | 0.0143941 |
| 6 | 6:7920361_A_G | 7920361 | 0.00016 | rs1594467 | A | G | 0.00849687 | 0.0316886 |
| 6 | 6:31566204_C_T | 31566204 | 0.00016 | rs2509217 | C | T | 0.00453989 | 0.0161312 |
| 7 | 7:34952750_G_A | 34952750 | 0.00016 | rs4098875 | G | A | 0.003412 | -0.0127406 |
| 9 | 9:128766237_A_C | 128766237 | 0.00016 | rs201113307 | A | C | 0.0157691 | 0.0579465 |
| 9 | 9:138327718_T_C | 138327718 | 0.00016 | rs11103005 | T | C | 0.00685959 | 0.0250583 |
| 10 | 10:4529068_C_T | 4529068 | 0.00016 | rs4584478 | C | T | 0.0042352 | -0.0155816 |
| 10 | 10:85303891_C_A | 85303891 | 0.00016 | rs150840421 | C | A | 0.0119352 | 0.0453139 |
| 11 | 11:3856553_C_T | 3856553 | 0.00016 | rs1451722 | C | T | 0.00332554 | -0.0125663 |
| 11 | 11:4176502_A_C | 4176502 | 0.00016 | rs9919651 | A | C | 0.00420523 | -0.0160743 |
| 14 | 14:35885747_G_A | 35885747 | 0.00016 | rs8018193 | G | A | 0.00343293 | -0.0123361 |
| 16 | 16:74491733_A_G | 74491733 | 0.00016 | rs2288054 | A | G | 0.00353408 | 0.0132313 |
| 17 | 17:57970861_C_T | 57970861 | 0.00016 | rs561841596 | C | T | 0.0126048 | 0.0477104 |
| 20 | 20:31297904_G_A | 31297904 | 0.00016 | rs150232961 | G | A | 0.0177063 | 0.0670514 |
| 21 | 21:22585244_G_A | 22585244 | 0.00016 | rs143980246 | G | A | 0.00715333 | -0.0270232 |
| 2 | 2:110382217_C_A | 110382217 | 0.00017 | rs919942 | C | A | 0.0040008 | 0.01479 |
| 2 | 2:136150855_A_G | 136150855 | 0.00017 | rs79762714 | A | G | 0.0105746 | 0.0379496 |
| 2 | 2:240091511_C_T | 240091511 | 0.00017 | rs3791527 | C | T | 0.00340893 | 0.0119863 |
| 4 | 4:59463082_A_G | 59463082 | 0.00017 | rs190750 | A | G | 0.00350328 | -0.0127652 |
| 4 | 4:121267902_C_T | 121267902 | 0.00017 | rs75306709 | C | T | 0.00435342 | 0.0157784 |
| 5 | 5:4689307_G_A | 4689307 | 0.00017 | rs6869481 | G | A | 0.00345761 | -0.0134534 |
| 6 | 6:137272447_C_T | 137272447 | 0.00017 | rs9389453 | C | T | 0.00343629 | -0.0126582 |
| 7 | 7:40329041_A_G | 40329041 | 0.00017 | rs117841416 | A | G | 0.013623 | 0.0510397 |
| 7 | 7:151415041_A_G | 151415041 | 0.00017 | rs10224002 | A | G | 0.00801122 | -0.0286446 |
| 8 | 8:9650401_T_C | 9650401 | 0.00017 | rs13249234 | T | C | 0.00372622 | 0.0137457 |
| 8 | 8:23074112_G_A | 23074112 | 0.00017 | rs146863456 | G | A | 0.0150828 | -0.060165 |
| 8 | 8:82498419_T_C | 82498419 | 0.00017 | rs57228086 | T | C | 0.00338432 | 0.0121395 |
| 9 | 9:91054373_G_A | 91054373 | 0.00017 | rs117087909 | G | A | 0.014268 | -0.0522238 |
| 10 | 10:63518831_T_C | 63518831 | 0.00017 | rs12255851 | T | C | 0.0033988 | 0.0126508 |
| 10 | 10:89836250_A_G | 89836250 | 0.00017 | rs17108310 | A | G | 0.0124433 | 0.0475938 |
| 11 | 11:48112468_C_T | 48112468 | 0.00017 | 11:48112468:C:T | C | T | 0.00428004 | -0.0156369 |
| 12 | 12:113161828_C_T | 113161828 | 0.00017 | rs78678577 | C | T | 0.00531453 | -0.0196943 |
| 12 | 12:115495279_G_A | 115495279 | 0.00017 | rs10850434 | G | A | 0.00337464 | -0.012711 |
| 14 | 14:34531884_T_C | 34531884 | 0.00017 | rs79269585 | T | C | 0.00472081 | 0.018104 |
| 21 | 21:21723700_A_G | 21723700 | 0.00017 | rs76480463 | A | G | 0.00644746 | 0.0240273 |
| 21 | 21:29922390_G_T | 29922390 | 0.00017 | rs977689 | G | T | 0.00490075 | -0.0166978 |
| 1 | 1:156434703_G_T | 156434703 | 0.00018 | rs1050316 | G | T | 0.00375072 | 0.0133782 |
| 1 | 1:201634354_A_G | 201634354 | 0.00018 | rs115331085 | A | G | 0.0140317 | 0.0529357 |
| 1 | 1:214425094_T_C | 214425094 | 0.00018 | rs199810932 | T | C | 0.0132869 | -0.0514823 |
| 1 | 1:234744250_G_A | 234744250 | 0.00018 | 1:234744250:G:A | G | A | 0.00384874 | -0.0148754 |
| 2 | 2:18886374_C_T | 18886374 | 0.00018 | rs34811804 | C | T | 0.00440641 | 0.0158323 |
| 2 | 2:97487699_A_C | 97487699 | 0.00018 | rs62152795 | A | C | 0.00488189 | -0.0179789 |
| 2 | 2:125486642_G_A | 125486642 | 0.00018 | rs77666577 | G | A | 0.00835631 | 0.0325372 |
| 2 | 2:150482211_A_G | 150482211 | 0.00018 | rs16827266 | A | G | 0.00339014 | -0.0124081 |
| 3 | 3:16478476_T_C | 16478476 | 0.00018 | rs73043160 | T | C | 0.00691471 | 0.0275733 |
| 3 | 3:156392857_C_T | 156392857 | 0.00018 | 3:156392857:C:T | C | T | 0.00764884 | -0.0295368 |
| 5 | 5:34777838_C_T | 34777838 | 0.00018 | rs74799269 | C | T | 0.0075575 | 0.0284844 |
| 5 | 5:96445702_G_A | 96445702 | 0.00018 | rs316182 | G | A | 0.00393069 | -0.0154892 |
| 6 | 6:30823630_T_C | 30823630 | 0.00018 | rs9295928 | T | C | 0.00333065 | 0.0122995 |
| 6 | 6:33399778_C_T | 33399778 | 0.00018 | rs9394145 | C | T | 0.00400012 | 0.015484 |
| 6 | 6:43767703_C_T | 43767703 | 0.00018 | rs10948095 | C | T | 0.00347028 | -0.013019 |
| 7 | 7:155661522_C_T | 155661522 | 0.00018 | rs11772375 | C | T | 0.00361149 | 0.0140418 |
| 10 | 10:64588209_C_T | 64588209 | 0.00018 | rs1040875 | C | T | 0.00402724 | 0.0148725 |
| 11 | 11:73993943_A_G | 73993943 | 0.00018 | rs17132902 | A | G | 0.0112578 | 0.0441461 |
| 13 | 13:41675867_G_A | 41675867 | 0.00018 | rs9566696 | G | A | 0.00355666 | -0.0128797 |
| 16 | 16:79273993_G_A | 79273993 | 0.00018 | rs143346043 | G | A | 0.0142742 | -0.0554575 |
| 19 | 19:2149000_G_A | 2149000 | 0.00018 | rs1106861 | G | A | 0.00578822 | -0.0214497 |
| 1 | 1:56559352_A_G | 56559352 | 0.00019 | rs144128661 | A | G | 0.0173742 | 0.0631988 |
| 1 | 1:199235065_C_T | 199235065 | 0.00019 | rs10919651 | C | T | 0.0130605 | 0.0484508 |
| 1 | 1:230775069_C_A | 230775069 | 0.00019 | rs553452621 | C | A | 0.0121283 | -0.0472405 |
| 2 | 2:104824430_A_C | 104824430 | 0.00019 | rs74683777 | A | C | 0.0044534 | -0.0166766 |
| 2 | 2:107254158_G_A | 107254158 | 0.00019 | rs34386318 | G | A | 0.00337097 | 0.0129799 |
| 3 | 3:116342782_T_C | 116342782 | 0.00019 | rs79370904 | T | C | 0.0133257 | -0.0519494 |
| 4 | 4:44827368_A_G | 44827368 | 0.00019 | rs35765022 | A | G | 0.00434008 | -0.0156319 |
| 4 | 4:162309814_C_T | 162309814 | 0.00019 | rs10213523 | C | T | 0.00433514 | -0.0156427 |
| 4 | 4:175970410_T_C | 175970410 | 0.00019 | rs138553397 | T | C | 0.0132427 | -0.0473328 |
| 5 | 5:155606835_T_C | 155606835 | 0.00019 | rs68120551 | T | C | 0.00338648 | 0.012478 |
| 5 | 5:161703415_T_C | 161703415 | 0.00019 | rs182418090 | T | C | 0.018913 | -0.0688276 |
| 6 | 6:31068525_A_C | 31068525 | 0.00019 | 6:31068525:A:C | A | C | 0.00760143 | 0.0283684 |
| 6 | 6:101884091_A_G | 101884091 | 0.00019 | rs11156075 | A | G | 0.00345899 | 0.011786 |
| 6 | 6:114122669_G_A | 114122669 | 0.00019 | rs143069745 | G | A | 0.00935344 | 0.0342417 |
| 6 | 6:126191733_T_C | 126191733 | 0.00019 | rs2065168 | T | C | 0.00449026 | -0.0165708 |
| 6 | 6:153180343_C_T | 153180343 | 0.00019 | rs9371640 | C | T | 0.00351978 | 0.0136171 |
| 11 | 11:23211700_C_A | 23211700 | 0.00019 | rs10833962 | C | A | 0.00344366 | 0.0128802 |
| 12 | 12:73232402_A_C | 73232402 | 0.00019 | rs7955353 | A | C | 0.00369701 | 0.0132491 |
| 19 | 19:38797200_C_T | 38797200 | 0.00019 | rs190074681 | C | T | 0.0159324 | -0.0580203 |
| 20 | 20:21471354_C_A | 21471354 | 0.00019 | rs10854249 | C | A | 0.00337475 | -0.0125293 |
| 1 | 1:174156492_A_G | 174156492 | 2E-04 | rs148441585 | A | G | 0.0107713 | -0.0413565 |
| 1 | 1:183940527_T_C | 183940527 | 2E-04 | rs72637288 | T | C | 0.00341694 | -0.0126762 |
| 2 | 2:694063_G_A | 694063 | 2E-04 | rs11127496 | G | A | 0.00360346 | -0.0138325 |
| 2 | 2:183225686_G_A | 183225686 | 2E-04 | rs1564543 | G | A | 0.00337862 | 0.0129548 |
| 2 | 2:224841784_G_A | 224841784 | 2E-04 | rs12469511 | G | A | 0.00352971 | 0.0128038 |
| 3 | 3:34690149_A_G | 34690149 | 2E-04 | rs138313835 | A | G | 0.0128667 | 0.0481932 |
| 3 | 3:169572621_G_T | 169572621 | 2E-04 | rs3732452 | G | T | 0.00659949 | -0.0255003 |
| 4 | 4:81786809_A_G | 81786809 | 2E-04 | rs928440258 | A | G | 0.0162533 | 0.0616815 |
| 5 | 5:75619503_C_T | 75619503 | 2E-04 | rs142258686 | C | T | 0.0142454 | -0.0545443 |
| 6 | 6:115941394_A_G | 115941394 | 2E-04 | rs140481754 | A | G | 0.0122189 | 0.0451881 |
| 7 | 7:65776127_G_T | 65776127 | 2E-04 | rs12671152 | G | T | 0.00420891 | -0.0156471 |
| 8 | 8:53526364_C_T | 53526364 | 2E-04 | rs4873669 | C | T | 0.00421837 | 0.0152286 |
| 11 | 11:110306329_T_C | 110306329 | 2E-04 | rs10891105 | T | C | 0.00346386 | -0.0128434 |
| 12 | 12:111976776_C_T | 111976776 | 2E-04 | rs648997 | C | T | 0.00640737 | 0.0226111 |
| 16 | 16:79236908_T_G | 79236908 | 2E-04 | rs369487 | T | G | 0.00489554 | 0.0192845 |
| 22 | 22:33528630_A_G | 33528630 | 2E-04 | rs5998745 | A | G | 0.00369545 | -0.0136756 |
| 1 | 1:73282691_G_T | 73282691 | 0.00021 | rs1949888 | G | T | 0.00420131 | -0.0156753 |
| 1 | 1:241901524_G_T | 241901524 | 0.00021 | rs144053142 | G | T | 0.0158897 | -0.0558024 |
| 3 | 3:68734794_G_A | 68734794 | 0.00021 | rs2202338 | G | A | 0.0106136 | 0.0401125 |
| 4 | 4:87906997_G_T | 87906997 | 0.00021 | rs58822039 | G | T | 0.0033641 | -0.0128165 |
| 5 | 5:105400023_G_A | 105400023 | 0.00021 | rs181749999 | G | A | 0.0104901 | 0.0370693 |
| 6 | 6:70915130_C_T | 70915130 | 0.00021 | rs2176355 | C | T | 0.00422606 | 0.0161207 |
| 6 | 6:152915796_C_T | 152915796 | 0.00021 | rs818452 | C | T | 0.00496074 | 0.0181255 |
| 7 | 7:7840553_G_A | 7840553 | 0.00021 | rs4720754 | G | A | 0.00357549 | -0.0131968 |
| 9 | 9:90061444_G_A | 90061444 | 0.00021 | rs148422585 | G | A | 0.0102023 | 0.038715 |
| 10 | 10:21180887_A_G | 21180887 | 0.00021 | rs788963 | A | G | 0.00508269 | 0.0185538 |
| 12 | 12:133046535_G_A | 133046535 | 0.00021 | rs75256190 | G | A | 0.00385302 | 0.0151806 |
| 15 | 15:26102117_A_G | 26102117 | 0.00021 | rs11631375 | A | G | 0.00357213 | 0.0138864 |
| 17 | 17:1278700_G_A | 1278700 | 0.00021 | rs4790082 | G | A | 0.00333457 | -0.0123331 |
| 17 | 17:40260527_G_A | 40260527 | 0.00021 | rs550563651 | G | A | 0.0140047 | 0.0511754 |
| 17 | 17:55267188_G_A | 55267188 | 0.00021 | rs11651981 | G | A | 0.00584437 | -0.0214094 |
| 17 | 17:58895325_C_T | 58895325 | 0.00021 | rs77260877 | C | T | 0.00486567 | -0.0180385 |
| 20 | 20:39276408_A_C | 39276408 | 0.00021 | rs6129654 | A | C | 0.0144669 | 0.0553552 |
| 20 | 20:59910848_A_G | 59910848 | 0.00021 | rs8122914 | A | G | 0.00343511 | 0.0116214 |
| 1 | 1:230841509_A_G | 230841509 | 0.00022 | rs2478523 | A | G | 0.00332871 | -0.0125525 |
| 2 | 2:100113720_G_A | 100113720 | 0.00022 | rs13431410 | G | A | 0.00856034 | -0.0311814 |
| 2 | 2:161760489_C_T | 161760489 | 0.00022 | 2:161760489:C:T | C | T | 0.00906454 | -0.0330874 |
| 2 | 2:182993609_A_G | 182993609 | 0.00022 | rs1921151 | A | G | 0.00387845 | 0.0140288 |
| 3 | 3:23280826_T_C | 23280826 | 0.00022 | rs2359637 | T | C | 0.00344455 | -0.0129864 |
| 4 | 4:3407694_G_A | 3407694 | 0.00022 | rs1962767 | G | A | 0.00336432 | -0.0128734 |
| 4 | 4:106336607_C_T | 106336607 | 0.00022 | rs182500825 | C | T | 0.0187519 | -0.0636862 |
| 5 | 5:80493041_C_T | 80493041 | 0.00022 | rs186673840 | C | T | 0.0110702 | 0.0410854 |
| 6 | 6:39776360_C_A | 39776360 | 0.00022 | rs1928191 | C | A | 0.00340229 | -0.0128092 |
| 6 | 6:114229056_A_G | 114229056 | 0.00022 | rs514949 | A | G | 0.00523346 | -0.0187824 |
| 8 | 8:2114663_T_C | 2114663 | 0.00022 | rs9644322 | T | C | 0.00416356 | 0.0152794 |
| 11 | 11:120248493_A_G | 120248493 | 0.00022 | rs58073046 | A | G | 0.00459754 | -0.0178731 |
| 12 | 12:113142365_G_T | 113142365 | 0.00022 | rs78451961 | G | T | 0.00456706 | -0.0168657 |
| 13 | 13:39430314_C_T | 39430314 | 0.00022 | rs9548509 | C | T | 0.0101758 | -0.034781 |
| 13 | 13:62994245_C_T | 62994245 | 0.00022 | rs146325974 | C | T | 0.018384 | 0.0657082 |
| 13 | 13:101161048_G_A | 101161048 | 0.00022 | rs837294 | G | A | 0.0148412 | 0.0528839 |
| 14 | 14:59491469_G_A | 59491469 | 0.00022 | rs970700 | G | A | 0.00354548 | 0.0135215 |
| 16 | 16:6130287_A_G | 6130287 | 0.00022 | rs976200 | A | G | 0.00548423 | 0.0191226 |
| 17 | 17:43173273_T_C | 43173273 | 0.00022 | rs2301597 | T | C | 0.00395081 | 0.0145961 |
| 18 | 18:21207719_A_G | 21207719 | 0.00022 | rs77369789 | A | G | 0.00814295 | 0.0285437 |
| 18 | 18:74805059_A_G | 74805059 | 0.00022 | rs76472188 | A | G | 0.00370363 | -0.0135506 |
| 1 | 1:173747659_G_A | 173747659 | 0.00023 | rs184934726 | G | A | 0.0107232 | -0.0404265 |
| 1 | 1:208956608_G_A | 208956608 | 0.00023 | rs148014580 | G | A | 0.0123921 | 0.0400961 |
| 2 | 2:217924153_A_G | 217924153 | 0.00023 | rs7561918 | A | G | 0.00461843 | -0.0172946 |
| 5 | 5:178766570_G_A | 178766570 | 0.00023 | rs55722639 | G | A | 0.00472049 | 0.0168791 |
| 6 | 6:6882525_T_C | 6882525 | 0.00023 | rs9392130 | T | C | 0.00636527 | -0.0221818 |
| 6 | 6:30365320_C_T | 30365320 | 0.00023 | rs1264569 | C | T | 0.0078424 | 0.0287714 |
| 6 | 6:134996762_C_T | 134996762 | 0.00023 | rs11965686 | C | T | 0.0112206 | -0.0400143 |
| 7 | 7:27548628_G_T | 27548628 | 0.00023 | 7:27548628:G:T | G | T | 0.0159563 | -0.0574882 |
| 7 | 7:84046034_G_T | 84046034 | 0.00023 | rs575475189 | G | T | 0.0178131 | 0.0624006 |
| 7 | 7:122043575_G_A | 122043575 | 0.00023 | rs55997702 | G | A | 0.00640266 | -0.0241323 |
| 8 | 8:74175913_G_A | 74175913 | 0.00023 | rs1895259 | G | A | 0.00343305 | 0.0130631 |
| 8 | 8:75619633_G_T | 75619633 | 0.00023 | rs78610045 | G | T | 0.0115709 | 0.0418648 |
| 9 | 9:25234239_A_C | 25234239 | 0.00023 | 9:25234239:A:C | A | C | 0.0175311 | 0.0659801 |
| 9 | 9:128239920_T_C | 128239920 | 0.00023 | rs7038668 | T | C | 0.00332594 | 0.0124817 |
| 10 | 10:13197332_A_G | 13197332 | 0.00023 | rs200035853 | A | G | 0.00623251 | -0.0229374 |
| 10 | 10:18307329_G_A | 18307329 | 0.00023 | rs9988676 | G | A | 0.00918536 | 0.0337958 |
| 10 | 10:103939622_G_A | 103939622 | 0.00023 | rs147208097 | G | A | 0.0131156 | 0.0496491 |
| 11 | 11:72321872_G_A | 72321872 | 0.00023 | rs4944566 | G | A | 0.0040924 | -0.0154193 |
| 11 | 11:108323862_T_C | 108323862 | 0.00023 | rs145818378 | T | C | 0.00827399 | -0.0297056 |
| 11 | 11:130777431_T_C | 130777431 | 0.00023 | rs189493702 | T | C | 0.0166719 | 0.0567238 |
| 12 | 12:84679520_G_T | 84679520 | 0.00023 | rs11116293 | G | T | 0.00573335 | -0.0215007 |
| 16 | 16:70733138_G_A | 70733138 | 0.00023 | rs4985563 | G | A | 0.00391763 | 0.0146573 |
| 17 | 17:45965532_G_A | 45965532 | 0.00023 | rs144199883 | G | A | 0.016082 | 0.0586783 |
| 19 | 19:46427385_C_T | 46427385 | 0.00023 | rs139648189 | C | T | 0.00577818 | -0.0196807 |
| 21 | 21:44796332_G_A | 44796332 | 0.00023 | rs73906513 | G | A | 0.0067232 | -0.024164 |
| 5 | 5:51987238_G_A | 51987238 | 0.00024 | rs350450 | G | A | 0.00369674 | -0.0131369 |
| 5 | 5:53005969_A_G | 53005969 | 0.00024 | rs449160 | A | G | 0.00360454 | 0.0138034 |
| 6 | 6:28697859_A_G | 28697859 | 0.00024 | rs144803253 | A | G | 0.0132908 | -0.0453774 |
| 7 | 7:8029516_A_G | 8029516 | 0.00024 | rs9640055 | A | G | 0.00431085 | 0.0148958 |
| 7 | 7:13866844_C_T | 13866844 | 0.00024 | rs10265181 | C | T | 0.00389236 | 0.014261 |
| 7 | 7:28039641_T_C | 28039641 | 0.00024 | rs117554813 | T | C | 0.00832164 | 0.0314186 |
| 8 | 8:17489468_T_C | 17489468 | 0.00024 | rs28396775 | T | C | 0.010946 | 0.0389462 |
| 9 | 9:26221794_T_C | 26221794 | 0.00024 | rs73645325 | T | C | 0.00703357 | -0.0262371 |
| 9 | 9:134984343_G_A | 134984343 | 0.00024 | rs11243629 | G | A | 0.00368839 | 0.0133928 |
| 11 | 11:69843505_G_A | 69843505 | 0.00024 | rs12797741 | G | A | 0.00365959 | 0.0134613 |
| 11 | 11:119806852_G_A | 119806852 | 0.00024 | rs548429 | G | A | 0.00374156 | 0.0133577 |
| 12 | 12:53441607_C_T | 53441607 | 0.00024 | rs7304594 | C | T | 0.00484493 | -0.0184886 |
| 13 | 13:58826854_C_T | 58826854 | 0.00024 | rs7319627 | C | T | 0.00379156 | 0.014345 |
| 13 | 13:97357355_A_C | 97357355 | 0.00024 | rs138733494 | A | C | 0.01007 | -0.0395788 |
| 14 | 14:61671033_C_T | 61671033 | 0.00024 | rs12587353 | C | T | 0.00512551 | 0.0184152 |
| 15 | 15:70139250_G_A | 70139250 | 0.00024 | rs304998 | G | A | 0.00340099 | 0.0130188 |
| 15 | 15:91171220_C_T | 91171220 | 0.00024 | rs181718576 | C | T | 0.00729082 | 0.0257953 |
| 16 | 16:69786092_C_A | 69786092 | 0.00024 | rs8045682 | C | A | 0.00356014 | -0.0128218 |
| 17 | 17:37497862_A_G | 37497862 | 0.00024 | rs193111360 | A | G | 0.0165188 | 0.0603877 |
| 18 | 18:19780216_G_A | 19780216 | 0.00024 | rs16964670 | G | A | 0.00485006 | -0.018197 |
| 18 | 18:63826878_T_C | 63826878 | 0.00024 | rs1532175 | T | C | 0.00354795 | 0.0126633 |
| 20 | 20:10688744_C_A | 10688744 | 0.00024 | rs6108676 | C | A | 0.00347443 | 0.013417 |
| 20 | 20:42726111_C_T | 42726111 | 0.00024 | rs6103619 | C | T | 0.00335766 | -0.0126794 |
| 2 | 2:33660211_A_G | 33660211 | 0.00025 | rs597593 | A | G | 0.00760136 | -0.0252386 |
| 2 | 2:199582469_G_A | 199582469 | 0.00025 | rs78178139 | G | A | 0.0135106 | 0.0474186 |
| 10 | 10:104569983_C_T | 104569983 | 0.00025 | rs76631236 | C | T | 0.0119818 | -0.0444166 |
| 12 | 12:23639392_G_A | 23639392 | 0.00025 | rs79259102 | G | A | 0.00550556 | -0.0195099 |
| 12 | 12:24503958_T_C | 24503958 | 0.00025 | rs486558 | T | C | 0.00424096 | 0.0143936 |
| 12 | 12:110082115_T_C | 110082115 | 0.00025 | rs1344543 | T | C | 0.00334503 | 0.0114829 |
| 17 | 17:13234888_C_T | 13234888 | 0.00025 | rs193046775 | C | T | 0.0143542 | -0.0526922 |
| 1 | 1:26758773_G_A | 26758773 | 0.00026 | rs6656196 | G | A | 0.00394406 | 0.0134247 |
| 1 | 1:56060981_C_T | 56060981 | 0.00026 | rs11206628 | C | T | 0.00340883 | 0.0120836 |
| 1 | 1:77966230_G_A | 77966230 | 0.00026 | rs3104464 | G | A | 0.00342602 | -0.0123417 |
| 2 | 2:145228701_A_G | 145228701 | 0.00026 | rs6430059 | A | G | 0.00334868 | 0.0117666 |
| 5 | 5:149099129_G_A | 149099129 | 0.00026 | rs7730770 | G | A | 0.00367481 | -0.013803 |
| 6 | 6:10419819_C_T | 10419819 | 0.00026 | rs143945720 | C | T | 0.0156812 | 0.0594473 |
| 6 | 6:109527841_C_T | 109527841 | 0.00026 | rs352854 | C | T | 0.00369534 | -0.0139521 |
| 12 | 12:16524170_A_G | 16524170 | 0.00026 | rs7313177 | A | G | 0.00401632 | -0.0150707 |
| 12 | 12:120830631_T_C | 120830631 | 0.00026 | rs145828988 | T | C | 0.00629598 | -0.0230265 |
| 17 | 17:46174910_C_T | 46174910 | 0.00026 | rs1033985943 | C | T | 0.0155432 | 0.0599197 |
| 1 | 1:87799284_T_C | 87799284 | 0.00027 | rs3795314 | T | C | 0.00409695 | 0.0157808 |
| 1 | 1:162892647_C_T | 162892647 | 0.00027 | rs10494378 | C | T | 0.0036012 | 0.0125252 |
| 1 | 1:168411432_G_A | 168411432 | 0.00027 | rs16860596 | G | A | 0.00627768 | 0.0214968 |
| 1 | 1:228321742_G_A | 228321742 | 0.00027 | rs142227736 | G | A | 0.014984 | -0.0519207 |
| 2 | 2:71351487_G_A | 71351487 | 0.00027 | rs11541017 | G | A | 0.00659081 | 0.0236367 |
| 2 | 2:85975594_C_T | 85975594 | 0.00027 | rs17026610 | C | T | 0.00393384 | 0.0145414 |
| 2 | 2:185519250_T_C | 185519250 | 0.00027 | rs76692397 | T | C | 0.0115004 | 0.0404217 |
| 2 | 2:211991518_C_A | 211991518 | 0.00027 | rs13400493 | C | A | 0.00349856 | -0.013343 |
| 4 | 4:149067678_C_T | 149067678 | 0.00027 | rs28482137 | C | T | 0.00798699 | 0.0283924 |
| 4 | 4:157370363_T_C | 157370363 | 0.00027 | rs2220588 | T | C | 0.00404361 | -0.0143129 |
| 5 | 5:58575346_C_T | 58575346 | 0.00027 | 5:58575346:C:T | C | T | 0.0125858 | 0.0459926 |
| 5 | 5:107115667_C_T | 107115667 | 0.00027 | rs770183 | C | T | 0.00349752 | 0.0123751 |
| 6 | 6:126572573_G_A | 126572573 | 0.00027 | rs9398797 | G | A | 0.00360238 | -0.0128838 |
| 6 | 6:162946697_T_C | 162946697 | 0.00027 | rs2846508 | T | C | 0.00333437 | 0.0120639 |
| 9 | 9:130971018_A_G | 130971018 | 0.00027 | rs7875406 | A | G | 0.00372561 | -0.01404 |
| 11 | 11:78110826_C_A | 78110826 | 0.00027 | rs180959251 | C | A | 0.0195974 | -0.0699409 |
| 12 | 12:128745548_C_T | 128745548 | 0.00027 | rs58687679 | C | T | 0.00666755 | -0.0219454 |
| 13 | 13:54471824_C_A | 54471824 | 0.00027 | rs9316673 | C | A | 0.00335172 | -0.0121933 |
| 16 | 16:81831781_T_C | 81831781 | 0.00027 | rs4889385 | T | C | 0.00632327 | 0.0221301 |
| 17 | 17:35858039_C_T | 35858039 | 0.00027 | rs34711199 | C | T | 0.00340586 | 0.0122636 |
| 17 | 17:45007213_A_G | 45007213 | 0.00027 | rs2072317 | A | G | 0.00337936 | 0.011814 |
| 20 | 20:57739809_C_T | 57739809 | 0.00027 | rs73306874 | C | T | 0.00613118 | -0.0227265 |
| 21 | 21:44457438_A_C | 44457438 | 0.00027 | rs414224 | A | C | 0.00372206 | -0.0132963 |
| 1 | 1:151550673_G_A | 151550673 | 0.00028 | rs12751350 | G | A | 0.00334226 | -0.0127578 |
| 1 | 1:155102577_T_G | 155102577 | 0.00028 | rs139822562 | T | G | 0.0140711 | -0.0472837 |
| 3 | 3:184102052_T_C | 184102052 | 0.00028 | rs58834031 | T | C | 0.00436357 | -0.0166948 |
| 4 | 4:152284391_C_T | 152284391 | 0.00028 | rs12503602 | C | T | 0.00338405 | 0.0116775 |
| 5 | 5:174419911_G_A | 174419911 | 0.00028 | rs62389785 | G | A | 0.0127617 | -0.0461681 |
| 6 | 6:155607022_T_G | 155607022 | 0.00028 | rs188220445 | T | G | 0.0101302 | -0.0366906 |
| 7 | 7:138335882_G_T | 138335882 | 0.00028 | rs79167264 | G | T | 0.016042 | 0.0541968 |
| 8 | 8:18071095_A_G | 18071095 | 0.00028 | rs4921581 | A | G | 0.00338986 | -0.0125357 |
| 9 | 9:122980750_A_G | 122980750 | 0.00028 | rs77023416 | A | G | 0.00903983 | -0.0343217 |
| 9 | 9:123433943_G_A | 123433943 | 0.00028 | rs189086231 | G | A | 0.0104885 | -0.0414456 |
| 10 | 10:103040585_G_A | 103040585 | 0.00028 | rs150444603 | G | A | 0.0128846 | -0.0470754 |
| 10 | 10:105354443_G_A | 105354443 | 0.00028 | rs2295588 | G | A | 0.00815541 | 0.0289942 |
| 10 | 10:125297295_A_G | 125297295 | 0.00028 | rs10510132 | A | G | 0.0052291 | -0.0184024 |
| 13 | 13:98907268_G_A | 98907268 | 0.00028 | rs4477549 | G | A | 0.00710471 | -0.0246323 |
| 16 | 16:87689186_C_T | 87689186 | 0.00028 | rs12598063 | C | T | 0.00356496 | 0.012379 |
| 19 | 19:5035007_A_C | 5035007 | 0.00028 | rs263056 | A | C | 0.00417625 | 0.0152395 |
| 20 | 20:19008025_G_T | 19008025 | 0.00028 | rs755981 | G | T | 0.00531019 | 0.0188092 |
| 1 | 1:10780727_A_G | 10780727 | 0.00029 | rs706007 | A | G | 0.00405606 | -0.0142652 |
| 1 | 1:83978738_T_C | 83978738 | 0.00029 | rs12134784 | T | C | 0.00538396 | 0.0198276 |
| 1 | 1:115069442_C_T | 115069442 | 0.00029 | rs147276148 | C | T | 0.00917164 | -0.0321075 |
| 1 | 1:227586880_T_C | 227586880 | 0.00029 | rs12040226 | T | C | 0.00766082 | 0.0271154 |
| 1 | 1:248251633_C_T | 248251633 | 0.00029 | rs553892203 | C | T | 0.0175125 | 0.0616336 |
| 3 | 3:41680180_C_A | 41680180 | 0.00029 | rs140338870 | C | A | 0.0146185 | -0.0547479 |
| 4 | 4:91026716_A_C | 91026716 | 0.00029 | rs117370618 | A | C | 0.0104248 | 0.0371707 |
| 6 | 6:114118746_A_G | 114118746 | 0.00029 | rs7751817 | A | G | 0.00497942 | 0.0183652 |
| 6 | 6:170899414_G_A | 170899414 | 0.00029 | rs12525596 | G | A | 0.0112153 | -0.0389179 |
| 8 | 8:81075583_A_G | 81075583 | 0.00029 | rs6473209 | A | G | 0.00386216 | 0.0133291 |
| 8 | 8:144054295_C_T | 144054295 | 0.00029 | rs13269431 | C | T | 0.00482892 | -0.0174153 |
| 9 | 9:92017733_G_A | 92017733 | 0.00029 | rs41287361 | G | A | 0.00382497 | 0.0139179 |
| 9 | 9:126495161_T_C | 126495161 | 0.00029 | rs80176232 | T | C | 0.0136601 | -0.0465914 |
| 10 | 10:105518428_G_A | 105518428 | 0.00029 | rs74773930 | G | A | 0.0146648 | 0.0540782 |
| 12 | 12:52241706_C_T | 52241706 | 0.00029 | rs303782 | C | T | 0.00376072 | 0.0136244 |
| 12 | 12:119587366_G_A | 119587366 | 0.00029 | rs73213433 | G | A | 0.00638455 | 0.0225649 |
| 13 | 13:63420706_C_T | 63420706 | 0.00029 | rs180752318 | C | T | 0.0178148 | 0.0625275 |
| 16 | 16:24690610_G_T | 24690610 | 0.00029 | 16:24690610:G:T | G | T | 0.0201883 | -0.0687417 |
| 16 | 16:27371527_G_T | 27371527 | 0.00029 | rs192952825 | G | T | 0.0160393 | -0.0588086 |
| 16 | 16:86089788_C_T | 86089788 | 0.00029 | rs199950649 | C | T | 0.0152152 | -0.0521774 |
| 17 | 17:10143291_T_G | 10143291 | 0.00029 | rs34343029 | T | G | 0.0104714 | 0.0355783 |
| 17 | 17:56005007_A_G | 56005007 | 0.00029 | rs73314294 | A | G | 0.0116909 | 0.0406052 |
| 19 | 19:7486381_T_C | 7486381 | 0.00029 | rs12978425 | T | C | 0.00340864 | -0.0123511 |
| 2 | 2:140730955_A_G | 140730955 | 3E-04 | rs79441497 | A | G | 0.00582176 | -0.0220227 |
| 3 | 3:119168235_A_C | 119168235 | 3E-04 | rs186678359 | A | C | 0.00917911 | 0.0332126 |
| 6 | 6:31745284_C_T | 31745284 | 3E-04 | rs2736426 | C | T | 0.00344195 | -0.012624 |
| 7 | 7:27882745_G_A | 27882745 | 3E-04 | rs146717029 | G | A | 0.016342 | -0.0575418 |
| 8 | 8:19636401_A_G | 19636401 | 3E-04 | rs141933287 | A | G | 0.0128948 | -0.0468341 |
| 8 | 8:105666107_A_G | 105666107 | 3E-04 | rs16871642 | A | G | 0.00389666 | -0.0142995 |
| 11 | 11:32455527_C_A | 32455527 | 3E-04 | rs3930513 | C | A | 0.00377222 | -0.0125366 |
| 11 | 11:128666107_A_G | 128666107 | 3E-04 | rs149715792 | A | G | 0.0107972 | -0.0370892 |
| 13 | 13:88482290_T_G | 88482290 | 3E-04 | rs190567455 | T | G | 0.0158539 | 0.055732 |
| 14 | 14:69339834_C_T | 69339834 | 3E-04 | rs10129289 | C | T | 0.00472224 | -0.0158218 |
| 17 | 17:1968986_A_G | 1968986 | 3E-04 | rs2232485 | A | G | 0.00349025 | 0.0128027 |
| 17 | 17:63448326_C_A | 63448326 | 3E-04 | rs8069448 | C | A | 0.00350233 | -0.0123888 |
| 20 | 20:943461_C_T | 943461 | 3E-04 | rs515349 | C | T | 0.00533948 | -0.0178202 |
| 1 | 1:188067782_A_G | 188067782 | 0.00031 | rs12031312 | A | G | 0.00385598 | -0.0134809 |
| 2 | 2:68259408_G_A | 68259408 | 0.00031 | rs79076069 | G | A | 0.0123149 | -0.0458608 |
| 4 | 4:37477042_A_G | 37477042 | 0.00031 | rs1463573 | A | G | 0.0039487 | 0.0139011 |
| 4 | 4:88969043_T_C | 88969043 | 0.00031 | rs146105174 | T | C | 0.0155247 | -0.0552324 |
| 4 | 4:106994648_C_T | 106994648 | 0.00031 | rs56222430 | C | T | 0.00428113 | -0.0160424 |
| 5 | 5:57755703_C_T | 57755703 | 0.00031 | rs3211270 | C | T | 0.00342889 | -0.0123907 |
| 6 | 6:90607031_C_T | 90607031 | 0.00031 | rs79510874 | C | T | 0.0135193 | 0.0509501 |
| 10 | 10:45384409_G_A | 45384409 | 0.00031 | rs1570985 | G | A | 0.00459209 | -0.015933 |
| 10 | 10:65367593_G_T | 65367593 | 0.00031 | rs141660877 | G | T | 0.0123687 | 0.0450824 |
| 11 | 11:8852239_C_T | 8852239 | 0.00031 | rs2243566 | C | T | 0.00378589 | -0.0136713 |
| 12 | 12:14168489_C_T | 14168489 | 0.00031 | rs147696847 | C | T | 0.014238 | 0.0523568 |
| 12 | 12:41648699_G_A | 41648699 | 0.00031 | rs1431121 | G | A | 0.00348897 | 0.011759 |
| 16 | 16:994699_T_C | 994699 | 0.00031 | rs12926731 | T | C | 0.00371935 | -0.013774 |
| 19 | 19:46343257_C_T | 46343257 | 0.00031 | rs76846311 | C | T | 0.00351568 | 0.012584 |
| 22 | 22:30669883_G_A | 30669883 | 0.00031 | rs6006426 | G | A | 0.00339823 | 0.0121732 |
| 1 | 1:9912137_G_A | 9912137 | 0.00032 | rs138578737 | G | A | 0.0107308 | 0.0379377 |
| 2 | 2:176675940_A_G | 176675940 | 0.00032 | rs10930710 | A | G | 0.00349375 | -0.0119565 |
| 3 | 3:29560152_C_A | 29560152 | 0.00032 | rs184074767 | C | A | 0.017007 | -0.0557511 |
| 4 | 4:105719324_T_C | 105719324 | 0.00032 | rs79947365 | T | C | 0.0185269 | -0.0638666 |
| 4 | 4:120058168_A_G | 120058168 | 0.00032 | rs57970878 | A | G | 0.00362181 | -0.0137156 |
| 7 | 7:6515183_T_C | 6515183 | 0.00032 | rs17136294 | T | C | 0.00739279 | -0.0277314 |
| 7 | 7:139444024_C_T | 139444024 | 0.00032 | rs10215942 | C | T | 0.00361173 | 0.0138936 |
| 7 | 7:155169497_A_G | 155169497 | 0.00032 | rs1421317 | A | G | 0.00900886 | -0.0332463 |
| 10 | 10:45963830_G_A | 45963830 | 0.00032 | rs4948675 | G | A | 0.00598473 | 0.0217043 |
| 11 | 11:5481141_C_T | 5481141 | 0.00032 | rs145812031 | C | T | 0.0158658 | -0.0532518 |
| 11 | 11:72520147_G_T | 72520147 | 0.00032 | 11:72520147:G:T | G | T | 0.00531738 | 0.0179039 |
| 11 | 11:82498536_C_T | 82498536 | 0.00032 | rs471755 | C | T | 0.00406539 | 0.0137448 |
| 12 | 12:1016910_T_C | 1016910 | 0.00032 | rs2277869 | T | C | 0.00466737 | -0.0178481 |
| 13 | 13:96600246_T_C | 96600246 | 0.00032 | rs117944539 | T | C | 0.0108699 | -0.0363404 |
| 13 | 13:113889127_A_G | 113889127 | 0.00032 | rs2287246 | A | G | 0.00368315 | -0.0137898 |
| 2 | 2:29820586_T_C | 29820586 | 0.00033 | rs6733973 | T | C | 0.00420504 | 0.0149134 |
| 2 | 2:55089897_C_A | 55089897 | 0.00033 | rs10186764 | C | A | 0.00353251 | 0.0119828 |
| 2 | 2:152309963_C_T | 152309963 | 0.00033 | rs141611911 | C | T | 0.0156176 | 0.0564671 |
| 4 | 4:8594683_C_T | 8594683 | 0.00033 | rs2302582 | C | T | 0.00406264 | 0.0141353 |
| 4 | 4:30837994_G_A | 30837994 | 0.00033 | rs918381504 | G | A | 0.0202031 | 0.0703614 |
| 5 | 5:24988228_T_C | 24988228 | 0.00033 | rs192245788 | T | C | 0.0136765 | 0.0453253 |
| 6 | 6:43710381_C_T | 43710381 | 0.00033 | rs10807290 | C | T | 0.00344457 | -0.0121067 |
| 6 | 6:92176663_A_C | 92176663 | 0.00033 | rs10806445 | A | C | 0.0034401 | 0.0126523 |
| 6 | 6:108467024_G_A | 108467024 | 0.00033 | rs218291 | G | A | 0.0042754 | -0.0153037 |
| 7 | 7:64781660_C_A | 64781660 | 0.00033 | rs6973096 | C | A | 0.00358728 | -0.012719 |
| 9 | 9:25294481_T_C | 25294481 | 0.00033 | rs12554573 | T | C | 0.00474083 | 0.0177791 |
| 11 | 11:45793023_T_G | 45793023 | 0.00033 | rs2666890 | T | G | 0.00336756 | 0.0116527 |
| 12 | 12:13429460_A_C | 13429460 | 0.00033 | rs11832072 | A | C | 0.00594574 | -0.0208169 |
| 12 | 12:27793571_C_A | 27793571 | 0.00033 | rs1964317 | C | A | 0.00353551 | 0.0122341 |
| 12 | 12:125519424_G_A | 125519424 | 0.00033 | rs148504100 | G | A | 0.0103649 | -0.0365576 |
| 19 | 19:35669071_G_A | 35669071 | 0.00033 | rs4805110 | G | A | 0.00371936 | -0.0140697 |
| 20 | 20:43575062_G_A | 43575062 | 0.00033 | rs145568277 | G | A | 0.00963433 | -0.0342347 |
| 22 | 22:44795976_G_A | 44795976 | 0.00033 | rs72619564 | G | A | 0.00407733 | -0.0140436 |
| 1 | 1:28600607_A_G | 28600607 | 0.00034 | rs2274848 | A | G | 0.00574501 | -0.0211426 |
| 1 | 1:53129119_T_C | 53129119 | 0.00034 | rs269322 | T | C | 0.0138925 | 0.0470697 |
| 1 | 1:219604008_C_T | 219604008 | 0.00034 | rs17528105 | C | T | 0.00735541 | -0.0262512 |
| 3 | 3:57115552_G_T | 57115552 | 0.00034 | rs77366256 | G | T | 0.0175277 | 0.0625916 |
| 3 | 3:150136035_G_A | 150136035 | 0.00034 | rs200638682 | G | A | 0.00847387 | -0.0283447 |
| 4 | 4:93019268_A_G | 93019268 | 0.00034 | rs75692041 | A | G | 0.00652673 | -0.023374 |
| 4 | 4:147658082_T_C | 147658082 | 0.00034 | rs10027612 | T | C | 0.00333637 | -0.0118739 |
| 6 | 6:52431403_C_T | 52431403 | 0.00034 | rs2268708 | C | T | 0.0033829 | 0.0116524 |
| 6 | 6:73914453_A_G | 73914453 | 0.00034 | rs57535819 | A | G | 0.00406027 | 0.0153268 |
| 6 | 6:108451893_A_G | 108451893 | 0.00034 | rs218294 | A | G | 0.00336567 | 0.0122522 |
| 7 | 7:49925058_G_A | 49925058 | 0.00034 | rs78589696 | G | A | 0.0075107 | 0.0251816 |
| 10 | 10:3524778_G_A | 3524778 | 0.00034 | rs74825782 | G | A | 0.0111774 | -0.0379985 |
| 10 | 10:28932128_G_A | 28932128 | 0.00034 | rs4749330 | G | A | 0.00461649 | -0.0162226 |
| 14 | 14:69068384_A_G | 69068384 | 0.00034 | rs12878344 | A | G | 0.00379334 | 0.013928 |
| 16 | 16:28582142_G_T | 28582142 | 0.00034 | rs193628 | G | T | 0.0038417 | 0.0144649 |
| 17 | 17:55307502_G_A | 55307502 | 0.00034 | 17:55307502:G:A | G | A | 0.00446141 | -0.016564 |
| 18 | 18:4001419_G_A | 4001419 | 0.00034 | rs10853309 | G | A | 0.00356188 | 0.0128157 |
| 1 | 1:228364605_T_C | 228364605 | 0.00035 | rs4653915 | T | C | 0.00355509 | 0.0123552 |
| 2 | 2:24994570_T_C | 24994570 | 0.00035 | rs9309308 | T | C | 0.00547954 | -0.018692 |
| 3 | 3:104666976_T_C | 104666976 | 0.00035 | rs13066237 | T | C | 0.0053846 | -0.0189569 |
| 3 | 3:105889064_A_G | 105889064 | 0.00035 | rs9872358 | A | G | 0.00360857 | -0.0129843 |
| 3 | 3:115244309_C_T | 115244309 | 0.00035 | rs73233838 | C | T | 0.00408823 | 0.014616 |
| 4 | 4:10597569_T_C | 10597569 | 0.00035 | rs1004325 | T | C | 0.0038235 | -0.0138452 |
| 6 | 6:118751839_C_A | 118751839 | 0.00035 | rs148380056 | C | A | 0.00623492 | 0.0228697 |
| 6 | 6:122303030_A_G | 122303030 | 0.00035 | rs2684275 | A | G | 0.0052393 | 0.0187482 |
| 9 | 9:24755397_G_T | 24755397 | 0.00035 | rs12005335 | G | T | 0.00433608 | -0.0163509 |
| 11 | 11:12183036_A_C | 12183036 | 0.00035 | rs7131034 | A | C | 0.00345612 | 0.0122426 |
| 11 | 11:70535575_C_T | 70535575 | 0.00035 | rs75955930 | C | T | 0.00751995 | 0.0243552 |
| 14 | 14:60733590_G_A | 60733590 | 0.00035 | rs116938873 | G | A | 0.00687893 | -0.0255606 |
| 15 | 15:41368514_C_T | 41368514 | 0.00035 | rs149029244 | C | T | 0.0146997 | -0.0519618 |
| 17 | 17:61562774_T_C | 61562774 | 0.00035 | 17:61562774:T:C | T | C | 0.00347213 | 0.0120657 |
| 20 | 20:39993341_C_T | 39993341 | 0.00035 | rs77201844 | C | T | 0.00373769 | -0.0124082 |
| 2 | 2:68143595_T_C | 68143595 | 0.00036 | rs872108 | T | C | 0.00336766 | 0.0119581 |
| 4 | 4:25090004_G_A | 25090004 | 0.00036 | rs4697545 | G | A | 0.00469422 | 0.0166436 |
| 5 | 5:146091590_G_T | 146091590 | 0.00036 | rs3851488 | G | T | 0.00343082 | 0.0123941 |
| 5 | 5:152121302_C_A | 152121302 | 0.00036 | 5:152121302:C:A | C | A | 0.0135241 | -0.0465437 |
| 9 | 9:101964013_A_G | 101964013 | 0.00036 | rs10988749 | A | G | 0.00533341 | -0.0186529 |
| 10 | 10:48441659_G_T | 48441659 | 0.00036 | rs4922500 | G | T | 0.0050309 | -0.0181482 |
| 11 | 11:10274896_T_C | 10274896 | 0.00036 | rs575892492 | T | C | 0.0144444 | 0.0538968 |
| 1 | 1:7911228_C_T | 7911228 | 0.00037 | 1:7911228:C:T | C | T | 0.00616751 | -0.0208116 |
| 2 | 2:162967870_T_C | 162967870 | 0.00037 | rs148992659 | T | C | 0.0164892 | -0.0631533 |
| 2 | 2:164752160_T_C | 164752160 | 0.00037 | rs7595546 | T | C | 0.00402772 | 0.0140578 |
| 2 | 2:170177382_G_A | 170177382 | 0.00037 | 2:170177382:G:A | G | A | 0.00942854 | -0.0366977 |
| 4 | 4:177479140_G_A | 177479140 | 0.00037 | rs7665613 | G | A | 0.00359598 | -0.0134511 |
| 6 | 6:26327900_C_T | 26327900 | 0.00037 | 6:26327900:C:T | C | T | 0.00952436 | -0.0346039 |
| 7 | 7:41318825_T_C | 41318825 | 0.00037 | rs17716754 | T | C | 0.00418731 | 0.0146333 |
| 12 | 12:126796885_A_G | 126796885 | 0.00037 | rs16920480 | A | G | 0.00354795 | -0.0123923 |
| 15 | 15:83330451_G_A | 83330451 | 0.00037 | rs117933613 | G | A | 0.0101442 | 0.0356711 |
| 16 | 16:49878302_G_A | 49878302 | 0.00037 | rs59369441 | G | A | 0.00872825 | 0.0306642 |
| 17 | 17:37455773_G_A | 37455773 | 0.00037 | rs150560802 | G | A | 0.0143354 | 0.0506539 |
| 18 | 18:65231955_T_C | 65231955 | 0.00037 | rs56048830 | T | C | 0.00776056 | -0.0276314 |
| 18 | 18:68698561_C_T | 68698561 | 0.00037 | rs57465160 | C | T | 0.00612145 | -0.0221598 |
| 1 | 1:18105126_A_G | 18105126 | 0.00038 | rs150183557 | A | G | 0.0130774 | 0.0464572 |
| 3 | 3:23766637_G_A | 23766637 | 0.00038 | rs9848071 | G | A | 0.00335392 | 0.0112233 |
| 3 | 3:170762294_G_A | 170762294 | 0.00038 | rs9868403 | G | A | 0.00553588 | -0.0196634 |
| 4 | 4:7429462_A_G | 7429462 | 0.00038 | rs73796570 | A | G | 0.00970136 | 0.0331371 |
| 4 | 4:65304222_A_G | 65304222 | 0.00038 | rs6828446 | A | G | 0.00474303 | -0.015276 |
| 4 | 4:109999694_G_A | 109999694 | 0.00038 | rs149911709 | G | A | 0.0186526 | 0.0659757 |
| 6 | 6:53623730_G_A | 53623730 | 0.00038 | rs4715420 | G | A | 0.00368789 | -0.0126694 |
| 7 | 7:30937178_T_C | 30937178 | 0.00038 | rs1000597 | T | C | 0.00408923 | -0.0144257 |
| 8 | 8:69860376_T_C | 69860376 | 0.00038 | rs73683778 | T | C | 0.00784901 | -0.0283662 |
| 8 | 8:123093181_A_G | 123093181 | 0.00038 | rs11990718 | A | G | 0.00674789 | 0.0231295 |
| 10 | 10:87355633_G_A | 87355633 | 0.00038 | rs143976158 | G | A | 0.0151452 | 0.0517101 |
| 15 | 15:91038778_T_C | 91038778 | 0.00038 | rs147725471 | T | C | 0.00751283 | -0.0283346 |
| 16 | 16:83603142_T_C | 83603142 | 0.00038 | rs7193706 | T | C | 0.00364679 | 0.0132086 |
| 18 | 18:71573242_G_A | 71573242 | 0.00038 | rs934301 | G | A | 0.00442135 | -0.0152643 |
| 19 | 19:11234294_C_T | 11234294 | 0.00038 | rs3826808 | C | T | 0.00770901 | -0.0273032 |
| 19 | 19:17239073_C_T | 17239073 | 0.00038 | rs78015763 | C | T | 0.00547027 | 0.0186726 |
| 20 | 20:40773610_C_T | 40773610 | 0.00038 | rs150626623 | C | T | 0.0154654 | 0.0510678 |
| 20 | 20:43967310_G_A | 43967310 | 0.00038 | rs2284277 | G | A | 0.00349616 | 0.0123497 |
| 22 | 22:29102645_G_A | 29102645 | 0.00038 | rs2003748 | G | A | 0.00692966 | 0.024214 |
| 2 | 2:182046295_A_G | 182046295 | 0.00039 | rs9678124 | A | G | 0.00367145 | -0.0137418 |
| 2 | 2:240292204_C_T | 240292204 | 0.00039 | rs72994578 | C | T | 0.00661934 | 0.0245329 |
| 3 | 3:50509864_G_A | 50509864 | 0.00039 | rs147400454 | G | A | 0.0099711 | 0.0335835 |
| 5 | 5:101558769_C_A | 101558769 | 0.00039 | rs6891076 | C | A | 0.00511206 | -0.0176414 |
| 6 | 6:31323296_A_G | 31323296 | 0.00039 | rs1050747 | A | G | 0.00487455 | 0.0171494 |
| 6 | 6:151981210_A_G | 151981210 | 0.00039 | rs851966 | A | G | 0.00384102 | -0.0133176 |
| 8 | 8:76389520_T_G | 76389520 | 0.00039 | rs552046983 | T | G | 0.0174778 | 0.0618625 |
| 13 | 13:31954683_G_A | 31954683 | 0.00039 | rs77587334 | G | A | 0.00435597 | 0.0152545 |
| 14 | 14:70463247_C_A | 70463247 | 0.00039 | rs17107578 | C | A | 0.00926042 | 0.0325514 |
| 14 | 14:98523202_C_T | 98523202 | 0.00039 | rs864134 | C | T | 0.0041913 | 0.0149667 |
| 17 | 17:9064553_G_A | 9064553 | 0.00039 | rs117830995 | G | A | 0.00733317 | 0.0248794 |
| 1 | 1:5900553_T_C | 5900553 | 4E-04 | rs9727677 | T | C | 0.00817906 | -0.0302767 |
| 2 | 2:104362298_A_G | 104362298 | 4E-04 | rs545791841 | A | G | 0.0122293 | -0.0448921 |
| 2 | 2:165018522_G_T | 165018522 | 4E-04 | rs118001189 | G | T | 0.0107114 | 0.0387486 |
| 3 | 3:49755742_G_T | 49755742 | 4E-04 | rs200455167 | G | T | 0.013433 | -0.0478834 |
| 4 | 4:10810518_T_C | 10810518 | 4E-04 | rs10025258 | T | C | 0.00497239 | 0.0183355 |
| 4 | 4:54813751_G_A | 54813751 | 4E-04 | rs75552861 | G | A | 0.00961088 | -0.0333034 |
| 5 | 5:101878240_C_T | 101878240 | 4E-04 | rs7735587 | C | T | 0.00334522 | -0.011868 |
| 6 | 6:97830324_G_T | 97830324 | 4E-04 | rs202098233 | G | T | 0.013572 | 0.0477771 |
| 9 | 9:131416460_C_T | 131416460 | 4E-04 | rs12344668 | C | T | 0.00371205 | -0.0131284 |
| 14 | 14:23249292_A_G | 23249292 | 4E-04 | 14:23249292:A:G | A | G | 0.00335907 | -0.0120044 |
| 17 | 17:10000083_G_A | 10000083 | 4E-04 | rs72635522 | G | A | 0.00426267 | -0.0146562 |
| 18 | 18:4964403_G_T | 4964403 | 4E-04 | rs60645797 | G | T | 0.00400655 | 0.0135664 |
| 20 | 20:21700016_C_T | 21700016 | 4E-04 | rs73607482 | C | T | 0.00890533 | 0.0311334 |
| 21 | 21:40002077_T_C | 40002077 | 4E-04 | rs59304931 | T | C | 0.00370506 | -0.0136747 |
| 22 | 22:28669990_A_G | 28669990 | 4E-04 | rs189376349 | A | G | 0.0120545 | -0.0418576 |
| 2 | 2:160432819_C_T | 160432819 | 0.00041 | rs17605928 | C | T | 0.00339048 | -0.0122586 |
| 4 | 4:29498211_T_C | 29498211 | 0.00041 | rs61793477 | T | C | 0.00408718 | -0.0144217 |
| 5 | 5:150945824_A_G | 150945824 | 0.00041 | rs145215828 | A | G | 0.0117845 | 0.0419599 |
| 7 | 7:27465460_A_G | 27465460 | 0.00041 | rs139734602 | A | G | 0.0146422 | 0.052191 |
| 8 | 8:64134436_C_T | 64134436 | 0.00041 | rs1048073498 | C | T | 0.0189044 | -0.0698076 |
| 8 | 8:76172697_A_G | 76172697 | 0.00041 | rs2199712 | A | G | 0.00352121 | -0.0117557 |
| 9 | 9:122190384_C_T | 122190384 | 0.00041 | rs10739538 | C | T | 0.00342119 | -0.0114675 |
| 10 | 10:59779063_T_C | 59779063 | 0.00041 | rs144553535 | T | C | 0.016106 | -0.0576145 |
| 11 | 11:69065914_A_G | 69065914 | 0.00041 | rs7129638 | A | G | 0.00335363 | 0.0122416 |
| 13 | 13:26137989_T_C | 26137989 | 0.00041 | rs12871941 | T | C | 0.00381494 | -0.0121448 |
| 16 | 16:59931077_T_C | 59931077 | 0.00041 | rs28681311 | T | C | 0.00361813 | -0.0124226 |
| 17 | 17:35486486_C_T | 35486486 | 0.00041 | rs9789070 | C | T | 0.00343745 | 0.0120563 |
| 18 | 18:59038893_A_G | 59038893 | 0.00041 | rs17068238 | A | G | 0.00511452 | 0.0184208 |
| 19 | 19:29594657_G_A | 29594657 | 0.00041 | rs76455001 | G | A | 0.0129698 | 0.0474269 |
| 20 | 20:54241540_G_A | 54241540 | 0.00041 | rs6092155 | G | A | 0.00674162 | -0.0239735 |
| 20 | 20:55292795_A_G | 55292795 | 0.00041 | rs2426646 | A | G | 0.00427631 | 0.0152416 |
| 4 | 4:81251198_C_T | 81251198 | 0.00042 | rs6811219 | C | T | 0.00384862 | 0.014019 |
| 5 | 5:38218896_C_T | 38218896 | 0.00042 | rs974596 | C | T | 0.00360604 | -0.0124536 |
| 6 | 6:115057337_C_T | 115057337 | 0.00042 | rs9398383 | C | T | 0.00702674 | -0.0238083 |
| 6 | 6:143747849_C_T | 143747849 | 0.00042 | rs79019517 | C | T | 0.0136396 | 0.0468289 |
| 10 | 10:18754495_T_C | 18754495 | 0.00042 | rs11014358 | T | C | 0.00370662 | -0.0130947 |
| 10 | 10:32460050_T_G | 32460050 | 0.00042 | rs2998035 | T | G | 0.00366724 | 0.0130672 |
| 11 | 11:72370260_A_G | 72370260 | 0.00042 | rs878277 | A | G | 0.00341412 | -0.0122192 |
| 12 | 12:19602487_T_C | 19602487 | 0.00042 | rs10841231 | T | C | 0.00375992 | -0.0123926 |
| 12 | 12:53457893_A_G | 53457893 | 0.00042 | rs14369 | A | G | 0.0046247 | 0.0173825 |
| 12 | 12:89941374_G_A | 89941374 | 0.00042 | rs4842665 | G | A | 0.00411973 | 0.0136479 |
| 12 | 12:128256082_A_C | 128256082 | 0.00042 | rs568842151 | A | C | 0.0132921 | 0.0483236 |
| 13 | 13:90190425_T_C | 90190425 | 0.00042 | rs113490380 | T | C | 0.00573454 | 0.0202181 |
| 14 | 14:78621278_C_T | 78621278 | 0.00042 | rs929703 | C | T | 0.00603881 | -0.0210711 |
| 16 | 16:82834549_A_G | 82834549 | 0.00042 | rs62036793 | A | G | 0.00334023 | -0.0116152 |
| 17 | 17:28425232_C_T | 28425232 | 0.00042 | rs201615441 | C | T | 0.0112217 | 0.0370143 |
| 22 | 22:39399738_T_C | 39399738 | 0.00042 | rs4315626 | T | C | 0.00337997 | 0.0122509 |
| 1 | 1:202140228_G_A | 202140228 | 0.00043 | rs7528295 | G | A | 0.00496655 | 0.0175885 |
| 2 | 2:58519929_A_G | 58519929 | 0.00043 | rs76927350 | A | G | 0.00897593 | 0.0300099 |
| 2 | 2:58570335_G_A | 58570335 | 0.00043 | rs67171338 | G | A | 0.00398434 | -0.0135699 |
| 2 | 2:99090524_T_C | 99090524 | 0.00043 | rs3820947 | T | C | 0.00332011 | -0.012388 |
| 5 | 5:174849681_G_A | 174849681 | 0.00043 | rs80321418 | G | A | 0.00869855 | 0.0315224 |
| 5 | 5:180035069_G_A | 180035069 | 0.00043 | rs146943555 | G | A | 0.0045665 | 0.0149768 |
| 6 | 6:121970655_A_G | 121970655 | 0.00043 | rs375621518 | A | G | 0.0170944 | 0.0591383 |
| 7 | 7:79918112_C_A | 79918112 | 0.00043 | rs76237764 | C | A | 0.00782328 | -0.0257613 |
| 8 | 8:26901853_C_T | 26901853 | 0.00043 | rs11786140 | C | T | 0.00582433 | 0.0202144 |
| 9 | 9:75521961_T_C | 75521961 | 0.00043 | rs8187980 | T | C | 0.0108361 | -0.0357735 |
| 11 | 11:89228425_A_G | 89228425 | 0.00043 | rs10765211 | A | G | 0.00350732 | -0.0122486 |
| 14 | 14:35879158_T_C | 35879158 | 0.00043 | rs7160414 | T | C | 0.00490795 | 0.0177666 |
| 17 | 17:60770645_A_G | 60770645 | 0.00043 | rs10328 | A | G | 0.00720737 | 0.0232722 |
| 2 | 2:71678927_T_C | 71678927 | 0.00044 | rs116984886 | T | C | 0.00949087 | 0.0339262 |
| 2 | 2:223588065_T_C | 223588065 | 0.00044 | rs4673033 | T | C | 0.0045458 | 0.0160862 |
| 3 | 3:47027672_G_A | 47027672 | 0.00044 | rs141035799 | G | A | 0.00998621 | -0.036633 |
| 3 | 3:66696197_T_C | 66696197 | 0.00044 | rs79763616 | T | C | 0.00890378 | -0.0307227 |
| 4 | 4:187055089_T_C | 187055089 | 0.00044 | rs4862643 | T | C | 0.00339056 | 0.0117384 |
| 5 | 5:29234529_C_T | 29234529 | 0.00044 | rs4257786 | C | T | 0.00335766 | -0.0123393 |
| 6 | 6:71474869_T_C | 71474869 | 0.00044 | rs144782377 | T | C | 0.0110246 | -0.0372773 |
| 6 | 6:77155660_C_T | 77155660 | 0.00044 | rs12664250 | C | T | 0.00481478 | 0.0178795 |
| 7 | 7:114017246_G_A | 114017246 | 0.00044 | rs12705961 | G | A | 0.00368251 | 0.0129466 |
| 8 | 8:133917588_G_A | 133917588 | 0.00044 | rs2068128 | G | A | 0.00360892 | 0.012921 |
| 9 | 9:9767971_G_A | 9767971 | 0.00044 | rs1174587 | G | A | 0.00548623 | -0.0202823 |
| 9 | 9:28008569_A_G | 28008569 | 0.00044 | rs117562410 | A | G | 0.00571615 | -0.0215787 |
| 9 | 9:33855015_T_C | 33855015 | 0.00044 | rs556913302 | T | C | 0.00390356 | -0.0143298 |
| 10 | 10:56711013_C_T | 56711013 | 0.00044 | rs10763154 | C | T | 0.00336307 | 0.0120845 |
| 13 | 13:80639448_A_G | 80639448 | 0.00044 | rs73238818 | A | G | 0.00944506 | 0.0335239 |
| 15 | 15:66563629_A_G | 66563629 | 0.00044 | rs76598168 | A | G | 0.00707961 | 0.0231149 |
| 19 | 19:45966351_A_C | 45966351 | 0.00044 | rs10413123 | A | C | 0.00345386 | 0.01252 |
| 20 | 20:24305748_C_T | 24305748 | 0.00044 | rs76716649 | C | T | 0.0091293 | 0.0305604 |
| 20 | 20:42317452_C_T | 42317452 | 0.00044 | rs58815529 | C | T | 0.00431759 | 0.0146382 |
| 2 | 2:36694330_T_G | 36694330 | 0.00045 | rs848560 | T | G | 0.00440251 | -0.014379 |
| 2 | 2:40342958_A_C | 40342958 | 0.00045 | rs41280643 | A | C | 0.00526908 | 0.018016 |
| 4 | 4:114791165_T_C | 114791165 | 0.00045 | rs117103806 | T | C | 0.016042 | 0.054284 |
| 5 | 5:38536470_C_T | 38536470 | 0.00045 | rs76622246 | C | T | 0.0039769 | -0.0135354 |
| 5 | 5:173879798_A_G | 173879798 | 0.00045 | rs6556137 | A | G | 0.00334041 | 0.0117348 |
| 6 | 6:109698306_C_T | 109698306 | 0.00045 | rs9480942 | C | T | 0.00344558 | -0.0119005 |
| 8 | 8:13596273_G_A | 13596273 | 0.00045 | rs10100432 | G | A | 0.00341997 | -0.0121428 |
| 9 | 9:121565460_C_T | 121565460 | 0.00045 | rs58109677 | C | T | 0.0159086 | 0.0529282 |
| 10 | 10:71145259_C_T | 71145259 | 0.00045 | 10:71145259:C:T | C | T | 0.00341839 | 0.011781 |
| 11 | 11:25624506_G_A | 25624506 | 0.00045 | rs75023102 | G | A | 0.0141388 | -0.0428247 |
| 11 | 11:90520075_A_G | 90520075 | 0.00045 | rs151218935 | A | G | 0.0127636 | -0.0449935 |
| 12 | 12:96002877_G_A | 96002877 | 0.00045 | rs4762228 | G | A | 0.00334005 | -0.0115313 |
| 16 | 16:83053273_G_A | 83053273 | 0.00045 | rs72794176 | G | A | 0.00395898 | 0.0146531 |
| 17 | 17:7845647_G_A | 7845647 | 0.00045 | rs12950912 | G | A | 0.00415806 | -0.0139609 |
| 19 | 19:11496981_T_C | 11496981 | 0.00045 | rs318719 | T | C | 0.00429623 | -0.0157387 |
| 2 | 2:54623186_C_T | 54623186 | 0.00046 | rs142039456 | C | T | 0.00945152 | -0.0308535 |
| 2 | 2:138476522_T_G | 138476522 | 0.00046 | rs75540221 | T | G | 0.0115297 | -0.039592 |
| 5 | 5:106796200_A_C | 106796200 | 0.00046 | rs152579 | A | C | 0.00370646 | 0.0123119 |
| 6 | 6:27198291_C_A | 27198291 | 0.00046 | rs144465147 | C | A | 0.0126889 | -0.0431843 |
| 6 | 6:31753526_G_A | 31753526 | 0.00046 | rs11751198 | G | A | 0.00651631 | -0.024414 |
| 6 | 6:128312843_G_A | 128312843 | 0.00046 | rs3903662 | G | A | 0.00332835 | -0.0115132 |
| 7 | 7:69334991_C_T | 69334991 | 0.00046 | rs11974201 | C | T | 0.00370134 | -0.0129326 |
| 7 | 7:76813111_C_T | 76813111 | 0.00046 | rs3095465 | C | T | 0.00339577 | -0.0113606 |
| 8 | 8:23136675_T_G | 23136675 | 0.00046 | 8:23136675:T:G | T | G | 0.003471 | 0.0131544 |
| 9 | 9:93187808_C_T | 93187808 | 0.00046 | rs10821428 | C | T | 0.0033572 | -0.0115141 |
| 10 | 10:34012758_A_G | 34012758 | 0.00046 | rs11009502 | A | G | 0.00685844 | 0.0238174 |
| 11 | 11:44119128_C_T | 44119128 | 0.00046 | rs145095567 | C | T | 0.00844355 | -0.029844 |
| 11 | 11:130937747_T_C | 130937747 | 0.00046 | rs11222450 | T | C | 0.00450262 | -0.0152515 |
| 13 | 13:90148759_C_T | 90148759 | 0.00046 | rs78662828 | C | T | 0.0139658 | 0.050258 |
| 15 | 15:69664906_G_A | 69664906 | 0.00046 | rs78170051 | G | A | 0.00582413 | -0.0185233 |
| 15 | 15:100725078_C_T | 100725078 | 0.00046 | rs77894823 | C | T | 0.00697824 | -0.0234006 |
| 17 | 17:48921219_G_A | 48921219 | 0.00046 | 17:48921219:G:A | G | A | 0.00343942 | -0.0119503 |
| 19 | 19:22689369_T_C | 22689369 | 0.00046 | rs117940968 | T | C | 0.00850844 | 0.0275133 |
| 22 | 22:29353612_A_G | 29353612 | 0.00046 | rs147244646 | A | G | 0.0078137 | 0.0268964 |
| 3 | 3:180607539_G_A | 180607539 | 0.00047 | rs140410994 | G | A | 0.0159953 | -0.0566075 |
| 4 | 4:54796220_T_C | 54796220 | 0.00047 | rs11725926 | T | C | 0.0043597 | 0.0140748 |
| 4 | 4:169453433_C_T | 169453433 | 0.00047 | rs62333841 | C | T | 0.00565055 | 0.0172823 |
| 6 | 6:109731504_A_G | 109731504 | 0.00047 | rs77634662 | A | G | 0.0147128 | -0.0530233 |
| 7 | 7:9953271_C_T | 9953271 | 0.00047 | rs548191820 | C | T | 0.0183424 | 0.0592446 |
| 7 | 7:25590299_T_G | 25590299 | 0.00047 | rs2999572 | T | G | 0.00373883 | -0.0135655 |
| 7 | 7:140690655_G_A | 140690655 | 0.00047 | rs9886312 | G | A | 0.00472285 | 0.0176391 |
| 8 | 8:56790861_T_G | 56790861 | 0.00047 | rs182832 | T | G | 0.00425032 | -0.0139984 |
| 10 | 10:33895551_A_C | 33895551 | 0.00047 | rs192032877 | A | C | 0.0157595 | -0.0562721 |
| 14 | 14:26302012_A_G | 26302012 | 0.00047 | rs6574687 | A | G | 0.00413279 | -0.0142992 |
| 17 | 17:16022883_T_C | 16022883 | 0.00047 | rs76010143 | T | C | 0.014923 | 0.0502005 |
| 19 | 19:2306865_C_T | 2306865 | 0.00047 | rs528923634 | C | T | 0.0218954 | 0.0713984 |
| 21 | 21:46648041_C_T | 46648041 | 0.00047 | rs34806772 | C | T | 0.00511986 | 0.0181941 |
| 1 | 1:58147592_C_T | 58147592 | 0.00048 | rs141843909 | C | T | 0.0162508 | -0.0551321 |
| 1 | 1:201430683_G_A | 201430683 | 0.00048 | rs4915523 | G | A | 0.00374265 | -0.012311 |
| 1 | 1:203140671_G_A | 203140671 | 0.00048 | rs3737875 | G | A | 0.00343188 | -0.0120979 |
| 1 | 1:234633134_C_T | 234633134 | 0.00048 | rs4027051 | C | T | 0.00803467 | 0.0283561 |
| 2 | 2:58168831_A_G | 58168831 | 0.00048 | rs2717038 | A | G | 0.00333104 | 0.0108953 |
| 2 | 2:79391622_C_A | 79391622 | 0.00048 | 2:79391622:C:A | C | A | 0.00455224 | 0.0169399 |
| 2 | 2:99009588_G_A | 99009588 | 0.00048 | 2:99009588:G:A | G | A | 0.0146442 | -0.0528072 |
| 3 | 3:14478434_G_A | 14478434 | 0.00048 | rs13086679 | G | A | 0.00374396 | 0.0136932 |
| 3 | 3:129650840_C_T | 129650840 | 0.00048 | rs1398709 | C | T | 0.00376281 | -0.0125749 |
| 3 | 3:195281162_C_T | 195281162 | 0.00048 | rs66516606 | C | T | 0.00760023 | 0.0257642 |
| 5 | 5:138657944_C_A | 138657944 | 0.00048 | rs3816021 | C | A | 0.00373937 | -0.01275 |
| 6 | 6:55906935_C_A | 55906935 | 0.00048 | rs2745749 | C | A | 0.00654433 | 0.0220294 |
| 6 | 6:119460786_G_A | 119460786 | 0.00048 | rs9372523 | G | A | 0.00410616 | 0.014077 |
| 7 | 7:124793437_A_G | 124793437 | 0.00048 | rs80299632 | A | G | 0.00378252 | -0.0126652 |
| 10 | 10:121169363_G_A | 121169363 | 0.00048 | rs10787966 | G | A | 0.00420916 | -0.014458 |
| 11 | 11:133101918_A_G | 133101918 | 0.00048 | rs12796255 | A | G | 0.00338177 | 0.01142 |
| 12 | 12:387451_G_A | 387451 | 0.00048 | 12:387451:G:A | G | A | 0.0034586 | -0.0125772 |
| 12 | 12:54436702_C_T | 54436702 | 0.00048 | rs137954489 | C | T | 0.0160399 | -0.0573098 |
| 13 | 13:62620778_G_A | 62620778 | 0.00048 | rs148216066 | G | A | 0.0160459 | 0.0551656 |
| 1 | 1:117018546_T_C | 117018546 | 0.00049 | rs17035776 | T | C | 0.00573675 | 0.0194263 |
| 2 | 2:11651522_A_G | 11651522 | 0.00049 | 2:11651522:A:G | A | G | 0.0107103 | 0.0371856 |
| 3 | 3:88019493_T_C | 88019493 | 0.00049 | rs12629984 | T | C | 0.00398484 | 0.0132038 |
| 4 | 4:91306116_A_G | 91306116 | 0.00049 | rs34027685 | A | G | 0.00335727 | -0.0118763 |
| 4 | 4:132941118_G_A | 132941118 | 0.00049 | rs143183692 | G | A | 0.0188702 | -0.0641495 |
| 6 | 6:55180945_G_T | 55180945 | 0.00049 | rs1604514 | G | T | 0.00481905 | 0.0170025 |
| 6 | 6:96572748_T_C | 96572748 | 0.00049 | rs11754559 | T | C | 0.00498182 | -0.0159202 |
| 7 | 7:36263068_G_A | 36263068 | 0.00049 | rs10281772 | G | A | 0.00468666 | 0.016166 |
| 7 | 7:69592701_G_T | 69592701 | 0.00049 | rs13228123 | G | T | 0.00371267 | -0.0129967 |
| 9 | 9:9624461_T_C | 9624461 | 0.00049 | rs142002412 | T | C | 0.0120292 | -0.0405292 |
| 10 | 10:12781832_C_T | 12781832 | 0.00049 | rs11817189 | C | T | 0.0101118 | 0.0328515 |
| 10 | 10:29405164_G_A | 29405164 | 0.00049 | rs78770713 | G | A | 0.0165871 | -0.0528757 |
| 13 | 13:114131954_C_T | 114131954 | 0.00049 | rs2257460 | C | T | 0.00941403 | -0.0330391 |
| 16 | 16:62478644_A_G | 62478644 | 0.00049 | rs1834080 | A | G | 0.00413306 | 0.0152185 |
| 16 | 16:72985981_T_C | 72985981 | 0.00049 | rs876444 | T | C | 0.00348476 | 0.012166 |
| 17 | 17:16543431_T_C | 16543431 | 0.00049 | rs9889590 | T | C | 0.00395354 | -0.0132569 |
| 3 | 3:18802882_T_C | 18802882 | 5E-04 | rs6550631 | T | C | 0.00334364 | -0.0123063 |
| 4 | 4:155508627_G_A | 155508627 | 5E-04 | rs2070018 | G | A | 0.0123871 | -0.0430946 |
| 4 | 4:170328804_A_G | 170328804 | 5E-04 | rs10023181 | A | G | 0.0047413 | -0.0163146 |
| 6 | 6:119105417_T_C | 119105417 | 5E-04 | rs17080617 | T | C | 0.00406653 | 0.0144512 |
| 7 | 7:44932538_C_A | 44932538 | 5E-04 | rs757694 | C | A | 0.00420555 | 0.0136333 |
| 7 | 7:71148011_T_C | 71148011 | 5E-04 | rs149148686 | T | C | 0.0120554 | -0.0415241 |
| 8 | 8:79959546_C_A | 79959546 | 5E-04 | rs80070666 | C | A | 0.0163357 | 0.0567169 |
| 9 | 9:104908320_C_T | 104908320 | 5E-04 | rs199871179 | C | T | 0.0114627 | -0.038828 |
| 12 | 12:38928472_T_C | 38928472 | 5E-04 | rs146294655 | T | C | 0.00724014 | -0.0260966 |
| 12 | 12:41385428_A_G | 41385428 | 5E-04 | rs157270 | A | G | 0.00552327 | -0.0197904 |
| 14 | 14:71139279_G_T | 71139279 | 5E-04 | rs8688 | G | T | 0.00336367 | -0.0120324 |
| 17 | 17:35798363_T_C | 35798363 | 5E-04 | rs3815073 | T | C | 0.00912527 | -0.0313676 |
| 17 | 17:60243377_G_A | 60243377 | 5E-04 | rs147720151 | G | A | 0.0105767 | 0.03758 |
| 1 | 1:7210552_A_G | 7210552 | 0.00051 | rs6693832 | A | G | 0.00354334 | 0.0112713 |
| 2 | 2:27336503_C_T | 27336503 | 0.00051 | rs12470752 | C | T | 0.00784765 | 0.0263864 |
| 3 | 3:151894094_T_G | 151894094 | 0.00051 | rs7617610 | T | G | 0.00505571 | 0.0176058 |
| 4 | 4:65128540_A_G | 65128540 | 0.00051 | rs28837783 | A | G | 0.00385699 | 0.0132248 |
| 4 | 4:92034092_A_G | 92034092 | 0.00051 | rs11732866 | A | G | 0.00368878 | -0.0134238 |
| 6 | 6:43268073_G_A | 43268073 | 0.00051 | rs3778491 | G | A | 0.00959906 | -0.0346736 |
| 6 | 6:135082532_G_A | 135082532 | 0.00051 | rs113353791 | G | A | 0.00886401 | -0.0327788 |
| 6 | 6:135395600_G_A | 135395600 | 0.00051 | rs145759340 | G | A | 0.0112252 | 0.0387852 |
| 8 | 8:29658117_C_T | 29658117 | 0.00051 | rs114839309 | C | T | 0.0151016 | 0.0546438 |
| 11 | 11:76271689_G_A | 76271689 | 0.00051 | rs2508747 | G | A | 0.00366484 | -0.0123365 |
| 12 | 12:31388199_C_T | 31388199 | 0.00051 | rs76475508 | C | T | 0.0119052 | -0.0400782 |
| 13 | 13:56886122_T_G | 56886122 | 0.00051 | rs530421465 | T | G | 0.0165071 | 0.0524458 |
| 14 | 14:27435381_G_A | 27435381 | 0.00051 | rs12882401 | G | A | 0.00365837 | -0.0128864 |
| 14 | 14:48112259_G_A | 48112259 | 0.00051 | rs150089314 | G | A | 0.00978011 | 0.0328847 |
| 18 | 18:25287953_G_A | 25287953 | 0.00051 | rs12606601 | G | A | 0.00667905 | -0.0224096 |
| 20 | 20:10067090_C_A | 10067090 | 0.00051 | rs507568 | C | A | 0.00333465 | -0.0112233 |
| 22 | 22:21032753_G_T | 21032753 | 0.00051 | rs56695765 | G | T | 0.00850498 | 0.0301325 |
| 2 | 2:44378535_G_T | 44378535 | 0.00052 | rs12612458 | G | T | 0.0043713 | -0.0154164 |
| 3 | 3:45995516_C_T | 45995516 | 0.00052 | rs148650293 | C | T | 0.0177187 | 0.064877 |
| 8 | 8:95286431_G_A | 95286431 | 0.00052 | rs2445719 | G | A | 0.00515616 | -0.01808 |
| 8 | 8:124765702_A_G | 124765702 | 0.00052 | rs10088262 | A | G | 0.00348304 | 0.0118488 |
| 9 | 9:27997339_C_T | 27997339 | 0.00052 | rs1197936 | C | T | 0.00355012 | 0.0120427 |
| 9 | 9:28190382_A_G | 28190382 | 0.00052 | 9:28190382:A:G | A | G | 0.0140976 | 0.0499192 |
| 11 | 11:12018221_G_A | 12018221 | 0.00052 | rs116973675 | G | A | 0.0121647 | 0.0388756 |
| 11 | 11:121754259_T_C | 121754259 | 0.00052 | rs140885381 | T | C | 0.0123744 | -0.0396878 |
| 13 | 13:41448879_C_T | 41448879 | 0.00052 | rs139156436 | C | T | 0.0160703 | 0.0524073 |
| 18 | 18:12346746_T_C | 12346746 | 0.00052 | rs12455273 | T | C | 0.00338377 | 0.0123588 |
| 20 | 20:10883131_A_G | 10883131 | 0.00052 | rs6040229 | A | G | 0.00352156 | 0.0116532 |
| 20 | 20:44507112_G_A | 44507112 | 0.00052 | rs139396693 | G | A | 0.0146766 | -0.0499101 |
| 1 | 1:2262487_A_G | 2262487 | 0.00053 | rs146620038 | A | G | 0.00866558 | 0.0291403 |
| 3 | 3:15358709_G_A | 15358709 | 0.00053 | rs2688662 | G | A | 0.00416057 | 0.0142729 |
| 4 | 4:11200718_C_T | 11200718 | 0.00053 | rs143609500 | C | T | 0.0150852 | -0.0508554 |
| 6 | 6:109715332_G_A | 109715332 | 0.00053 | rs77031799 | G | A | 0.00856649 | 0.028985 |
| 6 | 6:110015564_T_C | 110015564 | 0.00053 | rs141965670 | T | C | 0.0141837 | -0.0500687 |
| 11 | 11:127934975_C_T | 127934975 | 0.00053 | rs190507713 | C | T | 0.0159882 | -0.0535638 |
| 12 | 12:5031590_T_C | 5031590 | 0.00053 | rs4766312 | T | C | 0.00456762 | 0.015529 |
| 16 | 16:59041361_C_T | 59041361 | 0.00053 | rs1898365 | C | T | 0.00344373 | 0.0109159 |
| 18 | 18:54512510_G_A | 54512510 | 0.00053 | rs11659890 | G | A | 0.00406396 | 0.0135161 |
| 18 | 18:70676707_G_T | 70676707 | 0.00053 | 18:70676707:G:T | G | T | 0.0104443 | 0.0366232 |
| 20 | 20:46307295_C_T | 46307295 | 0.00053 | rs138191749 | C | T | 0.0114632 | 0.0405854 |
| 2 | 2:237124209_C_T | 237124209 | 0.00054 | rs6431439 | C | T | 0.00436457 | -0.0142831 |
| 4 | 4:75216597_A_G | 75216597 | 0.00054 | rs12507218 | A | G | 0.00338866 | -0.0125458 |
| 4 | 4:110473543_A_C | 110473543 | 0.00054 | 4:110473543:A:C | A | C | 0.0143549 | 0.0509332 |
| 5 | 5:44000542_G_A | 44000542 | 0.00054 | rs150481608 | G | A | 0.00716008 | 0.0245193 |
| 5 | 5:149679644_T_G | 149679644 | 0.00054 | rs12522210 | T | G | 0.0052929 | -0.0172058 |
| 6 | 6:28421567_C_T | 28421567 | 0.00054 | rs2859369 | C | T | 0.00372279 | -0.0136539 |
| 6 | 6:73530851_A_G | 73530851 | 0.00054 | rs10498884 | A | G | 0.0150429 | 0.0558486 |
| 6 | 6:110171982_C_T | 110171982 | 0.00054 | 6:110171982:C:T | C | T | 0.00367984 | 0.0117292 |
| 7 | 7:17445920_T_C | 17445920 | 0.00054 | rs118153701 | T | C | 0.0133748 | -0.0451559 |
| 9 | 9:37987297_G_A | 37987297 | 0.00054 | rs13298451 | G | A | 0.00360235 | -0.012672 |
| 10 | 10:131883514_C_T | 131883514 | 0.00054 | rs1072853 | C | T | 0.0034817 | 0.0126571 |
| 15 | 15:91227472_C_A | 91227472 | 0.00054 | rs34776072 | C | A | 0.00335891 | -0.0110878 |
| 17 | 17:60768846_T_C | 60768846 | 0.00054 | rs2460290 | T | C | 0.00955486 | 0.0330709 |
| 18 | 18:53166091_T_C | 53166091 | 0.00054 | rs117285364 | T | C | 0.0110842 | -0.0368094 |
| 22 | 22:23637664_A_G | 23637664 | 0.00054 | rs8138857 | A | G | 0.00440018 | 0.0146388 |
| 22 | 22:28714205_T_C | 28714205 | 0.00054 | rs6005757 | T | C | 0.00725228 | 0.0239203 |
| 1 | 1:92955426_G_A | 92955426 | 0.00055 | rs6703440 | G | A | 0.00726721 | 0.0258413 |
| 3 | 3:176568103_G_A | 176568103 | 0.00055 | rs6803362 | G | A | 0.00657214 | 0.023152 |
| 4 | 4:138464842_C_T | 138464842 | 0.00055 | rs7439567 | C | T | 0.003446 | -0.0121807 |
| 7 | 7:41273415_A_G | 41273415 | 0.00055 | rs2051870 | A | G | 0.00349139 | 0.0120035 |
| 10 | 10:110871992_C_T | 110871992 | 0.00055 | rs117391371 | C | T | 0.00943586 | 0.0328831 |
| 13 | 13:34793545_C_T | 34793545 | 0.00055 | rs11839186 | C | T | 0.00332258 | -0.0107521 |
| 13 | 13:69380036_G_A | 69380036 | 0.00055 | rs17570473 | G | A | 0.00995645 | -0.0329983 |
| 14 | 14:95370174_A_G | 95370174 | 0.00055 | rs1243562 | A | G | 0.00577672 | 0.019305 |
| 17 | 17:41223048_A_G | 41223048 | 0.00055 | rs4986854 | A | G | 0.0126054 | 0.0427472 |
| 1 | 1:174468818_G_T | 174468818 | 0.00056 | rs184606483 | G | T | 0.013012 | -0.0464818 |
| 2 | 2:242466139_C_T | 242466139 | 0.00056 | rs12617840 | C | T | 0.00346628 | 0.0134829 |
| 5 | 5:39847332_G_A | 39847332 | 0.00056 | rs117348317 | G | A | 0.00976222 | 0.03369 |
| 5 | 5:148442297_C_T | 148442297 | 0.00056 | 5:148442297:C:T | C | T | 0.00404318 | 0.0134134 |
| 6 | 6:112355662_G_A | 112355662 | 0.00056 | rs57367535 | G | A | 0.0045594 | -0.0161529 |
| 8 | 8:32433893_G_A | 32433893 | 0.00056 | rs2466072 | G | A | 0.00387237 | 0.0139699 |
| 10 | 10:63012852_T_C | 63012852 | 0.00056 | rs10821879 | T | C | 0.00375938 | 0.0124378 |
| 11 | 11:27679916_C_T | 27679916 | 0.00056 | rs6265 | C | T | 0.00338173 | 0.0117743 |
| 11 | 11:117293463_A_G | 117293463 | 0.00056 | rs11820562 | A | G | 0.00398549 | -0.0142221 |
| 14 | 14:53055536_G_T | 53055536 | 0.00056 | 14:53055536:G:T | G | T | 0.0159663 | 0.0550558 |
| 15 | 15:86855890_C_T | 86855890 | 0.00056 | rs117291394 | C | T | 0.0117487 | -0.0372204 |
| 16 | 16:56405970_A_G | 56405970 | 0.00056 | rs76240050 | A | G | 0.00900421 | -0.0297592 |
| 21 | 21:17661542_G_A | 17661542 | 0.00056 | rs2823740 | G | A | 0.0048924 | 0.0164453 |
| 3 | 3:64337275_A_G | 64337275 | 0.00057 | rs6782213 | A | G | 0.00334581 | 0.0118612 |
| 3 | 3:109957126_C_A | 109957126 | 0.00057 | rs12053963 | C | A | 0.00594823 | 0.0194016 |
| 3 | 3:149953618_T_C | 149953618 | 0.00057 | rs12634526 | T | C | 0.00467486 | -0.0157948 |
| 3 | 3:185616013_G_A | 185616013 | 0.00057 | rs139212114 | G | A | 0.0142999 | -0.0471064 |
| 3 | 3:186461216_G_A | 186461216 | 0.00057 | rs266760 | G | A | 0.00553453 | 0.0189774 |
| 4 | 4:120025002_C_T | 120025002 | 0.00057 | rs4261975 | C | T | 0.00444654 | 0.0155354 |
| 4 | 4:162776678_G_A | 162776678 | 0.00057 | rs7682544 | G | A | 0.0135219 | 0.0426259 |
| 4 | 4:180532288_T_C | 180532288 | 0.00057 | rs6818072 | T | C | 0.00980761 | 0.0367546 |
| 6 | 6:44205845_A_C | 44205845 | 0.00057 | 6:44205845:A:C | A | C | 0.00373193 | -0.012226 |
| 6 | 6:92561851_C_T | 92561851 | 0.00057 | rs117012857 | C | T | 0.0166765 | -0.0526528 |
| 9 | 9:137520936_T_C | 137520936 | 0.00057 | rs4304399 | T | C | 0.00369215 | -0.0129426 |
| 10 | 10:101342102_T_C | 101342102 | 0.00057 | rs4919355 | T | C | 0.00338077 | 0.0116367 |
| 13 | 13:96058682_G_A | 96058682 | 0.00057 | rs1538336 | G | A | 0.00441197 | 0.0150344 |
| 14 | 14:84314837_G_A | 84314837 | 0.00057 | rs191569528 | G | A | 0.0159814 | -0.0544399 |
| 15 | 15:100742786_G_A | 100742786 | 0.00057 | rs8035836 | G | A | 0.00333119 | 0.0101307 |
| 16 | 16:56596812_C_T | 56596812 | 0.00057 | rs71387120 | C | T | 0.00534904 | 0.0177042 |
| 19 | 19:31872738_C_A | 31872738 | 0.00057 | rs747730 | C | A | 0.0034855 | -0.0119205 |
| 20 | 20:31205771_T_G | 31205771 | 0.00057 | rs456798 | T | G | 0.00337843 | -0.0113012 |
| 4 | 4:77720527_C_T | 77720527 | 0.00058 | rs344104 | C | T | 0.0128247 | 0.0432146 |
| 5 | 5:57369117_A_G | 57369117 | 0.00058 | rs34739332 | A | G | 0.00407311 | 0.0140122 |
| 5 | 5:105905192_C_A | 105905192 | 0.00058 | rs140698269 | C | A | 0.0160377 | 0.0550685 |
| 6 | 6:6876129_A_G | 6876129 | 0.00058 | rs2326832 | A | G | 0.00398786 | -0.0131411 |
| 6 | 6:32339647_T_C | 32339647 | 0.00058 | 6:32339647:T:C | T | C | 0.00358761 | 0.0122939 |
| 6 | 6:97058567_A_G | 97058567 | 0.00058 | rs2273622 | A | G | 0.00513048 | -0.0170296 |
| 7 | 7:43173080_G_A | 43173080 | 0.00058 | rs1978232 | G | A | 0.00337459 | -0.0116791 |
| 12 | 12:125145655_A_C | 125145655 | 0.00058 | rs11057733 | A | C | 0.00852223 | -0.0287113 |
| 15 | 15:52742033_C_T | 52742033 | 0.00058 | rs1724588 | C | T | 0.00423013 | -0.0141755 |
| 16 | 16:20386115_T_C | 20386115 | 0.00058 | rs4522429 | T | C | 0.00346963 | 0.012364 |
| 16 | 16:81736928_G_A | 81736928 | 0.00058 | rs60309908 | G | A | 0.0044006 | -0.0142735 |
| 16 | 16:86100616_G_A | 86100616 | 0.00058 | rs76883775 | G | A | 0.00343997 | -0.0112391 |
| 19 | 19:6575726_G_A | 6575726 | 0.00058 | rs68037426 | G | A | 0.00929298 | 0.0332601 |
| 20 | 20:47258761_G_A | 47258761 | 0.00058 | rs78552395 | G | A | 0.00622818 | 0.0227966 |
| 1 | 1:1941828_T_C | 1941828 | 0.00059 | rs13303071 | T | C | 0.00348724 | 0.0124827 |
| 2 | 2:26812728_G_A | 26812728 | 0.00059 | rs79681689 | G | A | 0.00970107 | 0.0359425 |
| 3 | 3:20181615_G_T | 20181615 | 0.00059 | rs3762632 | G | T | 0.00341379 | 0.0112994 |
| 3 | 3:69357338_C_A | 69357338 | 0.00059 | 3:69357338:C:A | C | A | 0.00383143 | -0.0132864 |
| 4 | 4:171626347_G_T | 171626347 | 0.00059 | rs59512523 | G | T | 0.00520785 | 0.0186779 |
| 9 | 9:2529646_T_C | 2529646 | 0.00059 | rs10812204 | T | C | 0.00335844 | 0.0116208 |
| 10 | 10:74299146_T_G | 74299146 | 0.00059 | rs78099883 | T | G | 0.00986735 | -0.0328317 |
| 13 | 13:30695606_T_G | 30695606 | 0.00059 | rs482983 | T | G | 0.00355755 | 0.0117091 |
| 15 | 15:78873119_C_T | 78873119 | 0.00059 | rs569207 | C | T | 0.00332523 | 0.0117614 |
| 21 | 21:43686992_T_C | 43686992 | 0.00059 | rs225395 | T | C | 0.00333526 | 0.010904 |
| 1 | 1:11910677_A_G | 11910677 | 6E-04 | rs632793 | A | G | 0.00503889 | 0.0170439 |
| 1 | 1:188458988_C_T | 188458988 | 6E-04 | rs6656158 | C | T | 0.00361326 | -0.0122287 |
| 3 | 3:16639365_G_A | 16639365 | 6E-04 | rs75311389 | G | A | 0.0127646 | -0.043186 |
| 3 | 3:69644888_A_G | 69644888 | 6E-04 | rs73104189 | A | G | 0.00532993 | -0.0176411 |
| 4 | 4:75533892_A_G | 75533892 | 6E-04 | 4:75533892:A:G | A | G | 0.0155665 | 0.0548076 |
| 4 | 4:92655590_T_G | 92655590 | 6E-04 | rs11934075 | T | G | 0.00550194 | -0.0191409 |
| 5 | 5:5489383_G_A | 5489383 | 6E-04 | rs16900247 | G | A | 0.0037689 | 0.0132516 |
| 6 | 6:39855948_C_T | 39855948 | 6E-04 | rs74840002 | C | T | 0.0063144 | -0.0218415 |
| 6 | 6:55477277_A_G | 55477277 | 6E-04 | rs7750648 | A | G | 0.00449414 | 0.0151313 |
| 7 | 7:46079441_C_T | 46079441 | 6E-04 | rs187298771 | C | T | 0.0130727 | -0.0443791 |
| 7 | 7:153232739_A_C | 153232739 | 6E-04 | 7:153232739:A:C | A | C | 0.00458454 | -0.0147461 |
| 10 | 10:14614281_C_A | 14614281 | 6E-04 | rs10796203 | C | A | 0.00532577 | -0.0178469 |
| 10 | 10:105524081_C_T | 105524081 | 6E-04 | rs10883908 | C | T | 0.00350494 | -0.0122502 |
| 12 | 12:94964157_T_C | 94964157 | 6E-04 | rs1290005 | T | C | 0.00333215 | -0.0117519 |
| 14 | 14:21924225_G_A | 21924225 | 6E-04 | rs78484822 | G | A | 0.00717889 | 0.0242866 |
| 14 | 14:53115171_A_C | 53115171 | 6E-04 | rs11621600 | A | C | 0.0044391 | -0.0148177 |
| 1 | 1:29522674_C_T | 29522674 | 0.00061 | 1:29522674:C:T | C | T | 0.0158605 | 0.0558376 |
| 2 | 2:152900881_C_T | 152900881 | 0.00061 | rs4664522 | C | T | 0.0111747 | 0.0360081 |
| 3 | 3:114432826_T_C | 114432826 | 0.00061 | rs146361014 | T | C | 0.0137745 | -0.0495286 |
| 4 | 4:150006811_C_T | 150006811 | 0.00061 | rs181352268 | C | T | 0.0183086 | -0.0641957 |
| 5 | 5:91423022_T_G | 91423022 | 0.00061 | rs4421139 | T | G | 0.00333483 | -0.0101617 |
| 6 | 6:16180585_C_T | 16180585 | 0.00061 | 6:16180585:C:T | C | T | 0.0138317 | 0.0452091 |
| 8 | 8:74630299_C_T | 74630299 | 0.00061 | rs7814285 | C | T | 0.00336784 | 0.0121148 |
| 10 | 10:29008583_T_C | 29008583 | 0.00061 | rs2782389 | T | C | 0.00336316 | -0.0118079 |
| 10 | 10:59944373_A_G | 59944373 | 0.00061 | rs145897180 | A | G | 0.0130465 | 0.0473942 |
| 10 | 10:115082289_T_C | 115082289 | 0.00061 | rs541758 | T | C | 0.00333235 | 0.0114033 |
| 15 | 15:96216286_T_G | 96216286 | 0.00061 | rs1442414 | T | G | 0.00503961 | -0.0178854 |
| 16 | 16:8695530_G_A | 8695530 | 0.00061 | rs11644156 | G | A | 0.00448493 | 0.0153143 |
| 16 | 16:84465201_T_C | 84465201 | 0.00061 | rs12149038 | T | C | 0.00481328 | -0.0177758 |
| 18 | 18:47245456_C_A | 47245456 | 0.00061 | rs62101801 | C | A | 0.00514716 | 0.0175207 |
| 1 | 1:147102157_T_C | 147102157 | 0.00062 | rs2297961 | T | C | 0.00356914 | 0.0124428 |
| 3 | 3:14834850_T_C | 14834850 | 0.00062 | rs77095980 | T | C | 0.00974251 | -0.0355262 |
| 3 | 3:23734431_C_T | 23734431 | 0.00062 | rs4858529 | C | T | 0.00906545 | -0.0302824 |
| 7 | 7:69078855_T_C | 69078855 | 0.00062 | rs2533452 | T | C | 0.00373914 | 0.0128712 |
| 7 | 7:132855446_C_A | 132855446 | 0.00062 | rs185342672 | C | A | 0.0140595 | 0.0438682 |
| 11 | 11:69619875_T_C | 69619875 | 0.00062 | rs1893045 | T | C | 0.00342134 | 0.012157 |
| 13 | 13:89007252_C_T | 89007252 | 0.00062 | rs573269461 | C | T | 0.017861 | 0.0605298 |
| 15 | 15:73983013_C_T | 73983013 | 0.00062 | rs149289514 | C | T | 0.0122854 | -0.0412484 |
| 1 | 1:29805041_G_A | 29805041 | 0.00063 | rs1977332 | G | A | 0.00539704 | -0.0185013 |
| 2 | 2:146032359_A_G | 146032359 | 0.00063 | rs529020280 | A | G | 0.0170594 | -0.0562007 |
| 2 | 2:151120634_T_C | 151120634 | 0.00063 | rs187551655 | T | C | 0.0146154 | 0.0472024 |
| 3 | 3:184122152_T_C | 184122152 | 0.00063 | rs7648472 | T | C | 0.00404893 | -0.0137323 |
| 3 | 3:196329447_G_A | 196329447 | 0.00063 | rs148038338 | G | A | 0.0157355 | 0.0526766 |
| 5 | 5:96488207_C_T | 96488207 | 0.00063 | rs432482 | C | T | 0.00349437 | 0.011041 |
| 6 | 6:10430837_C_T | 10430837 | 0.00063 | rs72819713 | C | T | 0.0039005 | 0.0139777 |
| 6 | 6:23401124_T_C | 23401124 | 0.00063 | 6:23401124:T:C | T | C | 0.00367577 | -0.0126909 |
| 10 | 10:26815404_C_T | 26815404 | 0.00063 | rs10829016 | C | T | 0.00380584 | -0.013697 |
| 10 | 10:63495399_C_A | 63495399 | 0.00063 | rs189607322 | C | A | 0.0160948 | -0.0563034 |
| 10 | 10:104522633_G_A | 104522633 | 0.00063 | rs894609136 | G | A | 0.0147803 | -0.0507534 |
| 10 | 10:114835674_C_T | 114835674 | 0.00063 | rs61872795 | C | T | 0.00427613 | -0.0142911 |
| 11 | 11:44087715_T_G | 44087715 | 0.00063 | rs178513 | T | G | 0.00354182 | -0.011844 |
| 11 | 11:65460087_G_A | 65460087 | 0.00063 | rs72941018 | G | A | 0.00814168 | 0.0253256 |
| 15 | 15:42934631_A_C | 42934631 | 0.00063 | rs12594951 | A | C | 0.00337812 | -0.0114404 |
| 17 | 17:74144987_C_A | 74144987 | 0.00063 | rs150080490 | C | A | 0.0140555 | 0.046636 |
| 3 | 3:10112985_A_G | 10112985 | 0.00064 | rs117017234 | A | G | 0.00957827 | 0.0318585 |
| 4 | 4:55851739_T_C | 55851739 | 0.00064 | rs6554224 | T | C | 0.00439662 | -0.0148178 |
| 4 | 4:86794640_G_A | 86794640 | 0.00064 | rs141890665 | G | A | 0.0152129 | 0.0508665 |
| 5 | 5:76115521_C_T | 76115521 | 0.00064 | rs2242993 | C | T | 0.00735297 | 0.0250188 |
| 5 | 5:82989063_T_C | 82989063 | 0.00064 | rs146871287 | T | C | 0.015529 | 0.0564235 |
| 6 | 6:123343036_C_A | 123343036 | 0.00064 | rs141153710 | C | A | 0.0144883 | 0.0492178 |
| 7 | 7:12801874_C_T | 12801874 | 0.00064 | rs145735526 | C | T | 0.0149197 | -0.0499203 |
| 8 | 8:5999421_C_T | 5999421 | 0.00064 | rs2700714 | C | T | 0.00398981 | 0.0131395 |
| 8 | 8:107401202_T_C | 107401202 | 0.00064 | rs1954754 | T | C | 0.0033377 | 0.0116195 |
| 12 | 12:2550818_G_A | 2550818 | 0.00064 | rs2239079 | G | A | 0.00349459 | 0.0121508 |
| 12 | 12:2754073_G_A | 2754073 | 0.00064 | rs117339786 | G | A | 0.015018 | 0.0492336 |
| 15 | 15:66853975_C_T | 66853975 | 0.00064 | rs59153787 | C | T | 0.00526113 | -0.0181687 |
| 1 | 1:27945046_G_T | 27945046 | 0.00065 | rs75198513 | G | T | 0.0158159 | -0.051732 |
| 1 | 1:31821978_G_A | 31821978 | 0.00065 | rs144774588 | G | A | 0.0133573 | -0.0449484 |
| 2 | 2:28624161_C_T | 28624161 | 0.00065 | rs4666069 | C | T | 0.00379074 | -0.0130711 |
| 2 | 2:234289899_A_G | 234289899 | 0.00065 | rs78505555 | A | G | 0.00632755 | -0.022481 |
| 2 | 2:240162328_T_C | 240162328 | 0.00065 | rs189223779 | T | C | 0.0217905 | -0.07506 |
| 4 | 4:2821323_T_G | 2821323 | 0.00065 | rs6820967 | T | G | 0.00338557 | 0.010199 |
| 4 | 4:78937349_T_C | 78937349 | 0.00065 | rs13110474 | T | C | 0.00336926 | -0.0110724 |
| 9 | 9:118072970_C_A | 118072970 | 0.00065 | rs192629239 | C | A | 0.0141803 | -0.044693 |
| 12 | 12:91308906_G_A | 91308906 | 0.00065 | 12:91308906:G:A | G | A | 0.011329 | 0.0380218 |
| 13 | 13:20715801_C_A | 20715801 | 0.00065 | rs1886176 | C | A | 0.00366081 | 0.0122692 |
| 13 | 13:103955098_G_A | 103955098 | 0.00065 | 13:103955098:G:A | G | A | 0.00355775 | 0.0116699 |
| 16 | 16:413699_C_A | 413699 | 0.00065 | rs7201656 | C | A | 0.00499524 | -0.0150565 |
| 18 | 18:70954432_T_G | 70954432 | 0.00065 | rs73469404 | T | G | 0.0102804 | 0.0356723 |
| 20 | 20:38908605_A_G | 38908605 | 0.00065 | 20:38908605:A:G | A | G | 0.00412052 | -0.0133803 |
| 1 | 1:100739529_C_T | 100739529 | 0.00066 | rs79650351 | C | T | 0.00863439 | -0.0297943 |
| 2 | 2:191051980_A_G | 191051980 | 0.00066 | rs16832401 | A | G | 0.00599179 | -0.0210218 |
| 4 | 4:90837127_A_G | 90837127 | 0.00066 | rs10516850 | A | G | 0.00357598 | -0.012236 |
| 4 | 4:94458391_T_C | 94458391 | 0.00066 | rs1435473 | T | C | 0.00508552 | -0.0190392 |
| 5 | 5:155619741_T_C | 155619741 | 0.00066 | rs13152929 | T | C | 0.0147267 | -0.0505216 |
| 5 | 5:169833743_C_T | 169833743 | 0.00066 | rs560477664 | C | T | 0.0138159 | -0.0462069 |
| 6 | 6:31057386_A_G | 31057386 | 0.00066 | rs76409988 | A | G | 0.00419427 | 0.0143193 |
| 6 | 6:33044015_G_T | 33044015 | 0.00066 | rs115910061 | G | T | 0.00570404 | 0.0198456 |
| 6 | 6:107401043_T_C | 107401043 | 0.00066 | rs9373912 | T | C | 0.00383012 | 0.0136063 |
| 7 | 7:18544120_G_T | 18544120 | 0.00066 | rs573476045 | G | T | 0.0128113 | -0.0470575 |
| 7 | 7:29993208_T_G | 29993208 | 0.00066 | rs139852061 | T | G | 0.010107 | 0.0340271 |
| 9 | 9:127898603_T_G | 127898603 | 0.00066 | rs150910703 | T | G | 0.0103896 | -0.0348316 |
| 10 | 10:30078250_A_G | 30078250 | 0.00066 | rs150247349 | A | G | 0.00779536 | 0.0263491 |
| 10 | 10:96536708_C_T | 96536708 | 0.00066 | rs7068577 | C | T | 0.0173646 | 0.0602144 |
| 14 | 14:103772017_G_A | 103772017 | 0.00066 | rs78139593 | G | A | 0.0118922 | 0.0388594 |
| 15 | 15:46701546_G_A | 46701546 | 0.00066 | rs144759191 | G | A | 0.0167836 | 0.059871 |
| 15 | 15:98867809_T_C | 98867809 | 0.00066 | rs117401762 | T | C | 0.00812436 | -0.0285458 |
| 17 | 17:29914357_G_A | 29914357 | 0.00066 | rs72817744 | G | A | 0.0122191 | -0.0418815 |
| 18 | 18:352074_C_T | 352074 | 0.00066 | rs654312 | C | T | 0.00629812 | 0.0220807 |
| 2 | 2:154219966_T_C | 154219966 | 0.00067 | rs191938812 | T | C | 0.0164868 | -0.0579057 |
| 5 | 5:100715124_A_G | 100715124 | 0.00067 | rs168320 | A | G | 0.00644305 | 0.0217196 |
| 5 | 5:125132650_C_T | 125132650 | 0.00067 | rs79555437 | C | T | 0.00819011 | -0.0286538 |
| 7 | 7:29802292_A_G | 29802292 | 0.00067 | 7:29802292:A:G | A | G | 0.00346412 | 0.0112976 |
| 10 | 10:81032465_G_A | 81032465 | 0.00067 | rs10824737 | G | A | 0.00405873 | -0.0134553 |
| 11 | 11:76800127_A_G | 76800127 | 0.00067 | rs3824888 | A | G | 0.00410367 | 0.0137023 |
| 12 | 12:16469232_A_G | 16469232 | 0.00067 | rs374627259 | A | G | 0.0138004 | -0.0470516 |
| 15 | 15:90811290_C_T | 90811290 | 0.00067 | rs35144299 | C | T | 0.0103999 | 0.0346332 |
| 16 | 16:29900581_G_A | 29900581 | 0.00067 | rs150105015 | G | A | 0.00749968 | 0.0242919 |
| 17 | 17:27506007_G_A | 27506007 | 0.00067 | rs141128847 | G | A | 0.0119061 | 0.0367221 |
| 17 | 17:47529746_A_G | 47529746 | 0.00067 | rs2584681 | A | G | 0.00345519 | -0.0118371 |
| 20 | 20:18330108_G_A | 18330108 | 0.00067 | rs4814724 | G | A | 0.0033981 | 0.0111793 |
| 2 | 2:128588456_A_G | 128588456 | 0.00068 | rs3856368 | A | G | 0.00347815 | 0.0126314 |
| 2 | 2:166222718_A_G | 166222718 | 0.00068 | rs80185227 | A | G | 0.0101447 | 0.0351345 |
| 2 | 2:242250708_T_C | 242250708 | 0.00068 | rs76504488 | T | C | 0.018269 | 0.0617598 |
| 3 | 3:189409196_C_T | 189409196 | 0.00068 | rs2889918 | C | T | 0.00387774 | 0.0117002 |
| 4 | 4:22684429_A_G | 22684429 | 0.00068 | 4:22684429:A:G | A | G | 0.00432653 | -0.014048 |
| 7 | 7:158934102_G_A | 158934102 | 0.00068 | rs77003675 | G | A | 0.0079836 | -0.0281242 |
| 8 | 8:11480491_C_T | 11480491 | 0.00068 | rs75005830 | C | T | 0.00511716 | 0.0169458 |
| 9 | 9:15232884_T_C | 15232884 | 0.00068 | rs10961925 | T | C | 0.00366493 | 0.0127377 |
| 9 | 9:27870516_G_A | 27870516 | 0.00068 | rs10124928 | G | A | 0.0102316 | 0.0348715 |
| 11 | 11:108602717_G_T | 108602717 | 0.00068 | rs11212769 | G | T | 0.00393037 | 0.0131812 |
| 12 | 12:43215622_A_G | 43215622 | 0.00068 | rs10736005 | A | G | 0.00604792 | -0.0214711 |
| 12 | 12:109639521_G_A | 109639521 | 0.00068 | rs2284691 | G | A | 0.00504656 | -0.0185396 |
| 16 | 16:66723265_T_G | 66723265 | 0.00068 | rs118173521 | T | G | 0.00621738 | -0.021418 |
| 16 | 16:85974142_T_C | 85974142 | 0.00068 | rs305064 | T | C | 0.0048382 | 0.0172935 |
| 16 | 16:89717590_G_A | 89717590 | 0.00068 | rs78987932 | G | A | 0.00334711 | -0.0112404 |
| 17 | 17:72563599_T_C | 72563599 | 0.00068 | rs905708 | T | C | 0.00333849 | 0.0118123 |
| 19 | 19:39047428_A_C | 39047428 | 0.00068 | rs145101648 | A | C | 0.0145148 | -0.0463219 |
| 1 | 1:200481441_G_A | 200481441 | 0.00069 | rs148169002 | G | A | 0.0148404 | 0.0468505 |
| 2 | 2:163348190_C_T | 163348190 | 0.00069 | rs201761871 | C | T | 0.0177043 | -0.0651484 |
| 3 | 3:159267729_C_T | 159267729 | 0.00069 | rs74902805 | C | T | 0.0134549 | -0.0453818 |
| 3 | 3:191000349_C_T | 191000349 | 0.00069 | rs10937468 | C | T | 0.00335794 | 0.0111158 |
| 5 | 5:33202247_C_A | 33202247 | 0.00069 | rs11950850 | C | A | 0.00339606 | -0.0117778 |
| 5 | 5:107496102_T_C | 107496102 | 0.00069 | rs40071 | T | C | 0.00332443 | 0.0105473 |
| 7 | 7:149086688_A_C | 149086688 | 0.00069 | rs191017414 | A | C | 0.0177471 | -0.0604213 |
| 10 | 10:4713511_A_G | 4713511 | 0.00069 | rs144614474 | A | G | 0.0101995 | 0.0348226 |
| 10 | 10:125933307_G_A | 125933307 | 0.00069 | rs74750536 | G | A | 0.00463623 | -0.0165369 |
| 11 | 11:101203271_A_G | 101203271 | 0.00069 | rs149168058 | A | G | 0.0136449 | 0.0459283 |
| 12 | 12:111673176_G_A | 111673176 | 0.00069 | rs73197979 | G | A | 0.00938643 | -0.0290999 |
| 13 | 13:67370032_C_T | 67370032 | 0.00069 | 13:67370032:C:T | C | T | 0.00757594 | -0.0246327 |
| 13 | 13:94611749_A_G | 94611749 | 0.00069 | rs1933147 | A | G | 0.00337365 | 0.0104062 |
| 14 | 14:78131126_C_T | 78131126 | 0.00069 | rs117131659 | C | T | 0.00391928 | 0.0128598 |
| 18 | 18:63469053_T_C | 63469053 | 0.00069 | rs8092010 | T | C | 0.0097467 | -0.032101 |
| 22 | 22:28399915_C_T | 28399915 | 0.00069 | rs12160232 | C | T | 0.00764696 | 0.0247631 |
| 1 | 1:29092881_C_T | 29092881 | 7E-04 | rs11589088 | C | T | 0.0049021 | -0.0154333 |
| 2 | 2:54364368_C_T | 54364368 | 7E-04 | rs142769795 | C | T | 0.0121784 | -0.0390662 |
| 5 | 5:72593602_A_C | 72593602 | 7E-04 | rs7701037 | A | C | 0.00343065 | -0.0107613 |
| 5 | 5:79524541_C_T | 79524541 | 7E-04 | rs6882667 | C | T | 0.00342304 | -0.0111162 |
| 6 | 6:3789181_C_T | 3789181 | 7E-04 | rs9502072 | C | T | 0.00354376 | 0.011963 |
| 6 | 6:29555681_A_G | 29555681 | 7E-04 | 6:29555681:A:G | A | G | 0.00869152 | 0.0290101 |
| 6 | 6:107136274_A_G | 107136274 | 7E-04 | rs9398102 | A | G | 0.00450834 | -0.0149124 |
| 9 | 9:4191469_T_C | 4191469 | 7E-04 | rs62543613 | T | C | 0.0106744 | -0.0342532 |
| 9 | 9:90769805_G_A | 90769805 | 7E-04 | rs34946096 | G | A | 0.00385958 | 0.0135002 |
| 9 | 9:128783858_C_T | 128783858 | 7E-04 | rs79724509 | C | T | 0.0129054 | -0.0439307 |
| 9 | 9:131063476_G_A | 131063476 | 7E-04 | rs79321225 | G | A | 0.00631466 | 0.020766 |
| 10 | 10:104435950_C_T | 104435950 | 7E-04 | rs77129294 | C | T | 0.0144944 | -0.0488926 |
| 12 | 12:45291136_G_A | 45291136 | 7E-04 | rs2019452 | G | A | 0.00389705 | 0.0136031 |
| 12 | 12:52906153_A_G | 52906153 | 7E-04 | rs73103269 | A | G | 0.00541046 | 0.0182904 |
| 22 | 22:45824967_G_A | 45824967 | 7E-04 | rs138584719 | G | A | 0.015648 | -0.0550239 |
| 3 | 3:148728842_T_G | 148728842 | 0.00071 | rs3772573 | T | G | 0.00338116 | 0.0103743 |
| 4 | 4:107204681_G_A | 107204681 | 0.00071 | 4:107204681:G:A | G | A | 0.00654988 | 0.0232433 |
| 5 | 5:146867700_C_A | 146867700 | 0.00071 | rs726054 | C | A | 0.00491706 | -0.017988 |
| 6 | 6:32976909_C_A | 32976909 | 0.00071 | 6:32976909:C:A | C | A | 0.00337358 | 0.0115093 |
| 7 | 7:22609655_C_A | 22609655 | 0.00071 | rs1003924 | C | A | 0.00538838 | -0.0176095 |
| 8 | 8:126621187_C_T | 126621187 | 0.00071 | rs13259326 | C | T | 0.00739027 | -0.0252558 |
| 9 | 9:100971114_G_A | 100971114 | 0.00071 | rs41305493 | G | A | 0.0159206 | 0.0517135 |
| 11 | 11:110644532_T_C | 110644532 | 0.00071 | 11:110644532:T:C | T | C | 0.0033524 | -0.011232 |
| 13 | 13:41074466_G_A | 41074466 | 0.00071 | rs2802499 | G | A | 0.00463564 | 0.0163996 |
| 17 | 17:43304127_T_G | 43304127 | 0.00071 | rs148414437 | T | G | 0.0147433 | 0.049314 |
| 18 | 18:21563601_C_T | 21563601 | 0.00071 | rs56269021 | C | T | 0.00470211 | -0.0158537 |
| 19 | 19:58741666_C_T | 58741666 | 0.00071 | rs28693398 | C | T | 0.00874133 | -0.0291899 |
| 1 | 1:33132188_C_T | 33132188 | 0.00072 | rs786457 | C | T | 0.00411397 | 0.0137564 |
| 1 | 1:66672516_C_A | 66672516 | 0.00072 | rs57180329 | C | A | 0.012103 | -0.0410012 |
| 3 | 3:59977863_T_C | 59977863 | 0.00072 | rs79406655 | T | C | 0.0158112 | 0.0503257 |
| 6 | 6:151225565_G_A | 151225565 | 0.00072 | rs2295083 | G | A | 0.00421233 | -0.0145051 |
| 9 | 9:15208023_T_C | 15208023 | 0.00072 | rs10961915 | T | C | 0.00937376 | -0.0332543 |
| 10 | 10:104950627_A_G | 104950627 | 0.00072 | rs148824135 | A | G | 0.0144633 | 0.0509057 |
| 11 | 11:69346232_G_A | 69346232 | 0.00072 | rs11263479 | G | A | 0.00351969 | 0.0119683 |
| 13 | 13:31133133_C_T | 31133133 | 0.00072 | rs1331697 | C | T | 0.00359517 | -0.0125402 |
| 15 | 15:91399157_A_G | 91399157 | 0.00072 | rs193180540 | A | G | 0.0167261 | -0.0570376 |
| 19 | 19:23258711_T_C | 23258711 | 0.00072 | rs79671221 | T | C | 0.0162914 | 0.052423 |
| 1 | 1:78127554_T_C | 78127554 | 0.00073 | rs278852 | T | C | 0.00356485 | -0.0117179 |
| 3 | 3:10496681_T_C | 10496681 | 0.00073 | rs655553 | T | C | 0.00343625 | -0.0113303 |
| 4 | 4:31312965_C_T | 31312965 | 0.00073 | rs10033989 | C | T | 0.0154645 | 0.0490752 |
| 4 | 4:162487992_T_C | 162487992 | 0.00073 | 4:162487992:T:C | T | C | 0.0143294 | 0.0457914 |
| 6 | 6:136447203_A_G | 136447203 | 0.00073 | rs73565068 | A | G | 0.0102479 | 0.0336823 |
| 11 | 11:93374276_T_C | 93374276 | 0.00073 | rs4753485 | T | C | 0.00334866 | -0.0108944 |
| 11 | 11:116132415_T_C | 116132415 | 0.00073 | rs4938242 | T | C | 0.00697845 | 0.0221534 |
| 13 | 13:41168893_G_T | 41168893 | 0.00073 | rs184068690 | G | T | 0.017293 | 0.0563938 |
| 14 | 14:26160135_G_A | 26160135 | 0.00073 | rs141568371 | G | A | 0.01316 | 0.04371 |
| 2 | 2:36851264_C_T | 36851264 | 0.00074 | rs11124523 | C | T | 0.00418973 | -0.0142424 |
| 2 | 2:38759797_T_G | 38759797 | 0.00074 | rs13392800 | T | G | 0.00387095 | -0.0132076 |
| 2 | 2:56399153_T_C | 56399153 | 0.00074 | rs4671274 | T | C | 0.00372963 | 0.0117776 |
| 2 | 2:147474200_C_T | 147474200 | 0.00074 | rs148318987 | C | T | 0.0129016 | 0.0444225 |
| 3 | 3:124601972_C_T | 124601972 | 0.00074 | rs9968182 | C | T | 0.00342996 | 0.011394 |
| 4 | 4:25456590_T_C | 25456590 | 0.00074 | rs7660821 | T | C | 0.00359226 | 0.0121674 |
| 6 | 6:57299000_A_G | 57299000 | 0.00074 | rs758861 | A | G | 0.00367827 | -0.0125505 |
| 8 | 8:120402666_C_T | 120402666 | 0.00074 | rs150522964 | C | T | 0.0207979 | 0.0709145 |
| 8 | 8:138576650_G_A | 138576650 | 0.00074 | rs112210366 | G | A | 0.00368069 | 0.0120683 |
| 10 | 10:71606864_T_C | 71606864 | 0.00074 | rs10762316 | T | C | 0.00419859 | -0.0146316 |
| 16 | 16:53797792_C_T | 53797792 | 0.00074 | rs77722067 | C | T | 0.0150658 | -0.0484215 |
| 18 | 18:1224564_C_T | 1224564 | 0.00074 | rs7238392 | C | T | 0.004566 | 0.0151164 |
| 2 | 2:50197285_C_T | 50197285 | 0.00075 | rs17439328 | C | T | 0.0064012 | 0.0225122 |
| 2 | 2:227020853_G_A | 227020853 | 0.00075 | rs13388242 | G | A | 0.00412376 | 0.0140546 |
| 3 | 3:64789513_T_C | 64789513 | 0.00075 | 3:64789513:T:C | T | C | 0.0138017 | -0.0468664 |
| 3 | 3:72180966_T_C | 72180966 | 0.00075 | rs6779450 | T | C | 0.00467879 | 0.0157028 |
| 3 | 3:116774971_G_A | 116774971 | 0.00075 | rs11713439 | G | A | 0.00382083 | -0.012818 |
| 6 | 6:80318870_G_A | 80318870 | 0.00075 | rs75681002 | G | A | 0.0142035 | 0.0487653 |
| 10 | 10:90894233_T_G | 90894233 | 0.00075 | rs76175667 | T | G | 0.00553688 | 0.0188822 |
| 12 | 12:52305700_G_A | 52305700 | 0.00075 | rs12578436 | G | A | 0.00369107 | -0.0124152 |
| 12 | 12:122159954_G_A | 122159954 | 0.00075 | rs146058968 | G | A | 0.00850452 | -0.0297178 |
| 12 | 12:128753170_A_C | 128753170 | 0.00075 | rs145555263 | A | C | 0.00891278 | 0.0302979 |
| 14 | 14:61148304_T_C | 61148304 | 0.00075 | rs147332967 | T | C | 0.00932661 | 0.0292064 |
| 18 | 18:2938952_G_A | 2938952 | 0.00075 | rs79133417 | G | A | 0.0128354 | -0.0397259 |
| 18 | 18:56218354_C_A | 56218354 | 0.00075 | rs9797329 | C | A | 0.00423129 | -0.0143097 |
| 19 | 19:14910573_C_T | 14910573 | 0.00075 | rs10415562 | C | T | 0.00503209 | 0.0172239 |
| 19 | 19:33700445_C_T | 33700445 | 0.00075 | rs2303093 | C | T | 0.00661002 | -0.0223596 |
| 20 | 20:50061563_T_C | 50061563 | 0.00075 | rs228834 | T | C | 0.0150429 | -0.0476717 |
| 2 | 2:65661626_A_G | 65661626 | 0.00076 | rs12185613 | A | G | 0.00492499 | 0.0172658 |
| 2 | 2:146999990_G_A | 146999990 | 0.00076 | rs2381909 | G | A | 0.00375912 | -0.0128939 |
| 3 | 3:171081066_C_T | 171081066 | 0.00076 | rs141031906 | C | T | 0.011861 | 0.0413606 |
| 4 | 4:188892958_T_G | 188892958 | 0.00076 | rs4861762 | T | G | 0.00333337 | 0.0113926 |
| 5 | 5:137653737_G_A | 137653737 | 0.00076 | rs150416645 | G | A | 0.0156313 | 0.0490463 |
| 6 | 6:10878935_C_T | 10878935 | 0.00076 | rs190765395 | C | T | 0.0127716 | -0.0436526 |
| 6 | 6:54615614_G_A | 54615614 | 0.00076 | rs140050930 | G | A | 0.013639 | -0.0456378 |
| 9 | 9:9789202_C_T | 9789202 | 0.00076 | rs17232558 | C | T | 0.00675447 | -0.0221891 |
| 10 | 10:21257019_G_A | 21257019 | 0.00076 | rs77428179 | G | A | 0.00362724 | 0.0116509 |
| 10 | 10:105161480_C_T | 105161480 | 0.00076 | rs186833856 | C | T | 0.0152668 | -0.0498838 |
| 11 | 11:33759191_T_C | 33759191 | 0.00076 | rs79077373 | T | C | 0.0103383 | 0.0333026 |
| 13 | 13:24627728_G_A | 24627728 | 0.00076 | rs1113414 | G | A | 0.00339663 | -0.0108847 |
| 2 | 2:104010742_G_T | 104010742 | 0.00077 | 2:104010742:G:T | G | T | 0.0112189 | -0.037516 |
| 2 | 2:193240018_C_T | 193240018 | 0.00077 | rs77826353 | C | T | 0.0107029 | -0.036701 |
| 4 | 4:139889486_T_G | 139889486 | 0.00077 | rs62320504 | T | G | 0.00374088 | -0.0121705 |
| 8 | 8:79442653_C_A | 79442653 | 0.00077 | rs77743907 | C | A | 0.0176733 | 0.0580268 |
| 9 | 9:118389745_T_C | 118389745 | 0.00077 | rs75964829 | T | C | 0.00617107 | 0.0199256 |
| 13 | 13:87109655_C_T | 87109655 | 0.00077 | rs77760987 | C | T | 0.0155025 | 0.050455 |
| 21 | 21:42875620_G_A | 42875620 | 0.00077 | rs146120690 | G | A | 0.0139624 | 0.0476809 |
| 1 | 1:210916543_G_A | 210916543 | 0.00078 | rs199773200 | G | A | 0.0115396 | -0.0406245 |
| 2 | 2:167224823_T_C | 167224823 | 0.00078 | rs6714902 | T | C | 0.00455563 | 0.0162384 |
| 6 | 6:137714855_G_A | 137714855 | 0.00078 | rs10457021 | G | A | 0.00383983 | 0.0127431 |
| 8 | 8:71487638_G_A | 71487638 | 0.00078 | rs575807811 | G | A | 0.0169532 | 0.0561887 |
| 10 | 10:77140864_A_G | 77140864 | 0.00078 | rs7078507 | A | G | 0.00332528 | 0.0109678 |
| 12 | 12:564238_G_A | 564238 | 0.00078 | rs35184040 | G | A | 0.00500165 | -0.0163244 |
| 12 | 12:107772567_T_C | 107772567 | 0.00078 | rs1426466 | T | C | 0.00341656 | 0.0109589 |
| 16 | 16:56624079_A_G | 56624079 | 0.00078 | rs11644094 | A | G | 0.00462708 | -0.0158123 |
| 19 | 19:29501984_C_T | 29501984 | 0.00078 | rs117461915 | C | T | 0.0158153 | -0.0556197 |
| 2 | 2:108800584_T_C | 108800584 | 0.00079 | rs860587 | T | C | 0.00524571 | -0.0175257 |
| 2 | 2:139168700_T_C | 139168700 | 0.00079 | rs187174397 | T | C | 0.0159215 | -0.0554684 |
| 4 | 4:110815537_G_A | 110815537 | 0.00079 | rs202052463 | G | A | 0.0100515 | 0.0319079 |
| 5 | 5:158462918_A_G | 158462918 | 0.00079 | rs1363561 | A | G | 0.00376931 | 0.0127924 |
| 6 | 6:33927408_G_T | 33927408 | 0.00079 | rs2495969 | G | T | 0.00417622 | -0.0133954 |
| 7 | 7:83874600_G_A | 83874600 | 0.00079 | rs56231816 | G | A | 0.0124853 | -0.0414725 |
| 8 | 8:136217890_G_T | 136217890 | 0.00079 | rs113777497 | G | T | 0.00491091 | -0.016676 |
| 10 | 10:18255764_G_A | 18255764 | 0.00079 | 10:18255764:G:A | G | A | 0.013181 | -0.0436872 |
| 11 | 11:120303763_C_T | 120303763 | 0.00079 | rs117503416 | C | T | 0.00634569 | 0.0229281 |
| 12 | 12:103116187_C_T | 103116187 | 0.00079 | rs6539050 | C | T | 0.00460546 | -0.0159861 |
| 15 | 15:23944385_G_A | 23944385 | 0.00079 | rs1100803 | G | A | 0.00614319 | 0.0192021 |
| 15 | 15:47468691_A_G | 47468691 | 0.00079 | rs117449441 | A | G | 0.0148256 | 0.0479878 |
| 18 | 18:64848183_G_T | 64848183 | 0.00079 | rs11663828 | G | T | 0.0039309 | -0.0131185 |
| 19 | 19:11256285_A_G | 11256285 | 0.00079 | rs4804147 | A | G | 0.00459716 | 0.0158671 |
| 20 | 20:10971744_T_C | 10971744 | 0.00079 | rs7273599 | T | C | 0.00384001 | -0.0142443 |
| 1 | 1:69816251_G_T | 69816251 | 8E-04 | rs139237119 | G | T | 0.0147345 | 0.0525892 |
| 2 | 2:145744975_A_G | 145744975 | 8E-04 | rs186983974 | A | G | 0.018042 | -0.0590464 |
| 2 | 2:205089262_A_G | 205089262 | 8E-04 | rs16840902 | A | G | 0.00482111 | 0.0162115 |
| 3 | 3:143576296_C_T | 143576296 | 8E-04 | rs146140094 | C | T | 0.0151465 | -0.0530534 |
| 5 | 5:2194769_C_A | 2194769 | 8E-04 | rs16903612 | C | A | 0.00818712 | 0.0257958 |
| 5 | 5:103806932_T_C | 103806932 | 8E-04 | 5:103806932:T:C | T | C | 0.00373102 | 0.0124276 |
| 7 | 7:65822891_T_C | 65822891 | 8E-04 | rs536543205 | T | C | 0.0133545 | -0.0439977 |
| 10 | 10:46018831_A_G | 46018831 | 8E-04 | rs11239549 | A | G | 0.00608592 | 0.0206034 |
| 10 | 10:122901341_G_A | 122901341 | 8E-04 | rs1907246 | G | A | 0.00403614 | -0.0134295 |
| 15 | 15:44060615_C_T | 44060615 | 8E-04 | rs117062566 | C | T | 0.00860924 | 0.0282564 |
| 17 | 17:17998893_T_C | 17998893 | 8E-04 | rs138045057 | T | C | 0.00563005 | -0.0180935 |
| 20 | 20:59688743_G_T | 59688743 | 8E-04 | rs8183106 | G | T | 0.00347106 | -0.012213 |
| 1 | 1:174735278_C_T | 174735278 | 0.00081 | rs181197814 | C | T | 0.0135089 | -0.0469896 |
| 2 | 2:19955205_T_C | 19955205 | 0.00081 | rs1005943 | T | C | 0.005412 | 0.0174425 |
| 3 | 3:53438283_T_C | 53438283 | 0.00081 | rs147453806 | T | C | 0.0099546 | -0.0342126 |
| 3 | 3:127438760_G_T | 127438760 | 0.00081 | rs78621606 | G | T | 0.017233 | -0.0564547 |
| 4 | 4:55329649_A_G | 55329649 | 0.00081 | rs182824454 | A | G | 0.0156513 | 0.0561229 |
| 6 | 6:97621320_C_T | 97621320 | 0.00081 | rs1623056 | C | T | 0.00386527 | 0.0125087 |
| 6 | 6:116665082_A_C | 116665082 | 0.00081 | rs575112144 | A | C | 0.0117933 | 0.0399654 |
| 7 | 7:46008110_G_A | 46008110 | 0.00081 | rs11977526 | G | A | 0.00397769 | 0.0137346 |
| 10 | 10:18409845_A_G | 18409845 | 0.00081 | rs80255112 | A | G | 0.00434376 | 0.0143245 |
| 11 | 11:128109120_T_C | 128109120 | 0.00081 | rs471557 | T | C | 0.00403189 | -0.0139886 |
| 12 | 12:17196772_A_G | 17196772 | 0.00081 | rs7313107 | A | G | 0.00361891 | 0.0118615 |
| 13 | 13:48051775_G_A | 48051775 | 0.00081 | rs147713791 | G | A | 0.00854164 | -0.0295654 |
| 16 | 16:26987229_C_T | 26987229 | 0.00081 | rs186688055 | C | T | 0.00930211 | -0.0305463 |
| 1 | 1:156095359_A_C | 156095359 | 0.00082 | rs142909110 | A | C | 0.0102208 | -0.031143 |
| 2 | 2:73553397_G_A | 73553397 | 0.00082 | rs6546820 | G | A | 0.00371716 | -0.0124408 |
| 2 | 2:183193783_T_C | 183193783 | 0.00082 | rs140973866 | T | C | 0.00652475 | -0.0206982 |
| 3 | 3:125087678_C_T | 125087678 | 0.00082 | rs11719535 | C | T | 0.00334458 | -0.0111368 |
| 4 | 4:57007798_A_G | 57007798 | 0.00082 | rs13112186 | A | G | 0.00909663 | -0.0336132 |
| 4 | 4:121355432_G_A | 121355432 | 0.00082 | rs705104 | G | A | 0.0037897 | 0.0128904 |
| 9 | 9:24837993_G_A | 24837993 | 0.00082 | rs118041334 | G | A | 0.0151952 | 0.0513193 |
| 9 | 9:34381192_C_A | 34381192 | 0.00082 | rs3753033 | C | A | 0.00356302 | 0.0119404 |
| 12 | 12:110233540_T_C | 110233540 | 0.00082 | rs12579162 | T | C | 0.00635448 | -0.022101 |
| 14 | 14:22022946_G_T | 22022946 | 0.00082 | rs11846361 | G | T | 0.00339419 | -0.0118031 |
| 20 | 20:14688777_T_C | 14688777 | 0.00082 | rs11699301 | T | C | 0.00354795 | 0.0112778 |
| 1 | 1:115450844_T_G | 115450844 | 0.00083 | rs78255367 | T | G | 0.00914785 | -0.0294713 |
| 2 | 2:29544320_C_T | 29544320 | 0.00083 | rs78911101 | C | T | 0.0139713 | 0.0466176 |
| 3 | 3:53992944_T_C | 53992944 | 0.00083 | rs78887650 | T | C | 0.00541465 | 0.0177551 |
| 4 | 4:26808829_C_T | 26808829 | 0.00083 | rs7687234 | C | T | 0.00342756 | -0.0117532 |
| 4 | 4:137408872_T_C | 137408872 | 0.00083 | rs6535149 | T | C | 0.00335116 | -0.0116225 |
| 4 | 4:170619885_T_C | 170619885 | 0.00083 | rs77661390 | T | C | 0.00558579 | 0.0187506 |
| 5 | 5:127733961_A_G | 127733961 | 0.00083 | rs74989671 | A | G | 0.00435721 | -0.0146148 |
| 6 | 6:27455138_G_A | 27455138 | 0.00083 | rs6456793 | G | A | 0.00363611 | 0.0122641 |
| 6 | 6:30529816_G_A | 30529816 | 0.00083 | rs3888777 | G | A | 0.00717619 | 0.0240301 |
| 6 | 6:150325427_G_T | 150325427 | 0.00083 | 6:150325427:G:T | G | T | 0.00714953 | -0.0248225 |
| 7 | 7:28090868_T_C | 28090868 | 0.00083 | rs7778929 | T | C | 0.0034146 | 0.0107362 |
| 8 | 8:56867618_A_C | 56867618 | 0.00083 | rs10100561 | A | C | 0.0036643 | 0.0124691 |
| 9 | 9:97673069_G_A | 97673069 | 0.00083 | 9:97673069:G:A | G | A | 0.00486703 | -0.0158576 |
| 9 | 9:111696214_A_C | 111696214 | 0.00083 | 9:111696214:A:C | A | C | 0.00499957 | -0.0167258 |
| 11 | 11:32220283_T_C | 32220283 | 0.00083 | rs988233 | T | C | 0.00334363 | -0.0116494 |
| 12 | 12:116198214_G_A | 116198214 | 0.00083 | rs11067762 | G | A | 0.00361121 | 0.0121837 |
| 13 | 13:78765391_G_T | 78765391 | 0.00083 | rs74895487 | G | T | 0.00979229 | 0.0328085 |
| 15 | 15:27402601_C_T | 27402601 | 0.00083 | rs2286945 | C | T | 0.0036467 | 0.0126683 |
| 1 | 1:69503724_G_A | 69503724 | 0.00084 | 1:69503724:G:A | G | A | 0.00937741 | -0.0314311 |
| 2 | 2:102314437_T_C | 102314437 | 0.00084 | rs13430711 | T | C | 0.00389874 | 0.012421 |
| 2 | 2:165213501_G_A | 165213501 | 0.00084 | rs13032768 | G | A | 0.00363113 | 0.0129543 |
| 2 | 2:228697588_G_A | 228697588 | 0.00084 | rs79465534 | G | A | 0.0152449 | -0.0513239 |
| 3 | 3:5522970_A_G | 5522970 | 0.00084 | rs9814798 | A | G | 0.00378552 | -0.0117618 |
| 6 | 6:27291379_A_G | 27291379 | 0.00084 | rs117993336 | A | G | 0.00948583 | -0.0320073 |
| 7 | 7:118672913_C_T | 118672913 | 0.00084 | rs201344677 | C | T | 0.0164731 | -0.0537628 |
| 7 | 7:138141079_C_A | 138141079 | 0.00084 | rs112767772 | C | A | 0.0085113 | -0.0261183 |
| 8 | 8:39850822_G_A | 39850822 | 0.00084 | rs78080531 | G | A | 0.00911287 | -0.030244 |
| 9 | 9:26222607_C_T | 26222607 | 0.00084 | rs10812371 | C | T | 0.00365058 | -0.01248 |
| 9 | 9:128042293_C_T | 128042293 | 0.00084 | rs188049072 | C | T | 0.00934331 | -0.0289232 |
| 13 | 13:102691029_C_A | 102691029 | 0.00084 | 13:102691029:C:A | C | A | 0.013438 | -0.0442371 |
| 13 | 13:113629721_A_G | 113629721 | 0.00084 | rs2993274 | A | G | 0.00335979 | 0.0112004 |
| 14 | 14:27087018_T_C | 27087018 | 0.00084 | rs79170341 | T | C | 0.00943531 | 0.0301715 |
| 14 | 14:47909206_T_G | 47909206 | 0.00084 | rs2416078 | T | G | 0.00335229 | 0.0103874 |
| 14 | 14:60304119_T_C | 60304119 | 0.00084 | rs1957306 | T | C | 0.00338842 | -0.010887 |
| 14 | 14:60672662_C_T | 60672662 | 0.00084 | rs140096195 | C | T | 0.00797488 | 0.0260159 |
| 15 | 15:26114658_C_T | 26114658 | 0.00084 | rs1553893 | C | T | 0.00559484 | 0.0198525 |
| 15 | 15:92731487_G_A | 92731487 | 0.00084 | rs11074045 | G | A | 0.00450891 | -0.0152625 |
| 17 | 17:48979829_A_G | 48979829 | 0.00084 | rs140953255 | A | G | 0.01139 | -0.0392821 |
| 17 | 17:66108706_A_G | 66108706 | 0.00084 | rs62085664 | A | G | 0.00370574 | -0.0123823 |
| 18 | 18:38319135_G_A | 38319135 | 0.00084 | rs12606988 | G | A | 0.00636052 | -0.0214154 |
| 20 | 20:54535621_G_A | 54535621 | 0.00084 | rs4811646 | G | A | 0.0038826 | -0.0135869 |
| 2 | 2:152475938_T_G | 152475938 | 0.00085 | 2:152475938:T:G | T | G | 0.00384625 | -0.0126111 |
| 2 | 2:172159930_G_A | 172159930 | 0.00085 | rs117068099 | G | A | 0.010626 | -0.0342314 |
| 3 | 3:27222419_A_G | 27222419 | 0.00085 | rs9875517 | A | G | 0.00796149 | -0.0273308 |
| 3 | 3:60099156_A_G | 60099156 | 0.00085 | rs760317 | A | G | 0.00354797 | 0.0122684 |
| 3 | 3:138974704_G_A | 138974704 | 0.00085 | 3:138974704:G:A | G | A | 0.00336298 | 0.0112133 |
| 4 | 4:134692335_G_A | 134692335 | 0.00085 | rs6831760 | G | A | 0.00359077 | 0.0122688 |
| 5 | 5:101720384_T_C | 101720384 | 0.00085 | rs143375273 | T | C | 0.0134926 | 0.0423553 |
| 6 | 6:28012471_G_A | 28012471 | 0.00085 | rs77094101 | G | A | 0.0132196 | -0.0423813 |
| 7 | 7:18795250_T_G | 18795250 | 0.00085 | rs17139817 | T | G | 0.00337677 | -0.0112502 |
| 9 | 9:648690_G_A | 648690 | 0.00085 | rs147310732 | G | A | 0.0166954 | 0.0537589 |
| 9 | 9:21746419_T_C | 21746419 | 0.00085 | rs10757244 | T | C | 0.0035102 | -0.0115306 |
| 10 | 10:69410048_G_A | 69410048 | 0.00085 | rs10430513 | G | A | 0.0037655 | -0.013041 |
| 10 | 10:96563757_G_A | 96563757 | 0.00085 | rs4494250 | G | A | 0.00403089 | -0.0135601 |
| 11 | 11:80637478_T_C | 80637478 | 0.00085 | rs73508954 | T | C | 0.00688012 | 0.0234403 |
| 13 | 13:60883878_C_T | 60883878 | 0.00085 | rs75508903 | C | T | 0.00903566 | -0.0302169 |
| 13 | 13:102685934_C_T | 102685934 | 0.00085 | rs1075956 | C | T | 0.0106245 | -0.03588 |
| 15 | 15:89294227_C_T | 89294227 | 0.00085 | rs77047412 | C | T | 0.00587717 | -0.0203451 |
| 2 | 2:157602842_T_C | 157602842 | 0.00086 | rs79162832 | T | C | 0.00490232 | 0.0161666 |
| 3 | 3:72556335_G_A | 72556335 | 0.00086 | rs4494866 | G | A | 0.00334747 | 0.0112418 |
| 3 | 3:85737788_T_G | 85737788 | 0.00086 | rs6805113 | T | G | 0.0034819 | -0.0114143 |
| 4 | 4:61662306_G_A | 61662306 | 0.00086 | rs4623038 | G | A | 0.00393401 | -0.0132993 |
| 5 | 5:50900360_G_A | 50900360 | 0.00086 | rs76482666 | G | A | 0.00491686 | 0.0168195 |
| 5 | 5:163104597_G_A | 163104597 | 0.00086 | rs493108 | G | A | 0.00377815 | -0.0122344 |
| 6 | 6:38684867_A_G | 38684867 | 0.00086 | rs115843619 | A | G | 0.0100438 | -0.0324076 |
| 6 | 6:90978829_G_A | 90978829 | 0.00086 | rs618371 | G | A | 0.00343075 | 0.0107898 |
| 7 | 7:68652464_A_G | 68652464 | 0.00086 | rs142643441 | A | G | 0.0111501 | 0.0366475 |
| 7 | 7:95036897_A_G | 95036897 | 0.00086 | rs9640633 | A | G | 0.00599793 | -0.0193676 |
| 8 | 8:72781845_A_G | 72781845 | 0.00086 | rs183654229 | A | G | 0.016655 | -0.0526991 |
| 10 | 10:96143498_G_A | 96143498 | 0.00086 | rs11187919 | G | A | 0.017541 | 0.0598348 |
| 10 | 10:96932449_T_C | 96932449 | 0.00086 | rs138428477 | T | C | 0.0168099 | -0.0527224 |
| 15 | 15:52546520_T_C | 52546520 | 0.00086 | rs4627279 | T | C | 0.00367923 | -0.0119828 |
| 17 | 17:13180747_C_T | 13180747 | 0.00086 | rs12947402 | C | T | 0.00473775 | 0.0166524 |
| 17 | 17:38030041_T_C | 38030041 | 0.00086 | rs76599500 | T | C | 0.0108737 | 0.0347505 |
| 22 | 22:40479448_G_A | 40479448 | 0.00086 | rs7292838 | G | A | 0.00563214 | 0.0180057 |
| 1 | 1:238955230_A_G | 238955230 | 0.00087 | rs2689190 | A | G | 0.00345555 | -0.0109339 |
| 2 | 2:50682462_C_T | 50682462 | 0.00087 | rs1005431 | C | T | 0.00359966 | -0.0116431 |
| 2 | 2:183696219_T_G | 183696219 | 0.00087 | rs288318 | T | G | 0.00532392 | -0.0185928 |
| 5 | 5:61834319_C_T | 61834319 | 0.00087 | rs145626028 | C | T | 0.0132359 | -0.0415676 |
| 5 | 5:73259609_C_T | 73259609 | 0.00087 | rs17733586 | C | T | 0.00378546 | 0.0121541 |
| 6 | 6:26125342_C_T | 26125342 | 0.00087 | rs129128 | C | T | 0.00992559 | 0.0333017 |
| 6 | 6:97846871_A_C | 97846871 | 0.00087 | rs117404563 | A | C | 0.0169596 | -0.0559965 |
| 10 | 10:21043769_C_T | 21043769 | 0.00087 | rs376434913 | C | T | 0.00967275 | -0.0317363 |
| 10 | 10:125213800_A_C | 125213800 | 0.00087 | rs845086 | A | C | 0.00333143 | -0.0113562 |
| 13 | 13:41320577_T_C | 41320577 | 0.00087 | rs150239454 | T | C | 0.0146599 | 0.045442 |
| 14 | 14:81283948_C_T | 81283948 | 0.00087 | rs117898136 | C | T | 0.00750152 | -0.0269249 |
| 17 | 17:62479273_A_C | 62479273 | 0.00087 | rs17650301 | A | C | 0.0037153 | 0.0129373 |
| 18 | 18:2309411_C_T | 2309411 | 0.00087 | rs76149732 | C | T | 0.00935361 | 0.0301422 |
| 1 | 1:204457700_C_A | 204457700 | 0.00088 | rs138441041 | C | A | 0.0170041 | 0.0607267 |
| 2 | 2:55217518_T_C | 55217518 | 0.00088 | rs75903234 | T | C | 0.0045535 | -0.0141305 |
| 2 | 2:235473337_T_C | 235473337 | 0.00088 | rs12617228 | T | C | 0.00540981 | -0.0184417 |
| 3 | 3:23229563_G_A | 23229563 | 0.00088 | rs77563455 | G | A | 0.00986665 | -0.0312545 |
| 4 | 4:157654717_A_G | 157654717 | 0.00088 | rs4292374 | A | G | 0.00345565 | 0.0122827 |
| 5 | 5:19531541_G_A | 19531541 | 0.00088 | 5:19531541:G:A | G | A | 0.00695442 | 0.0213878 |
| 5 | 5:86964642_T_G | 86964642 | 0.00088 | rs189186040 | T | G | 0.0160569 | -0.0531107 |
| 7 | 7:100761408_G_A | 100761408 | 0.00088 | rs142207813 | G | A | 0.0145724 | -0.050394 |
| 8 | 8:54023170_G_A | 54023170 | 0.00088 | rs147197769 | G | A | 0.00781528 | -0.0275459 |
| 10 | 10:60287700_A_C | 60287700 | 0.00088 | rs117353586 | A | C | 0.00972525 | -0.0333431 |
| 11 | 11:44133486_A_G | 44133486 | 0.00088 | rs141524494 | A | G | 0.0111724 | 0.0372716 |
| 13 | 13:72624605_T_C | 72624605 | 0.00088 | rs118170601 | T | C | 0.0187835 | 0.0618415 |
| 14 | 14:74681228_G_A | 74681228 | 0.00088 | rs8013320 | G | A | 0.00336218 | -0.011172 |
| 14 | 14:77291214_G_T | 77291214 | 0.00088 | rs74068872 | G | T | 0.00340613 | -0.0110955 |
| 16 | 16:84448498_C_T | 84448498 | 0.00088 | 16:84448498:C:T | C | T | 0.0101654 | 0.0348955 |
| 20 | 20:9989696_G_A | 9989696 | 0.00088 | rs7343217 | G | A | 0.00375668 | 0.0125722 |
| 22 | 22:28263475_G_A | 28263475 | 0.00088 | rs74467157 | G | A | 0.0119062 | -0.0397313 |
| 1 | 1:155505967_G_A | 155505967 | 0.00089 | rs12746592 | G | A | 0.0039039 | 0.0125679 |
| 3 | 3:146368187_C_T | 146368187 | 0.00089 | rs9842222 | C | T | 0.00462342 | -0.0158829 |
| 5 | 5:52368473_G_A | 52368473 | 0.00089 | rs118190231 | G | A | 0.00840135 | 0.0275209 |
| 5 | 5:124630921_A_G | 124630921 | 0.00089 | rs11747033 | A | G | 0.00361857 | 0.0117109 |
| 6 | 6:162303953_C_T | 162303953 | 0.00089 | rs146376043 | C | T | 0.0123062 | 0.0413394 |
| 8 | 8:91800104_G_A | 91800104 | 0.00089 | rs180844417 | G | A | 0.0165625 | 0.0549794 |
| 9 | 9:10933694_A_C | 10933694 | 0.00089 | rs143891150 | A | C | 0.017239 | -0.0568707 |
| 12 | 12:97925364_T_G | 97925364 | 0.00089 | rs59066241 | T | G | 0.00370616 | -0.0125952 |
| 13 | 13:47292772_A_G | 47292772 | 0.00089 | rs141445308 | A | G | 0.0180921 | -0.0589028 |
| 15 | 15:96964299_G_A | 96964299 | 0.00089 | rs62009324 | G | A | 0.00852435 | 0.0306385 |
| 18 | 18:14342923_G_A | 14342923 | 0.00089 | rs4797905 | G | A | 0.00355016 | -0.0107942 |
| 18 | 18:66258965_A_G | 66258965 | 0.00089 | rs579814 | A | G | 0.0146706 | -0.0471655 |
| 20 | 20:46541995_T_C | 46541995 | 0.00089 | rs2426036 | T | C | 0.00406241 | 0.0129952 |
| 1 | 1:110625136_C_A | 110625136 | 9E-04 | rs139721115 | C | A | 0.0156077 | -0.0512803 |
| 5 | 5:99971418_A_G | 99971418 | 9E-04 | rs32283 | A | G | 0.00348478 | 0.0113763 |
| 6 | 6:52568342_T_C | 52568342 | 9E-04 | rs2207953 | T | C | 0.00377039 | -0.0123011 |
| 7 | 7:68109781_G_A | 68109781 | 9E-04 | rs2190068 | G | A | 0.00347154 | 0.0119546 |
| 11 | 11:4113200_A_G | 4113200 | 9E-04 | rs3750996 | A | G | 0.0046403 | -0.0146106 |
| 11 | 11:76383806_G_T | 76383806 | 9E-04 | rs947998 | G | T | 0.00373274 | 0.0116816 |
| 14 | 14:50493315_G_A | 50493315 | 9E-04 | rs145967741 | G | A | 0.0206403 | 0.0676626 |
| 1 | 1:84321948_A_G | 84321948 | 0.00091 | rs11163858 | A | G | 0.00345356 | 0.011486 |
| 5 | 5:180019952_T_C | 180019952 | 0.00091 | rs307804 | T | C | 0.00352589 | 0.0110746 |
| 6 | 6:27214789_A_C | 27214789 | 0.00091 | rs5030960 | A | C | 0.00430006 | -0.0142692 |
| 6 | 6:123101274_C_T | 123101274 | 0.00091 | rs74502222 | C | T | 0.00852659 | 0.0286592 |
| 11 | 11:119999191_A_G | 119999191 | 0.00091 | rs569044 | A | G | 0.00909987 | 0.0303382 |
| 12 | 12:31961708_C_T | 31961708 | 0.00091 | rs145409138 | C | T | 0.00447003 | 0.0151682 |
| 13 | 13:52492509_T_C | 52492509 | 0.00091 | rs17076102 | T | C | 0.0153205 | 0.0491318 |
| 19 | 19:46719360_C_T | 46719360 | 0.00091 | rs138976757 | C | T | 0.0122262 | -0.0416465 |
| 22 | 22:27973264_C_T | 27973264 | 0.00091 | rs13053396 | C | T | 0.00420627 | -0.0135208 |
| 1 | 1:234111442_C_T | 234111442 | 0.00092 | rs66540183 | C | T | 0.00364923 | -0.0117014 |
| 2 | 2:57729359_T_G | 57729359 | 0.00092 | rs2243947 | T | G | 0.00411095 | -0.0127426 |
| 2 | 2:103574137_T_C | 103574137 | 0.00092 | rs10202535 | T | C | 0.00345149 | -0.0117033 |
| 3 | 3:23624739_G_A | 23624739 | 0.00092 | rs117913304 | G | A | 0.00961862 | -0.0307556 |
| 4 | 4:110621820_A_G | 110621820 | 0.00092 | rs5030539 | A | G | 0.00337799 | 0.0110914 |
| 4 | 4:116723408_C_T | 116723408 | 0.00092 | rs2389090 | C | T | 0.00344144 | -0.0105719 |
| 7 | 7:68570963_T_C | 68570963 | 0.00092 | rs73146975 | T | C | 0.0101021 | -0.0316314 |
| 7 | 7:85012866_G_A | 85012866 | 0.00092 | rs148804680 | G | A | 0.0125325 | -0.0424647 |
| 17 | 17:6378481_T_C | 6378481 | 0.00092 | rs9905306 | T | C | 0.0116421 | -0.0395788 |
| 21 | 21:34081711_C_T | 34081711 | 0.00092 | rs113171118 | C | T | 0.00656154 | -0.0228852 |
| 1 | 1:227331000_G_A | 227331000 | 0.00093 | rs141830603 | G | A | 0.00822383 | 0.0272421 |
| 2 | 2:40297772_T_C | 40297772 | 0.00093 | rs11898088 | T | C | 0.00357773 | 0.0114956 |
| 2 | 2:79839050_T_G | 79839050 | 0.00093 | rs2195678 | T | G | 0.0092618 | 0.0320021 |
| 6 | 6:43240455_T_C | 43240455 | 0.00093 | rs937591 | T | C | 0.00337244 | -0.00996772 |
| 6 | 6:79637925_T_C | 79637925 | 0.00093 | rs10755377 | T | C | 0.00346749 | -0.0115684 |
| 7 | 7:862186_A_G | 862186 | 0.00093 | rs4721502 | A | G | 0.0033897 | 0.0116096 |
| 8 | 8:81921234_C_T | 81921234 | 0.00093 | rs4739780 | C | T | 0.00333465 | -0.0105676 |
| 10 | 10:79160265_T_G | 79160265 | 0.00093 | rs35809 | T | G | 0.00366749 | 0.011953 |
| 14 | 14:88632321_C_T | 88632321 | 0.00093 | rs2236267 | C | T | 0.00346572 | 0.0119176 |
| 14 | 14:99484879_G_A | 99484879 | 0.00093 | rs1565612 | G | A | 0.00355386 | 0.0116349 |
| 16 | 16:80903299_G_A | 80903299 | 0.00093 | rs148971944 | G | A | 0.0104923 | -0.031939 |
| 18 | 18:41209457_C_A | 41209457 | 0.00093 | rs9947502 | C | A | 0.00499689 | -0.0148534 |
| 1 | 1:237272980_A_G | 237272980 | 0.00094 | rs10925316 | A | G | 0.00332368 | 0.0108223 |
| 2 | 2:27963111_C_T | 27963111 | 0.00094 | rs190700662 | C | T | 0.0150958 | 0.0483549 |
| 2 | 2:218886044_T_C | 218886044 | 0.00094 | rs118155325 | T | C | 0.00992068 | -0.032518 |
| 4 | 4:16420363_G_A | 16420363 | 0.00094 | rs75429512 | G | A | 0.0141578 | -0.0480113 |
| 4 | 4:37202646_T_C | 37202646 | 0.00094 | rs149279856 | T | C | 0.0158813 | 0.0523221 |
| 13 | 13:39481254_T_C | 39481254 | 0.00094 | rs7989466 | T | C | 0.00936671 | 0.0313702 |
| 17 | 17:715782_G_A | 715782 | 0.00094 | rs7213222 | G | A | 0.00953177 | 0.0270962 |
| 18 | 18:55190400_A_G | 55190400 | 0.00094 | rs652140 | A | G | 0.00333671 | -0.0105276 |
| 1 | 1:37756668_G_T | 37756668 | 0.00095 | rs146492893 | G | T | 0.0203276 | 0.068454 |
| 5 | 5:20276361_C_T | 20276361 | 0.00095 | 5:20276361:C:T | C | T | 0.0110883 | -0.0359265 |
| 7 | 7:35706058_G_A | 35706058 | 0.00095 | rs7779075 | G | A | 0.00349842 | 0.0112126 |
| 9 | 9:4295281_T_C | 4295281 | 0.00095 | rs4741937 | T | C | 0.00766248 | 0.0244792 |
| 9 | 9:12198886_A_C | 12198886 | 0.00095 | 9:12198886:A:C | A | C | 0.0047071 | -0.0150121 |
| 11 | 11:122855298_T_G | 122855298 | 0.00095 | rs557790412 | T | G | 0.00792335 | -0.0277764 |
| 12 | 12:125864882_C_T | 125864882 | 0.00095 | rs11058155 | C | T | 0.00651577 | -0.0212455 |
| 15 | 15:22888255_G_A | 22888255 | 0.00095 | rs34456892 | G | A | 0.00940642 | -0.0319082 |
| 15 | 15:93891760_C_T | 93891760 | 0.00095 | rs1483319 | C | T | 0.00700346 | -0.0212254 |
| 16 | 16:8339919_A_G | 8339919 | 0.00095 | rs8056172 | A | G | 0.00350055 | -0.0115992 |
| 16 | 16:85988895_T_C | 85988895 | 0.00095 | rs6540235 | T | C | 0.00504809 | 0.0153442 |
| 17 | 17:1323019_C_T | 1323019 | 0.00095 | rs34673556 | C | T | 0.00482055 | -0.0160184 |
| 19 | 19:33079964_A_G | 33079964 | 0.00095 | rs78299806 | A | G | 0.00336825 | 0.0114247 |
| 1 | 1:18183424_G_A | 18183424 | 0.00096 | rs647619 | G | A | 0.00344364 | 0.0113774 |
| 2 | 2:20042295_G_A | 20042295 | 0.00096 | 2:20042295:G:A | G | A | 0.00371371 | 0.0124023 |
| 2 | 2:181449305_C_A | 181449305 | 0.00096 | rs2887180 | C | A | 0.00333252 | -0.0107818 |
| 5 | 5:78001356_C_A | 78001356 | 0.00096 | rs10041905 | C | A | 0.00340921 | -0.0106656 |
| 5 | 5:122034985_T_C | 122034985 | 0.00096 | rs10044811 | T | C | 0.0124197 | -0.0396601 |
| 7 | 7:78207959_G_A | 78207959 | 0.00096 | rs143416546 | G | A | 0.0169938 | 0.0517552 |
| 7 | 7:95595282_G_A | 95595282 | 0.00096 | rs2888983 | G | A | 0.00546401 | -0.0176185 |
| 9 | 9:123926863_C_T | 123926863 | 0.00096 | 9:123926863:C:T | C | T | 0.0104456 | -0.0366989 |
| 11 | 11:78396661_C_A | 78396661 | 0.00096 | rs147436170 | C | A | 0.0163982 | -0.0520617 |
| 16 | 16:6378086_C_T | 6378086 | 0.00096 | rs56371995 | C | T | 0.00448009 | -0.0149279 |
| 16 | 16:79504724_C_T | 79504724 | 0.00096 | rs1077055 | C | T | 0.00645933 | -0.0204828 |
| 17 | 17:36899045_C_T | 36899045 | 0.00096 | rs3785457 | C | T | 0.00337218 | 0.0109094 |
| 2 | 2:131753007_A_G | 131753007 | 0.00097 | rs72614457 | A | G | 0.00425242 | 0.0137006 |
| 5 | 5:82497764_G_A | 82497764 | 0.00097 | rs112218000 | G | A | 0.00682261 | -0.0221842 |
| 5 | 5:121418602_C_T | 121418602 | 0.00097 | rs840463 | C | T | 0.0135369 | 0.0414525 |
| 5 | 5:127557814_T_C | 127557814 | 0.00097 | rs11955148 | T | C | 0.00404207 | -0.0135397 |
| 8 | 8:20466541_T_C | 20466541 | 0.00097 | rs13249563 | T | C | 0.00391888 | -0.0125718 |
| 11 | 11:1956365_G_A | 1956365 | 0.00097 | rs585088 | G | A | 0.00485611 | -0.0170097 |
| 13 | 13:45448353_A_G | 45448353 | 0.00097 | rs76627386 | A | G | 0.0101795 | -0.0326148 |
| 15 | 15:44648044_G_T | 44648044 | 0.00097 | rs142878670 | G | T | 0.011603 | 0.0379058 |
| 20 | 20:47336184_T_C | 47336184 | 0.00097 | rs117253294 | T | C | 0.016221 | -0.0567649 |
| 4 | 4:22513052_T_C | 22513052 | 0.00098 | rs73114194 | T | C | 0.0035751 | 0.0115177 |
| 4 | 4:81280233_G_A | 81280233 | 0.00098 | rs190771863 | G | A | 0.0178808 | -0.0572358 |
| 4 | 4:137897645_C_T | 137897645 | 0.00098 | rs182127754 | C | T | 0.0152654 | 0.0501462 |
| 10 | 10:19935608_A_G | 19935608 | 0.00098 | rs11010907 | A | G | 0.00344772 | 0.0120355 |
| 10 | 10:125016364_C_T | 125016364 | 0.00098 | rs77929588 | C | T | 0.00930668 | 0.0305335 |
| 11 | 11:113803008_T_C | 113803008 | 0.00098 | rs11214773 | T | C | 0.00911728 | -0.0293134 |
| 12 | 12:31197341_T_C | 31197341 | 0.00098 | rs35025 | T | C | 0.00342336 | -0.0103649 |
| 12 | 12:79581503_G_A | 79581503 | 0.00098 | rs7350564 | G | A | 0.00333624 | 0.0113648 |
| 13 | 13:54219420_C_T | 54219420 | 0.00098 | rs9591527 | C | T | 0.00390943 | 0.0122398 |
| 18 | 18:9806466_C_T | 9806466 | 0.00098 | rs116523877 | C | T | 0.00820589 | -0.0288136 |
| 18 | 18:45787494_G_A | 45787494 | 0.00098 | rs11873801 | G | A | 0.00406136 | -0.0130922 |
| 21 | 21:25892954_G_A | 25892954 | 0.00098 | rs62215720 | G | A | 0.0158117 | 0.048576 |
| 1 | 1:6502107_T_C | 6502107 | 0.00099 | rs75242862 | T | C | 0.0120732 | -0.0374585 |
| 1 | 1:233719603_T_C | 233719603 | 0.00099 | rs4620469 | T | C | 0.00354278 | 0.0125355 |
| 4 | 4:15826558_C_T | 15826558 | 0.00099 | rs1800561 | C | T | 0.0143403 | -0.0483288 |
| 5 | 5:429989_G_A | 429989 | 0.00099 | rs957792 | G | A | 0.0064401 | 0.0224132 |
| 6 | 6:10463611_C_A | 10463611 | 0.00099 | rs145105753 | C | A | 0.017089 | 0.0577627 |
| 8 | 8:123870465_C_T | 123870465 | 0.00099 | rs16897563 | C | T | 0.00348094 | 0.0113872 |
| 11 | 11:46333235_G_T | 46333235 | 0.00099 | rs117246933 | G | T | 0.00747119 | -0.0242599 |
| 14 | 14:73118921_A_C | 73118921 | 0.00099 | rs181670708 | A | C | 0.0120719 | 0.0405 |
| 14 | 14:95850409_G_A | 95850409 | 0.00099 | rs10144978 | G | A | 0.00463018 | -0.0150613 |
| 15 | 15:34214815_G_A | 34214815 | 0.00099 | rs141982178 | G | A | 0.0121288 | 0.0392106 |
| 16 | 16:10625061_C_A | 10625061 | 0.00099 | rs181262515 | C | A | 0.0156562 | -0.0501533 |
| 17 | 17:9860265_G_A | 9860265 | 0.00099 | rs78025230 | G | A | 0.0071035 | -0.0229748 |

CHR, chromosome; SNP, single nucleotide polymorphism; BP, base pair position on the genome GRCh37; P, P-values; rsid, reference NP luster ID; REF, reference; ALT, alternative effect allele; SE, standard error of the effect size estimate; BETA, estimated effect size of the alternative allele
